# Proteomic analyses reveal distinct chromatin-associated and soluble transcription factor complexes

**DOI:** 10.15252/msb.20145504

**Published:** 2015-01-21

**Authors:** Xu Li, Wenqi Wang, Jiadong Wang, Anna Malovannaya, Yuanxin Xi, Wei Li, Rudy Guerra, David H Hawke, Jun Qin, Junjie Chen

**Affiliations:** 1 Department of Experimental Radiation Oncology, The University of Texas MD Anderson Cancer CenterHouston, TX, USA; 2 Department of Molecular and Cellular Biology, Dan L. Duncan Cancer Center, Baylor College of MedicineHouston, TX, USA; 3 Division of Biostatistics, Dan L. Duncan Cancer Center, Baylor College of MedicineHouston, TX, USA; 4 Department of Statistics, Rice UniversityHouston, TX, USA; 5 Proteomics Facility, Department of Pathology, The University of Texas MD Anderson Cancer CenterHouston, TX, USA

**Keywords:** forkhead box, mass spectrometry, protein–protein interaction, transcriptional factor

## Abstract

The current knowledge on how transcription factors (TFs), the ultimate targets and executors of cellular signalling pathways, are regulated by protein–protein interactions remains limited. Here, we performed proteomics analyses of soluble and chromatin-associated complexes of 56 TFs, including the targets of many signalling pathways involved in development and cancer, and 37 members of the Forkhead box (FOX) TF family. Using tandem affinity purification followed by mass spectrometry (TAP/MS), we performed 214 purifications and identified 2,156 high-confident protein–protein interactions. We found that most TFs form very distinct protein complexes on and off chromatin. Using this data set, we categorized the transcription-related or unrelated regulators for general or specific TFs. Our study offers a valuable resource of protein–protein interaction networks for a large number of TFs and underscores the general principle that TFs form distinct location-specific protein complexes that are associated with the different regulation and diverse functions of these TFs.

See also: Z Ji & AD Sharrocks (January 2015)

## Introduction

**Figure 1:**
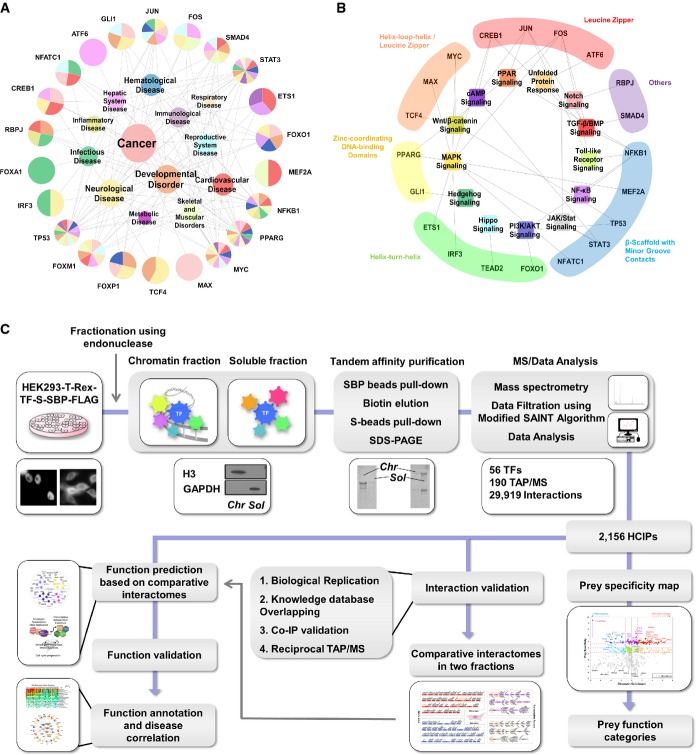
Proteomic analysis of human transcription factors

Over the years, considerable effort has been devoted to understand the signalling pathways, the basis of biological activities in all living organisms. Sophisticated signal transduction pathways are required for the development and survival of any organism, a minor disruption of which may cause developmental defects and diseases such as cancer (Fig1A). The examples of these highly conserved signalling pathways include the Wnt (MacDonald *et al*, 2009), TGF-β (Massague, 1998) and NF-κB (Hayden & Ghosh, 2004) pathways. Many of these pathways function by ultimately regulating the activity of certain transcription factors (TFs), often by changing their localizations. Reports on individual proteins suggested that the chromatin association of TFs is tightly controlled by upstream signals. For example, NF-κB is known to translocate from the cytoplasm to the nucleus upon activation, which is a critical step coupling extracellular stimuli with transcriptional activation (Baldwin, 1996).

Disease correlation of 19 TFs and 4 well-studied FOX family members, based on their GO annotations. Each colour indicates one disease. The size of each coloured pie indicates the relative ratio of –log (*P*-value) of GO annotations in the corresponding disease.

Pathway correlation and structural superclasses of TFs. Each coloured area indicates one superfamily.

Schematic diagram showing the major steps involved in TAP/MS screening and data analysis of human TFs and snapshot for each part of the data. Fifty-six transcription factors, together with 70 unrelated control proteins and control vector, were constructed into a vector harbouring a C-terminal SFB-tag through gateway technology. 293T cells stably expressing each bait protein were generated by stable transfection and puromycin selection. Protein was collected and separated into two fractions by a two-step lysis process. Through the standard tandem affinity purification steps, purified protein complexes were identified by mass spectrometry analysis, and final interacting proteins were generated by SAINT algorithm-based filtration. The data were subjected to prey functional categories analysis, interaction validation and function validation. Snapshots of data generated by each step were shown aside.

TFs are known to be rigorously regulated via their associations with other proteins (Blackwood & Eisenman, 1991). However, while the DNA-binding and the transcriptional activities of TFs on chromatin have been extensively studied, our knowledge of protein–protein interactions (PPIs) that may occur off the chromatin, which are important for the regulations and functions of these TFs, is very limited. Knowing what proteins TFs interact with and, especially, where they interact will greatly improve our understanding of how the activities of these TFs are controlled.

One example is the Forkhead box (FOX) family of TFs, which has been relatively well studied in their regulations of transcriptional activities, but little is known about protein–protein interactions involving these TFs. The term “Forkhead” was derived from a mutant *Drosophila melanogaster* that has a forklike head (Weigel *et al*, 1989). They have been classified into 19 subfamilies on the basis of the conservation of their DNA-binding domains (Kaestner *et al*, 2000). However, some have since been found to be variants of other family members. To date, there are 40 experimentally confirmed FOX family members and additional six or more FOX-like proteins in humans. Several *in vitro* studies have identified the consensus DNA sequences of a few FOX proteins, including those of FOXA, FOXD, FOXO, FOXP and FOXM, and their target genes using microarrays and ChIP-sequencings (Jolma *et al*, 2013). For example, the consensus sequence for FOXA1 is 5′-(G/A)(T/C)(A/C)AA(C/T)A-3′ (Georges *et al*, 2010), and FOXA1 plays a pivotal role in ER transcription activities (Hurtado *et al*, 2011).

FOX family TFs play important roles in regulating the expression of genes involved in a sundry of cellular processes, especially during development and tumorigenesis (Benayoun *et al*, 2011; Lam *et al*, 2013). Many FOX family members have been reported to be involved in cell, tissue and organ developments. For example, FOXA family members are required for normal development of liver, pancreas, lungs and prostate. Mutations or deletions of FOX genes often lead to developmental defects (Tuteja & Kaestner, 2007a,b), including severe organ and immune defects, premature ovarian failure, mental retardation, autism and speech disorders (Carlsson & Mahlapuu, 2002; Lehmann *et al*, 2003; Ariani *et al*, 2008; Hamdan *et al*, 2010). Recent studies indicated that the FOX family is involved in tumorigenesis as well (Lehtinen *et al*, 2006; Anders *et al*, 2011; Kress *et al*, 2011; Sykes *et al*, 2011; Ross-Innes *et al*, 2012). For example, Akt promotes cell survival by phosphorylating and inhibiting FOXO transcriptional activity (Brunet *et al*, 1999), which is important for the development of leukaemia and colorectal cancer (Tzivion *et al*, 2011). This regulation is achieved by changing the localization of FOXOs, since Akt-dependent phosphorylation of FOXOs promotes the relocalization of FOXOs to the cytoplasm via enhancing the binding of FOXOs to 14-3-3 family proteins and thereby inhibiting their transcriptional activities (Brunet *et al*, 1999). FOXM1 is often involved in the oncogenesis of many different types of carcinoma (Koo *et al*, 2012). FOXM1 transcriptional activities are required for its oncogenic functions (Lam *et al*, 2013); however, FOXM1 could also promote β-catenin nuclear translocation independently of its DNA-binding activity, which may play a role in glioma formation (Zhang *et al*, 2011). These examples provide a rationale to further determine how these FOX proteins are regulated by protein–protein interaction network.

Thus, we started this project using the FOX TF family as a model to better understand how TFs in general are regulated on and off DNA. We used tandem affinity purification (TAP) followed by mass spectrometry (MS) analysis. As an unbiased approach, MS offers tremendous advantages over other methods in identifying PPIs under near-physiological conditions. Several large-scale MS-based studies have been conducted with yeast and human co-regulator protein complexes (Gavin *et al*, 2006; Malovannaya *et al*, 2011). In addition, several function-related large-scale studies have been conducted, which focus on specific signalling pathways (Behrends *et al*, 2010) or biological processes (Matsuoka *et al*, 2007; Bennett *et al*, 2010). While current methods are effective in identifying stable protein complexes, they are inadequate in recognizing regulated interactions, which are essential for understanding the complex signalling networks in the cell. This shortcoming is especially obvious when conducting large-scale proteomics analysis, since the appearance of abundant associated proteins in the interactomes drastically reduces the sensitivity for detecting small amounts of regulated but biologically significant interactions. Using a modified TAP/MS method, we have identified several relatively weak but regulated interactions for individual proteins and functionally validated these interactions (Liu *et al*, 2010; Wang *et al*, 2013). Thus, it is technically feasible to identify these functionally important interactions. The question is whether or not we can expand these studies to a relatively larger scale, with the ultimate goal of studying how PPIs change under different physiological conditions. We chose to start by revealing PPIs of TFs that are present on or off chromatin, since we believe that these location-specific PPIs are likely to be engaged in the differential regulations of these TFs.

We isolated soluble versus chromatin fractions based on our assumption that TFs on chromatin are likely to be involved in transcription-related functions, while they are not when in the soluble fractions. Most TFs localize constantly in the nucleus as determined by immunostaining experiments. It was assumed that these TFs would be chromatin bound all the time. This is not the case, since even for TFs that are always localized in the nucleus, they are often present in both soluble and chromatin fractions. Excitingly, our initial proteomics study of the FOX family of TFs revealed that they indeed form distinct complexes on and off chromatin. This finding may not be a total surprise, but it had never been systematically demonstrated. We wondered whether this is a general phenomenon that would apply to other TFs as well. Thus, we performed TAP/MS analyses for 19 non-FOX TFs involved in a variety of pathways associated with development and cancer. These non-FOX TFs are from five structural TF superfamilies to exclude the potential bias caused by the structural preference of their DNA-binding activities. With these additional TFs, we still observed distinct complexes of these TFs formed on and off chromatins. Altogether, our study provided location-specific (i.e. chromatin-associated and chromatin-free) complexomes for 56 TFs involved in various signalling pathways and validated our working hypothesis that TFs are engaged in different PPIs on and off chromatin, which are likely important for their regulations and diverse functions.

## Results

### Proteomic analysis of transcription factors

We performed TAP/MS analyses for total 56 TFs (Supplementary Table S1), including 37 FOX family members and 19 non-FOX TFs involved in various human diseases (Fig1A) and signalling pathways (Fig1B, Supplementary Table S1) (i.e. MYC, MAX, TP53, NFKB1, JUN, FOS, SMAD4, TEAD2, RBPJ, TCF4, ATF6, CREB1, ETS1, GLI1, IRF3, MEF2A, NFATC1, PPARG and STAT3). These factors covered the five structural superclasses of TFs (Matys *et al*, 2006), which include basic domain TFs (leucine zippers: ATF6, CREB1, FOS, JUN; helix-loop-helix factors: MYC, MAX, TCF4), zinc-coordinating DNA-binding domains TFs (GLI1, PPARG), helix-turn-helix TFs (ETS1, IRF3, TEAD2, FOX), β-scaffold factors with minor groove contacts (MEF2A, NFATC1, NFKB1, STAT3, TP53) and others (RBPJ, SMAD4).

We established HEK293T-derivative cell lines stably expressing streptavidin-S-FLAG (SFB) triple-tagged TFs by transient transfections followed by puromycin selection. We picked 12–24 single clones for each bait, examined them by Western blotting and immunostaining and chose the ones with the correct subcellular localizations and the lowest expression for affinity purifications. We compared the immunostaining results of our stable cell lines with those available in the literature. All of the tagged proteins were localized as previously reported (Supplementary Fig S1A and summarized in Supplementary Table S2). We also compared the expression levels of 12 tagged proteins with endogenous proteins in our stable cell lines by Western blotting. Most of the tagged proteins were expressed similar to or slightly higher than that of endogenous proteins (Supplementary Fig S1B).

To assess specific protein complexes of TFs on and off chromatin, we first isolated soluble fractions using a crude lysis step and then treated the insoluble pellets (i.e. the chromatin fraction) with TurboNuclease, which hydrolyses both single- and double-stranded DNA or RNA to oligonucleotides of 1–4 bases in length, to release chromatin-bound proteins. We detected very little histones, HMG proteins and other chromatin components in our purifications, suggesting that we were able to eliminated most of the non-specific interactions mediated by DNA. We performed TAP/MS using both soluble and chromatin fractions (for examples, see Supplementary Fig S1C; for fractionation specificity, see Supplementary Fig S1D) and then conducted data analysis to identify these location-specific interactions (i.e. interactions on or off chromatin; Fig1C). We performed a total of 120 experiments and biological replicates for 24 of them (20%). We also included 70 control purifications, which comprise 61 unrelated protein purifications and nine vector only purifications. The vector-only purifications contain four in chromatin fraction, four in soluble fraction and one combined.

**Figure 2:**
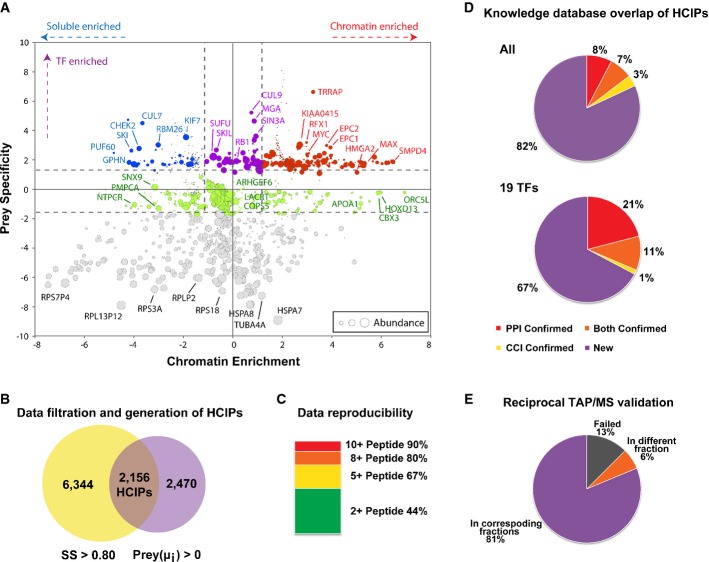
Proteomic analysis of human transcription factors and data validation

We identified a total of 29,919 interacting proteins from 120 TF and 70 control TAP/MS, which represents 3,751 unique preys (Supplementary Tables S3 and S4). To classify these preys, we adopted a significance analysis method for spectral count data (Choi *et al*, 2008, 2011) and assigned each prey appearing in the TF group (3,714 in total) with an abundance score and specificity score. The prey abundance score was a parameter estimated by the Poisson mixture model using the SAINT algorithm, which reflects the estimated protein abundance across all the experiments. The specificity score was another parameter, which represents the difference of the estimated prey abundance between the negative control group and the entire group (sample + control). We plotted these two scores to get the prey proteins specifically enriched in the sample group (Supplementary Fig S2A). We have removed the bait self-identifications to avoid any interference of data analysis due to bait overexpression. Using the same approach, we also obtained the prey specificity scores for proteins identified in chromatin (Supplementary Fig S2B) and soluble fractions (Supplementary Fig S2C). These analyses allowed us to easily rule out the generic preys (HSPs) and other non-specific binding proteins (actin, tubulin, RPLs), which appear as tails at the lower right corner of each atlas, while the proteins with high specificity and abundance appear in the upper right regions (Supplementary Fig S2). We then combined these three individual distributions into a bubble plot for prey categorization (Fig2A, Supplementary Table S5). Using this prey fraction and specificity distribution map, we classified the preys into four coloured groups based on their positions: red: co-regulators of TFs may be involved in transcriptional regulation (e.g. MAX, TRRAP); purple: regulators with no fractional preference (MGA, SIN3A); blue: transcription-unrelated functions or negative regulators of TFs (CUL7, SKI); and green: potential regulators with less specificity (CBX3, COPS5, NTPCR) (Fig2A). The grey indicates non-specific binding proteins with no preference (HSP, RPS, RPL), which locate at the bottom of this plot (Fig2A). This prey distribution map may suggest how these preys act in regulating TFs in a generic or a specific manner.

Comparative analysis of prey specificities of TFs over different fractions. The *y*-axis shows TF-binding specificities of preys: positive, specifically associate with transcription factors group; negative, no binding preference. The *x*-axis depicts fraction specificities of preys: positive and negative numbers indicate preference for their enrichment in chromatin and soluble fractions, respectively. The size of a coloured bubble indicates the log (overall abundance) of individual preys. The selected preys were categorized into four groups based on their positions highlighted with different colours: red, specific co-regulators of TFs that may be involved in transcriptional regulation; purple, regulators with no fractional preference; blue, transcription-unrelated functions or negative regulators of TFs; green, potential regulators with less specificity; plus a group of abundant proteins with no binding preference, which were shown at the bottom of the map (grey).

Data filtration using a modified SAINT algorithm. The total peptide and protein numbers obtained from mass spectrometry analysis are listed. The SAINT score > 0.80 was used as the cut-off to identify HCIPs, as suggested by the SAINT method. We also applied another filtration using the prey information in 70 control purifications to remove the non-specific bindings or contaminants. The numbers of HCIPs remaining after these two filtrations are shown here.

Data reproducibility test based on biological replicates. The HCIP overlap ratio rises with the peptide numbers.

Summary of HCIPs that overlap with those reported in knowledge PPI databases. 389 interactions of the total 2,156 HCIPs (˜18%) were reported previously.

Summary of reciprocal purifications of 16 interactions identified from MAX, NFATC1, RBPJ and CREB1 purifications performed with the same TAP/MS protocol. 14 out of 16 preys captured their corresponding baits from reciprocal AP/MS, 13 of which are in the corresponding fractions.

To discover individual interactions with high confidence (HCIPs, high-confident candidate interacting proteins), raw data from the MS analysis were subjected to a modified SAINT (Significance Analysis of INTeractome) algorithm (Choi *et al*, 2011; Wang *et al*, 2014) (Fig2B). We used a two-pool analysis, and the spectra counts from TF group and control group were assembled as a matrix for all of the bait and prey proteins (Supplementary Table S3). In total, 29,919 protein matches were identified in 190 experiments, with 60 TF purifications from chromatin fractions, 60 TF purifications from soluble fractions and 70 control purifications (Fig2C). We temporarily removed the bait self-identifications from the list to get a better estimation of bait abundance in cells and then added them back after the completion of the filtration. According to the SAINT methodology, the interactions with over 0.8 probability score were kept for further analysis as described below. 8,500 interactions passed this first filtration: 3,927 of which from chromatin fractions and 4,573 from soluble fractions (Fig2C).

We then used the prey specificity information to further eliminate contaminants frequently shown in our purifications. The score *μ*_*i*_ of individual preys as common contaminants were used to calculate the probability of abundant or non-specific preys that frequently showed up in these purifications. We filtered out preys with *μ*_*i*_ *≥ 0*, which removed common contaminants and/or abundant non-specific binding proteins; 4,626 interactions passed this filtration. In total, 2,156 interactions passed both filtrations (Fig2B) and were designated as HCIPs: 1,423 of which from chromatin fractions and 733 from soluble fractions (Table1, Supplementary Table S6, Supplementary Dataset S1). We also performed biological replicates for 24 purifications, and the overlapped HCIPs were summarized in Supplementary Table S7. As predicted, the reproducibility of the HCIPs increases with the spectra counts (44% for 2 counts, 67% for 5 counts and 90% for 10 counts) (Fig2C).

**Table 1:** Summary of step-by-step proteomics data analysis.

	Total	TFs	Chromatin	Soluble	Controls
Experiments	190	120	60	60	70
Peptides	185,394	113,654	48,077	65,577	71,740
Proteins	29,919	19,698	9,380	10,318	10,221
Passed SAINT	8,500	8,500	3,927	4,573	
HCIP	2,156	2,156	1,423	733	

The identified proteins and peptide numbers of each group and step were presented.

### Data validation and functional studies

To validate the reliability of our data set, we searched the HCIPs in various PPI databases, including BioGrid (Stark *et al*, 2006), STRING (von Mering *et al*, 2003), BIND (Bader *et al*, 2003), DIP (Xenarios *et al*, 2000), HPRD (Prasad *et al*, 2009) and *C. elegans* TF data set (Reece-Hoyes *et al*, 2013). 15% interactions we identified have been confirmed by this combined knowledge database. We also compared the results with the CCI data set we published early (Malovannaya *et al*, 2011). 10% interactions we identified have been confirmed by the CCI database, which was created based on MS analysis of immunoprecipitates of endogenous protein complexes and therefore could be considered as an “endogenous co-IP validation”. For example, proteins SIN3A, SAP130, ARID4B, MORC2, FOXK1 and FOXK2 from MAX purification have been confirmed by the CCI database (Supplementary Table S6 “CCI-Confirmed” column). In total, 389 interactions of the total 2,156 HCIPs (∽18%) were reported previously (Fig2D and Supplementary Table S6). If only considering the 19 relatively well-studied non-FOX TFs, 33% (217 out of the 663 HCIPs) of the interactions were reported previously (Fig2D and Supplementary Table S6), which confirmed the validity of our data set. We have also overlapped our results with the CRAPome, a collection of common contaminants in AP/MS data (Mellacheruvu *et al*, 2013). Using 20% frequency as the “non-specific interaction” cut-off, we found our HCIP set only generated 3.8% “potential false positives”.

To experimentally verify our proteomics data set (Supplementary Table S6), we performed reciprocal purifications using prey proteins and hoped to identify the corresponding bait proteins. We conducted reverse purifications for MAX-associated protein L3MBTL2, E2F6, FOXK2; NFATC1-associated protein JUN, HOXD13, CREB1, ATF1, ATF3; RBPJ-associated protein L3MBTL3, KDM1, FBXO42; and CREB1-associated protein ATF1, HMGA1, ZNF131, NFIX, NFATC2. In 14 out of 16 purifications, we identified the corresponding baits in the reverse purifications (Fig2E, Table2), 13 of which were in the corresponding fractions. This further validated that our purification results reflect endogenous PPIs under physiological conditions. We also performed co-IP experiments using tagged preys to pull down endogenous baits, to validate the HCIPs of MAX, FOXM1 and FOXO3. We have obtained 54% positive rate (82% if only counting the preys we actually tested since some prey constructs were not available) (Supplementary Fig S3A, B and D). shRNA screening of MAX and FOXO3 HCIPs was conducted using, respectively, Ki67 staining and GADD45A mRNA level as read-outs. We have obtained eight potential positive regulators and six potential negative regulators of MAX, and six potential positive regulators of FOXO3 (Supplementary Fig S3C and E). From FOXO3 positive hits, we chose FOXK1 for further validation. We found that FOXK1 also regulates FOXO3 subcellular localization. Overexpression of FOXK1 translocated FOXO3 to the nucleus, while knocking down FOXK1 reduced the FOXO3 nuclear translocation upon treatment with PI3K inhibitor LY294002 (Supplementary Fig S3F). Taken together, these data indicate that the interactomes we built are highly reliable.

**Table 2:** Summary of reciprocal purifications results.

Bait	Prey	In bait fraction	In prey fraction	Bait	Prey	In bait fraction	In prey fraction
MAX	L3MBTL2	Chr	Sol	RBPJ	L3MBTL2	Chr	Sol
MAX	E2F6	Chr	Chr	RBPJ	KDM1	Chr	Chr
MAX	FOXK2	Chr	Chr	RBPJ	FBXO42	Chr/Sol	Chr/Sol
NFATC1	JUN	Chr	Chr	CREB1	ATF1	Chr/Sol	Chr/Sol
NFATC1	HOXD13	Chr	Chr	CREB1	HMGA1	Chr	N
NFATC1	CREB1	Chr	Chr	CREB1	ZNF131	Chr	N
NFATC1	ATF3	Chr	Chr	CREB1	NFIX	Chr	Chr
NFATC1	ATF1	Chr	Chr	CREB1	NFATC2	Chr	Chr

Reciprocal purifications of 16 interactions identified from MAX, NFATC1, RBPJ and CREB1 purifications were performed with the same TAP/MS protocol. Chromatin and soluble fractions were separated and whether the corresponding baits appeared in the reciprocal purification was indicated by fraction name or “N”.

We have also searched post-translational modifications including phosphorylation and acetylation in all our MS results (Supplementary Table S8) and identified 8,043 peptides modified by phosphorylation and/or acetylation. In total, we identified 6,842 phosphorylation sites and 4,384 acetylation sites. Among these modified peptides, 47% of total and 36% of the bait peptides only exist in one fraction, which indicates that the PTMs of TF protein complexes are different in soluble and chromatin fractions.

### Transcription factors form distinct functional complexes on and off chromatin, which could be functionally relevant

**Figure 3:**
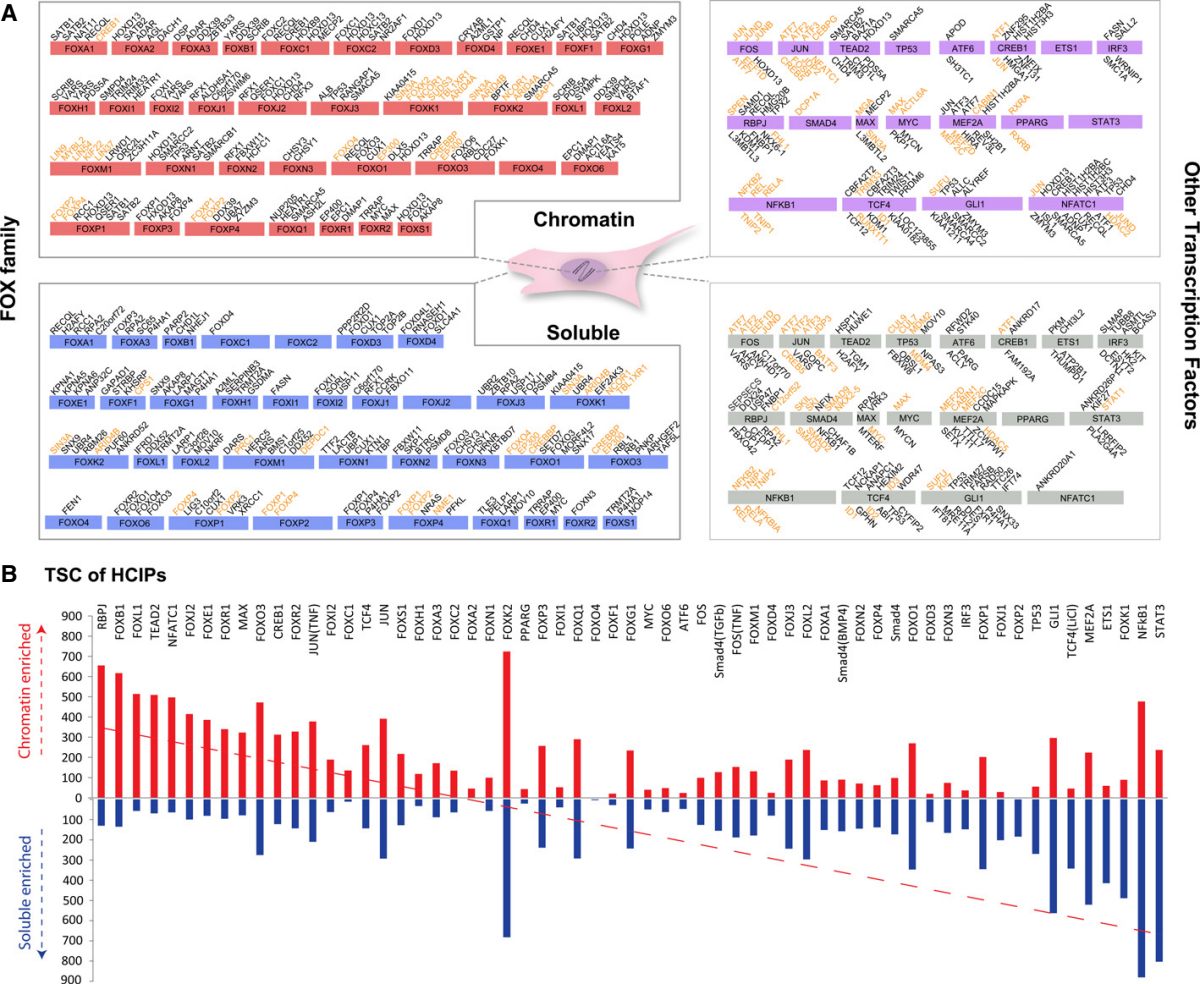
Transcription factors form distinct complexes on and off chromatin

We listed the top HCIPs of each bait in each fraction (Fig3A). It is clear that these HCIPs are very different between the fractions. The total spectra counts (TSC) of HCIPs for different baits were also compared between two fractions, which reflect the total amount of specific protein bindings of these TFs on or off chromatin (Fig3B), which showed distinct preferences for these TFs to form complexes on or off the chromatin. RBPJ, a TF known to be chromatin bound before and after Notch activation, showed overwhelming protein binding in the chromatin fraction, while STAT3, which is predominantly present in the cytoplasm without stimuli, had very few interactions in the chromatin fraction (Fig3B). These results indicate that our comparative interactomes are biologically meaningful.

HCIPs with highest spectra counts were listed. The length of each box with the protein name on it indicates the protein size. Black fonts indicate new interactions identified by our purifications. Orange fonts indicate interactions defined by our purifications and the literature.

Total spectra counts of TFs in different fractions. The *y*-axis indicates the total spectra counts (TSC) of HCIPs in corresponding TF purifications. Red bar: TSC of HCIPs in chromatin fractions; blue bar: TSC of HCIPs in soluble fractions.

**Figure 4:**
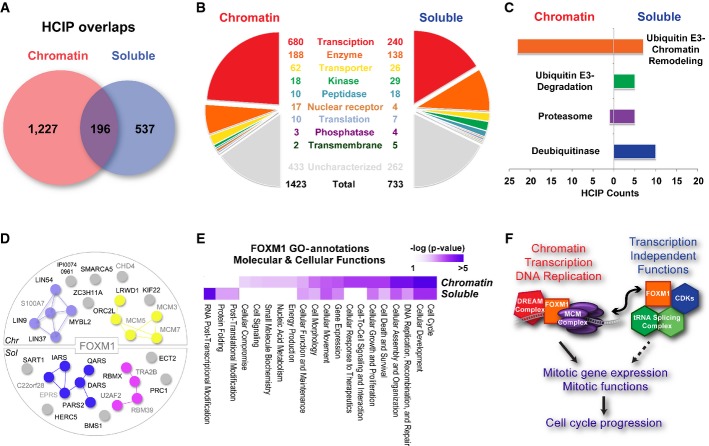
Protein categories and functions are different on and off chromatin

Notably, only 14% of total HCIPs appeared in both soluble and chromatin fractions (Fig4A), many of which are bait self-identification. To make sure that this is not due to any artefact caused by data analysis, we used the CCI algorithm (Malovannaya *et al*, 2011) and an in-house written algorithm based on CompPASS (Sowa *et al*, 2009). The percentage of total HCIPs appearing in both fractions varies between 8 and 14% in these analyses, which confirmed our working hypothesis that TFs are differentially regulated on and off chromatin by distinct protein partners. We listed the protein families of these HCIPs (Supplementary Table S6) and summarized them in two different fractions (Fig4B). As expected, proteins related with transcription are enriched in the chromatin fraction (*P *= 4.07e-7), while kinase (*P *= 6.08e-5), peptidase (*P *= 7.16e-4) and transmembrane proteins (*P *= 0.037) are enriched in the soluble fraction. This may reflect that the regulation of protein post-translational modifications and trafficking occur preferentially in the soluble fraction. Since these functional indications based protein families may not be precise, we further annotated the HCIPs with ubiquitin-related function categories (Fig4C). We found that degradation-related ubiquitin (Ub) E3 ligases/F-box proteins (FBXs), proteasome subunits and deubiquitinase (DUBs) only appeared in soluble fractions, while the chromatin remodelling ubiquitin E3s are highly enriched in chromatin fractions (Fig4C). This more detailed analysis indicates that fraction preference is associated with the functions and/or the regulations of prey proteins.

Overlap Venn diagram of HCIPs in chromatin and soluble fractions. Only 196 HCIPs appear in both chromatin and soluble fractions, 120 of which are bait self-identifications.

Function categories of HCIPs in the two fractions.

Ubiquitin-related HCIPs enrichment in the two fractions. The *y*-axis indicates the number of HCIPs.

FOXM1 HCIPs form distinct complexes in chromatin versus soluble fractions. HCIPs with highest spectra counts or previously known associated proteins were listed. Black font indicates the HCIPs defined by our purifications; grey font indicates the proteins defined by our purifications that form complexes with HCIPs, but are not in the HCIP list. Lines indicate the interactions defined in the literature. Prey dots in different colours indicate different function complexes defined in the literature.

GO annotation of FOXM1 in molecular and cellular functions based on its HCIPs identified in chromatin or soluble fractions. Colours indicate the –log (*P*-value) of GO annotations.

A model showing on/off chromatin functions of FOXM1 in mitosis and cell cycle progression. All of the components indicated were identified from FOXM1 purifications.

Different complexes formed with a given TF on and off chromatin may indicate how it is regulated differently and/or carry out distinct functions in these fractions. For instance, the DREAM complex, which is composed of LIN9, LIN37, LIN54, MYBL2 and S100A7, was identified only in the chromatin fraction of FOXM1 (Fig4D). This finding agrees with the known function of the DREAM complex, which recruits FOXM1 to promote mitotic gene expression (Sadasivam *et al*, 2012; Chen *et al*, 2013; Grant *et al*, 2013) and thereby regulates cell cycle progression (Litovchick *et al*, 2007; Schmit *et al*, 2007). On the other hand, the tRNA splicing ligase complex, which is composed of IARS, DARS, EPRS, PARS2, QARS and C22orf28, was present only in the soluble fraction of FOXM1-associated proteins. This complex is known to be phosphorylated during mitosis and is required for tRNA splicing and mitotic functions (Dephoure *et al*, 2008; Olsen *et al*, 2010), implying that FOXM1 may have a previously unknown function in this process. The GO function annotation suggested that FOXM1 may have distinct functions on and off the chromatin, but still contribute to the overall functions of cell cycle, cellular development, cell growth and proliferation (Fig4E). These data suggest that there might be a potential role of FOXM1 in tRNA splicing complex function or regulation. Our GO analysis of FOXM1 interactomes suggested similar biological functions of FOXM1 with different complex formations in these two fractions (Fig4F).

**Figure 5:**
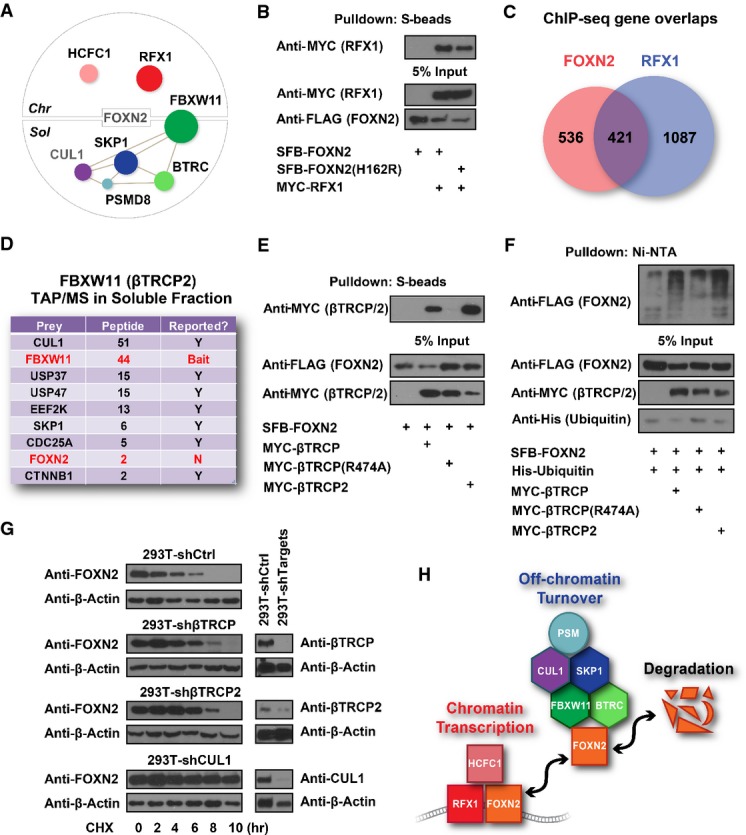
Functional validation of FOXN2 based on its interacting proteins in soluble and chromatin fractions

In some other cases, the complexes in the two fractions may perform unrelated or even opposing regulatory roles. Many ubiquitin (Ub) E3 ligases/F-box proteins (FBXs) and proteasome subunits appeared in the left region of our prey specificity and abundance map (Fig2A) and enriched in the soluble fraction (Fig4C), which indicated they may serve as general negative regulators of TFs. Several of these interactions of Ub-related proteins with TFs, for example MDM2/MDM4 and TP53 interaction, are well studied, but most are not. We picked FOXN2 as an example to validate the potential negative regulation of TFs by E3 ubiquitin ligases. We found that FOXN2 binds to RFX1, a known TF or transcription cofactor that also helps other TFs to recognize target DNA (Gajiwala *et al*, 2000), in the chromatin fraction (Fig5A). We confirmed this interaction via co-IP experiments (Fig5B) and found 44% FOXN2 target genes overlapped with RFX1 target genes (Fig5C). This indicates that FOXN2 and RFX1 form a transcriptional complex on chromatin and co-regulate a subset of gene transcription. On the other hand, FOXN2 binds to SKP1, βTRCP (also called BTRC/FBXW1), βTRCP2 (also called FBXW11) and some proteasome components such as PSMD8 in the soluble fraction. We have also identified CUL1 from our parallel virus-based FOXN2 TAP/MS. Indeed, our pathway annotation analysis indicated that FOXN2 is tightly associated with the protein ubiquitination pathway in the soluble fraction, but not in the chromatin fraction. To further validate these results, we performed reciprocal purification in HEK293T cells using βTRCP2/FBXW11 as the bait, which is the adaptor protein that should associate with CUL1 substrates (Lyapina *et al*, 1998; Wu *et al*, 2000). Indeed, we identified many known βTRCP2-interacting proteins, which include CUL1, SKP1 (Lyapina *et al*, 1998; Wu *et al*, 2000), USP37 (Burrows *et al*, 2012), USP47 (Peschiaroli *et al*, 2010), EEF2K (Meloche & Roux, 2012), CDC25A (Busino *et al*, 2003) and CTNNB1 (Hart *et al*, 1999) (Fig5D). As expected, we also identified FOXN2 as a βTRCP2-binding protein (Fig5D). We validated that FOXN2 binds to βTRCP and βTRCP2, but not to βTRCP substrate binding-defective mutant R474A (Inuzuka *et al*, 2010) (Fig5E). βTRCP and βTRCP2, but not the mutant βTRCP (R474A), promoted FOXN2 ubiquitination *in vivo* (Fig5F). While knocking down CUL1 significantly stabilized FOXN2, knocking down βTRCP or βTRCP2 only partially stabilized FOXN2 (Fig5G), which agrees with βTRCP and βTRCP2 being highly related proteins and having overlapping functions in the cell. On the basis of the data presented above, we propose that FOXN2 associates with RFX1 on chromatin to carry out its transcriptional functions, but that FOXN2 itself is regulated by proteasome-mediated degradation in the soluble fraction (Fig5H). This example indicates that by investigating the different interacting proteins in soluble and chromatin fractions, we are able to gain further insights into the regulations and functions of transcription factors.

FOXN2 HCIPs form distinct complexes in chromatin versus soluble fractions. The size of prey dots indicates the estimated abundance of preys. Lines indicate the interactions defined in the literature. CUL1 was identified in a parallel virus-based FOXN2 purification.

293T cells were transfected with constructs encoding MYC-tagged RFX1 and SFB-tagged FOXN2 or its DNA binding-defective mutant FOXN2 (H162R) as indicated. Pull-down experiments were carried out with S-protein beads and immunoblotted with antibodies as indicated.

Overlap Venn diagram of FOXN2 and RFX1 target genes identified by ChIP-sequencing. 293T cells stably expressing SFB-tagged FOXN2 or RFX1 were subjected to ChIP-sequencing using anti-FLAG antibody. Each experiment was performed with two biological replicates, and four control ChIP-sequencings were performed using 293T cells stably expressing other TFs.

Reverse purification of FBXW11 (βTRCP2)-containing protein complexes conducted using the same TAP/MS protocol recovered FOXN2 as FBXW11-binding protein. Prey names, peptide counts and whether or not the interactions have been reported were listed.

293T cells were transfected with constructs encoding SFB-tagged FOXN2 and MYC-tagged βTRCP, its substrate binding-defective mutant βTRCP (R474A), or βTRCP2 as indicated. Pull-down experiments were carried out with S-protein beads and immunoblotted with antibodies as indicated.

*In vivo* ubiquitination assays were performed by co-transfecting constructs encoding FLAG-tagged FOXN2, His-tagged ubiquitin, MYC-tagged βTRCP, βTRCP (R474A) or βTRCP2 into HEK293T cells as indicated. Cell lysates were denatured with 1% SDS and diluted 10-fold using PBS prior to the pull-down by Ni-NTA resin, followed by immunoblot with antibodies as indicated.

293T or 293T-shβTRCP2, 293T-shβTRCP2, 293T-shCUL1 cells were treated with 100 mM cycloheximide (CHX) for the indicated time. Immunoblotting was conducted with antibodies as indicated.

A model showing on/off chromatin regulation of FOXN2 by transcriptional co-factors or E3 ligase complexes. All of the components indicated were identified from FOXN2 purifications. Source data are available online for this figure.

**Figure 6:**
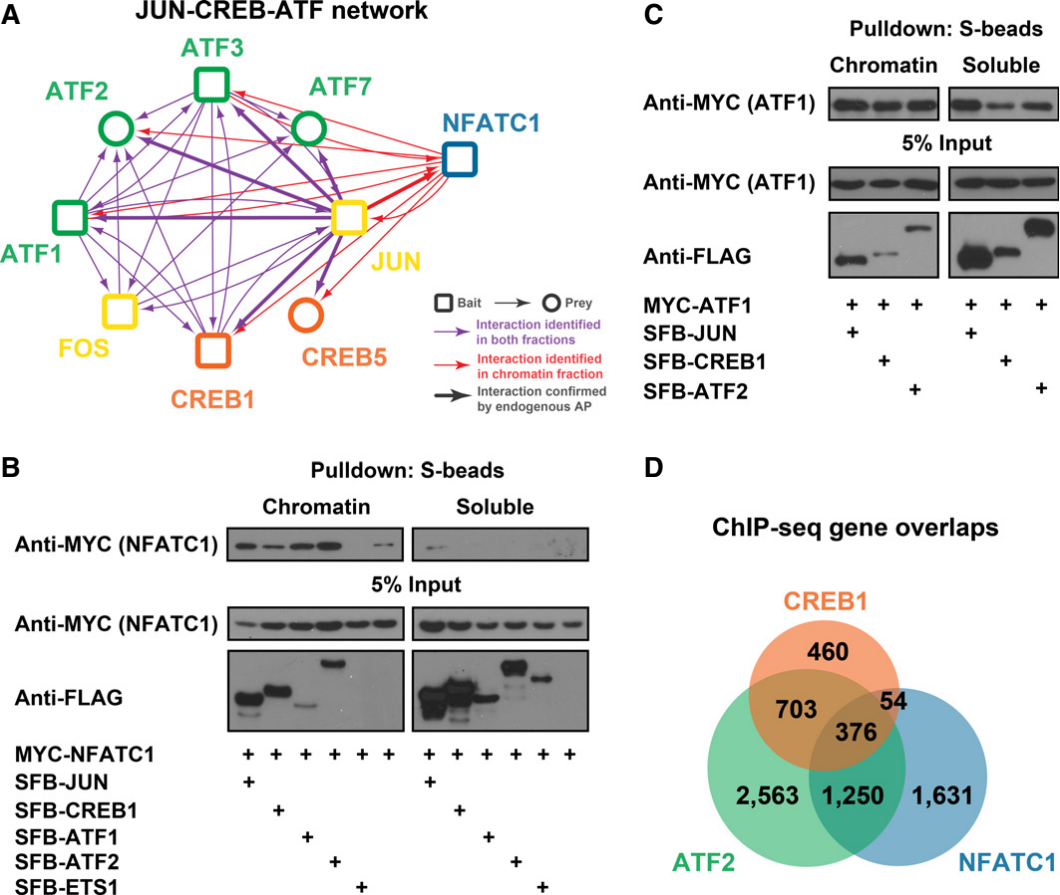
Overview of JUN/CREB/ATF/NFATC1 subnetwork

To further confirm that our newly established TF fraction-specific PPI network can be used for predicting novel protein functions or regulations, we expanded our studies on the JUN/CREB/ATF/NFATC subnetwork. Our TAP/MS and corresponding reciprocal TAP/MS analysis showed that CREB1 and NFATC1/2 bind to each other only in the chromatin fraction, which is also the case for ATF1/2/3/7 and NFATC1 (Table2, Supplementary Table S4). However, CREB1 and ATFs always bind to each other, regardless of whether they are on or off chromatin (Table2, Supplementary Table S4). To confirm that these fraction-specific complex formations truly reflect the endogenous situation, we further conducted endogenous JUN purifications in chromatin and soluble fractions (Supplementary Table S9). 65% high-confident interactions identified by our tagged TAP/MS have been confirmed by these AP/MS using antibodies against endogenous JUN. More importantly, we found that the fraction specificities are highly reproducible with endogenous AP. FOS, ATF2/3/7 and CREB5 were found in both fractions, while NFATC1 still only appeared in chromatin fractions (Supplementary Table S9). Putting these TAP/MS data together, we built a JUN/CREB/ATF network (Fig6A). Western analysis confirmed that NFATC1 binds to JUN, CREB1 and ATF1/2 predominantly in the chromatin fraction (Fig6B), while ATF1 binds to JUN/CREB/ATF2 in both fractions (Fig6C). ChIP-sequencing results from the ENCODE database suggested that 68% of CREB1 target genes overlapped with ATF2 target genes, while only 27% overlapped with NFATC1. 87% of the CREB1/NFATC1 co-target genes are also targeted by ATF2 (Fig6D). These data indicate that JUN/CREBs/ATFs are likely to form a stable complex, while they may only associate with NFATC when they are targeted to chromatin and act with NFATC to control gene transcription.

JUN/CREB/ATF/NFATC1 subnetwork map. Arrows indicate the identifications from TAP/MS. Bold arrows indicate the identifications from both TAP/MS and endogenous AP. Colours of arrows indicate the locations of interactions: red, in chromatin only; purple, in both fractions.

NFATC1 binds to other factors mainly in chromatin fractions. 293T cells were transfected with constructs encoding MYC-tagged NFATC1 and SFB-tagged other TFs as indicated. Pull-down experiments were carried out with S-protein beads and immunoblotted with antibodies as indicated.

ATF1 binds to ATF2, JUN and CREB1 in both fractions. 293T cells were transfected with constructs encoding MYC-tagged ATF1 and SFB-tagged other TFs as indicated. Pull-down experiments were carried out with S-protein beads and immunoblotted with antibodies as indicated.

Overlap Venn diagram of CREB1, ATF2 and NFATC1 target genes using ChIP-seq data sets generated by the ENCODE consortium in GM12878 cells. Source data are available online for this figure.

To further characterize these TFs and their associated proteins in the context of biological processes, we carried out pathway analysis to identify the new biological insights of baits indicated by their HCIPs (Supplementary Table S10) and the analysis of alteration of HCIPs of each bait in multiple cancer databases (Supplementary Table S11). These analyses link the TFs to a wide variety of cellular functions and disease correlations. For instance, we compared the GO annotation results of RBPJ (Supplementary Fig S4A) and FOXO3 (Supplementary Fig S4B) based on their non-self-interact HCIPs (TF-HCIP) or with the known functions in the literature (TF literature). We have not only identified previously reported functions such as Notch signalling (Tanigaki & Honjo, 2010) and PI3K/AKT signalling (Brunet *et al*, 1999; Tzivion *et al*, 2011), oestrogen receptor signalling (Guo & Sonenshein, 2004; Xia *et al*, 2006), but also novel function or regulations, such as RBPJ might be regulated by DNA methylation-mediated transcriptional repression (Supplementary Fig S4A), and FOXO3 is potentially involved in checkpoint signalling in the DNA damage response (Supplementary Fig S4B).

## Discussion

In this study, we revealed chromatin-associated and soluble complexomes for each of the 56 TFs, which validated our hypothesis that TFs form unique protein complexes on and off chromatin. These results and other information presented in this study offer new insights into the regulation of TFs and their diverse *in vivo* functions.

Among our 214 TAP/MS results, there are several results that have few or no prey identifications. We believe this is due to several reasons. In many cases, such as in the cases of FOXC2 and FOXJ2, the bait proteins expressed well and could be found in both fractions. For example, we identified 38 and 64 peptides of FOXC2, 129 and 100 peptides of FOXJ2 in chromatin and soluble fractions, respectively. After the removal of non-specific interacting proteins such as chaperones, there is no HCIP left in the soluble fraction lists, which may indicate that these proteins only form functional complexes on chromatin. In some other cases, such as FOXA2 and ETS1, the bait proteins are highly enriched in one fraction. For example, we identified 20 peptides of FOXA2 in the chromatin fraction, but none in the soluble fraction. Similarly, we identified 59 peptides of ETS1 in the chromatin fraction and 402 peptides in the soluble fraction. In these cases when the bait protein was predominantly presented in one fraction, the lack of HCIPs in the other fraction could reflect the nature of these bait TFs, which predominantly form functional complexes in one fraction. Of course, this could also be due to technical reasons, especially for chromatin fractions, since we may lose some of the associated proteins during extraction of chromatin-associated proteins. In the case of FOXO4, the failure to identify HCIPs could be just due to technical issues, since we only recovered a few peptides of FOXO4 in either fractions, which may indicate problems with protein expression or stability. It is known that AP/MS covers only a limited portion of the total peptide population (Liu *et al*, 2004), and some proteins are relatively difficult to recognize by MS because of their abundance or sequence/structural features (Altelaar *et al*, 2013); the peptide readings per se may not reflect the real biological importance of these protein–protein interactions, especially if these interactions are regulated in a signalling pathway. For instance, we repeated our NF-κB1 purifications and were able to obtain RELB (Supplementary Table S12), a known NF-κB1-binding protein (Bouwmeester *et al*, 2004). Thus, more replicates may help to uncover additional HCIPs, but at a higher cost.

Affinity purification (AP) is commonly used in large-scale proteomics studies in mammalian systems, which led to several milestone discoveries. However, the method has its limitations, especially in detecting transient or regulated interacting proteins in the presence of highly abundant, non-specific associated proteins (Figeys *et al*, 2001; McHugh & Arthur, 2008). The major challenge to identify the relatively weak but regulated interactions is to eliminate the huge amount of common contaminants and abundant proteins frequently shown in the MS, since the real signals are often buried in the large amount of unspecific noise. These contaminants mainly come from two difference sources. One is from the pull-down assay per se, which usually generates a list of non-specific binding proteins that have affinity for the particular matrix (i.e. antibodies or other) one uses. This could be easily removed during our tandem affinity purification, since the non-specific binding proteins are unlikely to have affinity towards two different affinity matrixes. The other source comes from the binding of overexpressed bait with in most cases abundant cellular proteins. For example, many tagged proteins would associate with various heat shock proteins, tubulins and RNA-binding proteins. In most cases, these commonly identified associated proteins that show up in multiple purifications are eliminated by our bioinformatics analysis using the modified SAINT method.

In this study, we employed a modified TAP method for isolating protein complexes and obtained results that could be further validated. Thus, we hope our TAP/MS-bioinformatics package could provide an easy and accurate way of studying protein–protein interactions, which can be expanded not only to protein families and signalling pathways, but also to the entire ORFeome in the future. Of course, this approach has its own drawbacks, including the use of tags that may interfere with certain PPIs, overexpression of a given protein (which may lead to its mislocalization, misfolding or both) and very weak binding proteins that may be lost during the two-step purifications. In addition, our stringent criteria for selecting HCIPs could filter out some true but weakly or transiently interacting proteins. We may need to develop a new computational method specifically designed for this TAP/MS approach to obtain as many true interacting proteins as possible, but at the same time eliminate contaminants and abundant proteins that are often associated with the baits.

Several previous reports have suggested that the association between certain TFs and chromatin is tightly regulated and that these TFs could shuttle on and off chromatin. However, whether this applies to other TFs remains largely unknown. In other words, are there often “free” transcription factors that exist off chromatin? Based on the data presented above, the answer is yes. Indeed, the distribution of TFs and their associated proteins in these fractions differs dramatically (Supplementary Fig S1D; also see Fig3). Most TFs form very different protein complexes on and off chromatin, which means that soluble and chromatin-bound fractions exist for many TFs and the majority of the proteins in soluble fractions are not just the proteins released from chromatin during purification. The separation of soluble nuclear proteins versus soluble cytosolic proteins is irrelevant to our study, since these soluble TF-associated proteins should not be directly involved in transcriptional regulation, regardless of where they are (i.e. in the nuclei or in the cytosol).

Are these “location-specific” interactomes specific and relevant to the regulation of protein functions? We believe the answer is also a yes. The protein complexes in different fractions are highly specific and functionally relevant. In 14 reciprocal TAP/MS analyses that successfully identified bait proteins, 13 of which were uncovered in the corresponding fractions (Fig2F and G). The reciprocal TAP/MS of βTRCP2/FBXW11, which was identified from FOXN2 soluble fractions, also captured FOXN2 only in the soluble fractions (Fig5C). This means that complexes are not just randomly distributed in chromatin and soluble fractions, but they are highly organized and regulated. Another interesting example is that JUN/FOS, CREB1/5 and ATF1/2/3/7 are likely to form a stable transcription complex, while they may only associate with NFATC when they are targeted to chromatin to regulate gene transcription. This hypothesis is, at least partially, supported by reports indicating that CREB1 is targeted to the same promoter sequence as NFATC under certain stimulations (Sato *et al*, 2006; Suehiro *et al*, 2010).

On the basis of our proteomics studies, we proposed that we could use this location-specific (i.e. chromatin-associated and soluble) interactomes to predict how TFs function or are regulated in the cell (Figs4–6 and Supplementary Fig S4). For example, we found that FOXM1 formed two distinct complexes on and off chromatin, but both complexes are potentially involved in the same biological events: the regulation of mitosis and the promotion of cell cycle progression (Fig4D–F), while the two FOXN2 complexes formed on and off chromatin had opposing roles in regulating FOXN2 functions (Fig5). In addition, the functional annotation based on the HCIPs correlates well with some of the known functions in the literature. For example, RBPJ/CBF1, a TF that plays a central role in Notch signalling (Tanigaki & Honjo, 2010), is functionally annotated to be linked with the Notch signalling pathway (Supplementary Fig S4A). Similarly, on the basis of our analysis, the well-studied FOXO3 acts in PI3K/AKT signalling (Brunet *et al*, 1999; Tzivion *et al*, 2011), oestrogen receptor signalling (Guo & Sonenshein, 2004; Xia *et al*, 2006), ERK5 signalling (Finegan *et al*, 2009) and cell cycle/checkpoint regulation (Chung *et al*, 2012). These results agree well with the known diverse functions of FOXO3 in these processes (Supplementary Fig S4B). Moreover, both RBPJ and FOXO3 proteins could be functionally annotated to novel function or regulations based on our PPI studies: RBPJ might be regulated by DNA methylation-mediated transcriptional repression (Supplementary Fig S4A), and FOXO3 is potentially involved in BRCA1 signalling in DNA damage response (Supplementary Fig S4B). Therefore, we anticipate that these functional annotations defined by guilty by association will be beneficial, especially when studying proteins with unknown functions. However, since proteins often form distinct protein complexes in different environments or tissues to execute their tissue-specific functions, one needs to be cautious when drawing any conclusion solely based on PPI studies. In these cases, the functional validation and relevance should be the most important aspects and these leads should be pursued with a biological question in mind. Thus, our functional prediction and disease correlations based on the TAP/MS results performed in HEK293T cells only represent a fraction of the functions carried out by these bait proteins and should only be used as references.

In summary, our study offers a valuable resource of protein–protein interaction networks for transcription factors involved in many signalling pathways and human diseases. Although it may not come as a total surprise, our findings highlight that transcription factors form distinct complexes on and off chromatin. This location-based interactomes may be used to predict the molecular mechanisms underlying the regulations of these transcription factors and their associated biological functions.

## Materials and Methods

### Constructs and small hairpin RNAs (shRNAs)

FOXK1 and FOXK2 cDNAs were a generous gift from Dr. Andrew D. Sharrocks (Ji *et al*, 2012). βTRCP (R474A) cDNA was a generous gift from Dr. Wenyi Wei (Inuzuka *et al*, 2010). cDNAs encoding other known FOX proteins and TP53, MYC, MAX, RBPJ, TCF4, TEAD, JUN, FOS, NF-κB, SMAD4, ATF6, CREB1, ETS1, GLI1, IRF3, MEF2A, NFATC1, PPARG, STAT3, L3MBTL2, L3MBTL3, E2F6, HOXD13, ATF1, ATF2, ATF3, ATF7, HMGA1, ZNF131, KDM1, FBXO42, NFIX, CBX3, EPC2, MTERF, XRCC1, TFAP2A, VRK3, MECP2, TFDP1, EEF1D, LIG3, RPA2, H2AFY, RECQL, PARS2, DDX52, QARS, C1orf25, DARS, ORC2L, PES1, IARS2, SMARCA1, SMARCA5, LARS, LRWD1, ZC3H11A, CDC27, RB1, RBL1, PNKP, TAF5L, ARHGEF2, RFX1, βTRCP and βTRCP2 were obtained from the hORFV5.1 library and Open Biosystems. cDNAs were subcloned into the pDONR201 vector (Invitrogen) as entry clones and subsequently transferred to gateway-compatible destination vectors for the expression of C-terminal SFB-, MYC-, GFP-tagged fusion proteins. Point or deletion mutants were generated using sequential PCR methods and verified by sequencing. FOXN2 DNA binding-defective mutant H162R was generated based on the conserved DNA-binding domain reported for FOXO3 (Harada *et al*, 2010).

Four individual pGIPZ lentiviral shRNAs targeting βTRCP, βTRCP2, CUL1, ARHGEF2, TRRAP, TAF5L, CDC27, RB1, RBL1, PNKP, FOXK1, EP300, CREBBP, MECP2, LIG3, TFAP2A, TBP, FOXK2, L3MBTL2, SMARCA1, RPA2, EEF1D, WIZ, TFDP1, TWIST1, SAP130, H2AFY, XRCC1, RECQL, VRK3, MTERF, MORC2, BAZ1A, MXI1 and MXD4 were obtained from the shRNA and ORFeome core facility at The University of Texas MD Anderson Cancer Center. All lentiviral supernatants were generated by transient transfection of 293T cells with packaging plasmids pSPAX2 and pMD2G and harvested 48 h later. Supernatants were passed through a 0.45-μm filter and used to infect HEK293T and MCF10A cells with the addition of 8 μg/ml polybrene.

### Cell culture, treatments and transfection

HEK293T cells were cultured in Dulbecco's modified Eagle's medium supplemented with 10% foetal bovine serum and 1% penicillin and streptomycin. MCF10A cells were cultured in Dulbecco's modified Eagle's medium/F12 supplemented with 5% horse serum, 10 μg/ml insulin, 20 μg/ml epidermal growth factor, 0.5 μg/ml hydrocortisone, 0.1 μg/ml cholera toxin and 1% penicillin and streptomycin.

The activity of several TFs, such as SMAD4 and NF-κB1, can be greatly influenced by specific signalling events. For this reason, we also treated some stable cells with drugs to promote the nuclear translocation of some TFs. We performed purifications of SMAD4 with treatment of 5 ng/ml TGFβ or BMP4 for 16 h, TCF4 with treatment of 10 mM LiCl for 16 h or NF-κB1, JUN, FOS with treatment of 25 ng/ml TNF-α for 8 h to promote chromatin association of the corresponding TFs. In many cases, we were able to isolate chromatin-associated proteins of these TFs even without any treatment.

Constructs encoding C-terminally SFB-tagged TFs were transfected into HEK293T cells using polyethylenimines as previously described (Wang *et al*, 2013). Cells were selected with puromycin and 12–24 single clones were picked, examined by Western blotting and immunostaining. We chose the ones with the correct subcellular localizations and the lowest expression for the subsequent TAP/MS analysis.

### TAP of TF-associated protein complexes and MS analysis

A total of 1 × 10^8^ HEK293T cells stably expressing tagged TFs were lysed with NETN buffer [20 mM Tris–HCl (pH 8.0), 100 mM NaCl, 1 mM EDTA and 0.5% Nonidet P-40, containing 1 μg/ml each of pepstatin A and aprotinin] for 30 min. Crude lysates were saved as the soluble fraction by centrifugation at 16,000 *g* at 4°C for 30 min, and the pellet was digested with TurboNuclease (Accelagen) for 10 min in digesting buffer [50 mM Tris (pH 8), 1 mM MgCl_2_ and protease inhibitor] to extract chromatin-bound proteins. The supernatants were cleared at 16,000 *g* to remove debris from chromatin-bound protein fractions. Both fractions were then incubated with streptavidin-conjugated beads (Amersham) for 2 h at 4°C. The beads were washed three times with NETN buffer, and the bead-bound proteins were eluted with NETN buffer containing 2 mg/ml biotin (Sigma). The elutes were incubated with S-protein beads (Novagen). The beads were again washed three times with NETN buffer and subjected to SDS–PAGE. Protein band containing the entire sample was excised, and MS analyses were performed by the TAPLIN Biological Mass Spectrometry Facility of Harvard University.

For MS analysis, excised gel bands were cut into approximately 1-mm^3^ pieces. Gel pieces were then subjected to in-gel trypsin digestion and dried. Samples were reconstituted in 5 μl of HPLC solvent A (2.5% acetonitrile, 0.1% formic acid). A nano-scale reverse-phase HPLC capillary column was created by packing 5-μm C18 spherical silica beads into a fused silica capillary (100 μm inner diameter × ∽20 cm length) with a flame-drawn tip. After equilibrating the column, each sample was loaded via a Famos autosampler (LC Packings, San Francisco, CA) onto the column. A gradient was formed and peptides were eluted with increasing concentrations of solvent B (97.5% acetonitrile, 0.1% formic acid).

As peptides eluted, they were subjected to electrospray ionization and then entered into an LTQ Velos ion trap mass spectrometer (Thermo Fisher, San Jose, CA). Peptides were detected, isolated and fragmented to produce a tandem mass spectrum of specific fragment ions for each peptide. Peptide sequences (and hence protein identity) were determined by matching the acquired fragmentation pattern with protein databases by the software program, SEQUEST (ver. 28) (Thermo Fisher). Enzyme specificity was set to partially tryptic with two missed cleavages. Modifications included carboxyamidomethyl (cysteines, fixed) and oxidation (methionine, variable). Mass tolerance was set to 2.0 for precursor ions and 1.0 for fragment ions. The database searched was the Human IPI databases version 3.6. The number of entries in the database was 160,900, which includes both the target (forward) and the decoy (reversed) human sequences. Spectral matches were filtered to contain < 1% FDR at the peptide level based on the target-decoy method. Finally, only tryptic matches were reported and spectral matches were manually examined. When peptides matched to multiple proteins, the peptide was assigned so that only the most logical protein was included (Occam's razor). This same principle was used for isoforms when present in the database. The longest isoform was reported as the match. Supplementary Tables S3 and S4 contain the lists of both the peptides identified and the proteins identified during these analyses.

The mass spectrometry data from this publication, including raw files and search results, have been deposited to ProteomeXchange (www.proteomexchange.org) with identifier PXD001383 and DOI 10.6019/PXD001383.

### Data analysis and bioinformatics analysis

We downloaded protein sequences from the UniProt Consortium. The function annotations were generated through the use of Ingenuity Pathway Analysis software (www.ingenuity.com). The heatmap and clustering were generated using Multi Experiment Viewer version 4.8.1 (TM4) and Heatmap Builder (Dr. Euan Ashley, Stanford University) software.

For MS data filtration, protein results from MS sequencing were converted to NCBI gene identifiers and searched for protein length. We reorganized the data to the format compatible to the SAINT program and used two-pool analysis, which recognized control group as a separate pool. We did not remove outlier datapoints. However, during the data analysis, we temporarily removed the bait self-identification in the identification list before applying the SAINT algorithms and added them back after the data filtration. The separation of positive and negative distributions was considered for the scoring of low-count interactions or for division of spectra counts by the total spectra counts of each purification. The statistics used to assess accuracy and significance of measurements was referred to the SAINT algorithms, where *X*_*ij*_* *> 0.80 was taken as the threshold required for the data quantification.

For interactomes generated by Cytoscape, we analysed the network and created custom styles, then applied *y*Files organic layout or unweighted force-directed distributions with minor adjustments when necessary. The GO annotations and disease correlations were generated using the literature or non-self HCIPs identified in our studies, weighted by the spectra counts and searched in the Knowledge Base provided by Ingenuity pathway software (Ingenuity Systems, www.ingenuity.com), which contains findings and annotations from multiple sources including the Gene Ontology database, to estimate the significance of these correlations.

### ChIP-sequencing and genomic data analysis

ChIP-sequencing was performed in HEK293T cells with two biological replicates. Samples were sequenced using the Illumina Miseq, raw reads were mapped to human reference genome (hg19), and peaks were selected using MACS in Galaxy and annotated with PAVIS (Zhang *et al*, 2008; Langmead *et al*, 2009; Huang *et al*, 2013). Data are available in the ArrayExpress database www.ebi.ac.uk/arrayexpress (Rustici *et al*, 2013) under accession number E-MTAB-3120.

Analysis of the NFATC1-, CREB1- and ATF2-bound regions was performed using ChIP-seq data sets generated by the ENCODE consortium in GM12878 cells (ENCODE-Project-Consortium, 2012). Data sets were analysed in Galaxy and mapped to genes using PAVIS with −5000 to +1000 TSS windows.

### Western blotting and immunostaining

Whole-cell lysates were prepared by lysing cells with NETN buffer [20 mM Tris–HCl (pH 8.0), 100 mM NaCl, 1 mM EDTA and 0.5% Nonidet P-40] on ice for 30 min and then boiling in 2× Laemmli buffer. Lysates were subjected to SDS–PAGE followed by immunoblotting with antibodies against various proteins, including TP53, c-MYC (the same one used for MYC-tag WB), GFP, GAPDH, FOXN2 (Santa Cruz Biotechnology), FOXK1, FOXM1, FOXO3, JUN, MAX, MYC (for endogenous MYC blotting), NF-κB1, STAT3, TCF4, βTRCP, CUL1 (Cell Signalling), β-actin, FLAG (Sigma), RBPJ, βTRCP2 (Abcam) and histone H3 (Upstate). The rabbit polyclonal anti-FOXD3 antibody was generated by immunizing rabbits (Cocalico Biologicals) with GST-FOXD3 fusion protein and affinity purified.

For immunostaining assays, cells cultured on coverslips were washed with PBS, fixed with 3% paraformaldehyde for 20 min and permeabilized with 0.5% (v/v) Triton X-100 solution for 5 min. Coverslips were washed with PBS and immunostained with primary antibodies in 5% goat serum for 60 min. Cells were then washed and incubated with rhodamine- or FITC-conjugated secondary antibodies for 60 min, and nuclei were stained with 1 μg/ml 4′,6-diamidino-2-phenylindole (DAPI). Slides were mounted and visualized using a Nikon ECLIPSE E800 fluorescence microscope with a Nikon Plan Fluor 40× oil objective lens (numerical aperture 1.30) at room temperature. Cells were photographed using a SPOT camera (Diagnostic Instruments) and analysed using Photoshop software (Adobe).

## Supporting Information

Supplementary Table S12

Review Process File
