# Supplementary figures and images for: Proteomic analyses reveal distinct chromatin-associated and soluble transcription factor complexes
Source: Mol Syst Biol. 2015 Jan 21;11(1):775. doi: 10.15252/msb.20145504 (PMC4332150; doi:10.15252/msb.20145504)

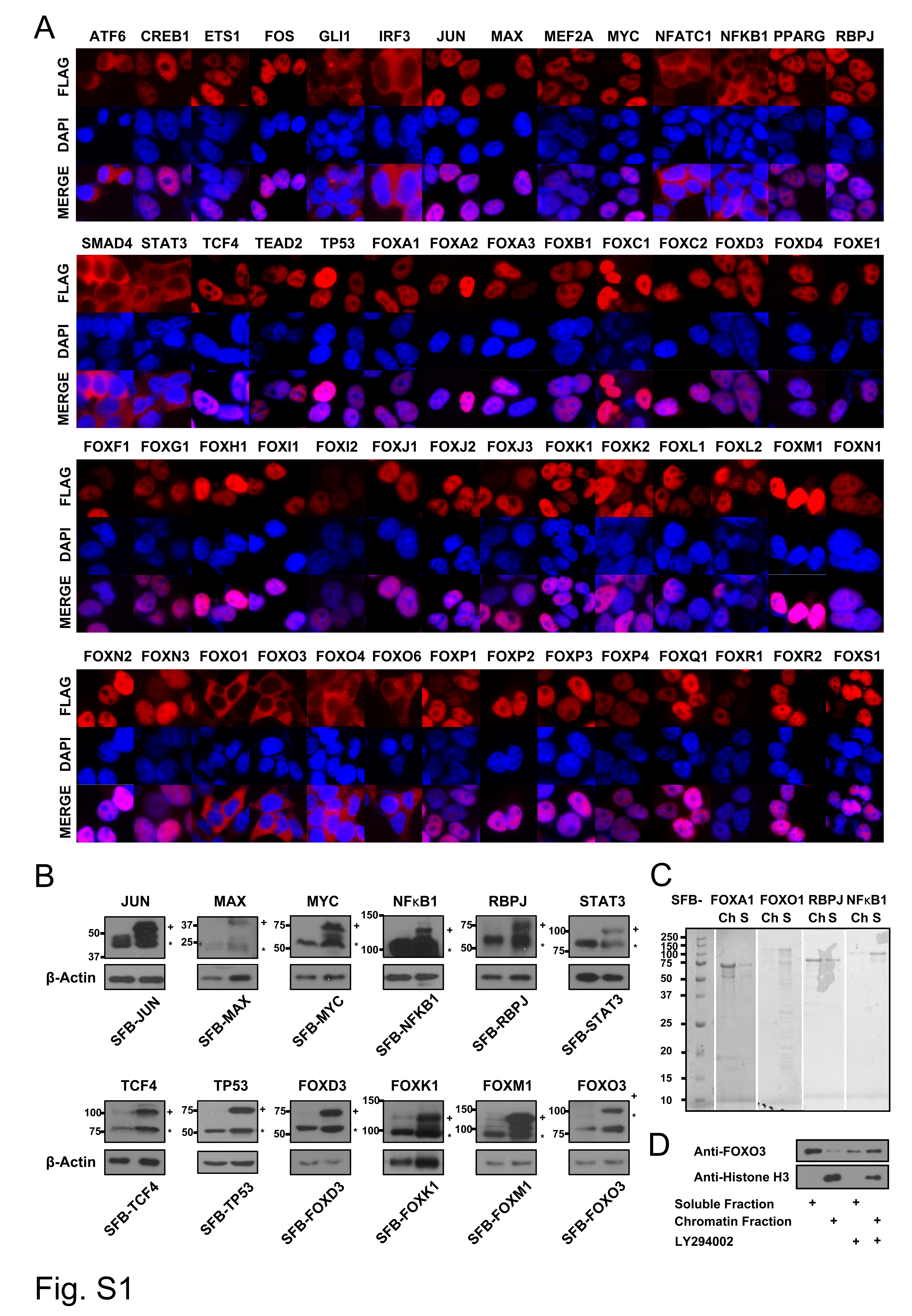

Supplement: Supplementary file 1 [file msb0011-0775-sd1.tif]

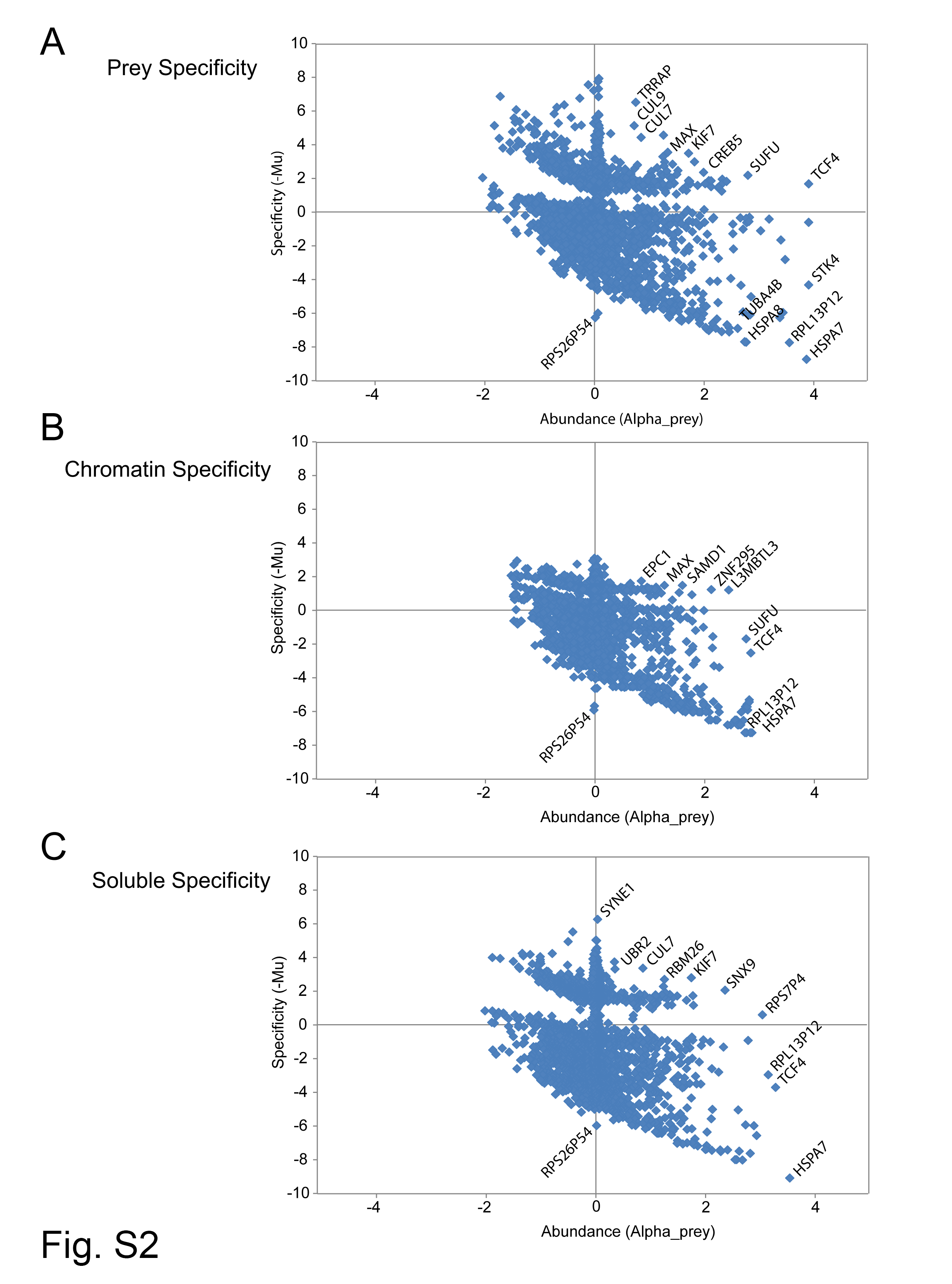

Supplement: Supplementary file 2 [file msb0011-0775-sd2.tif]

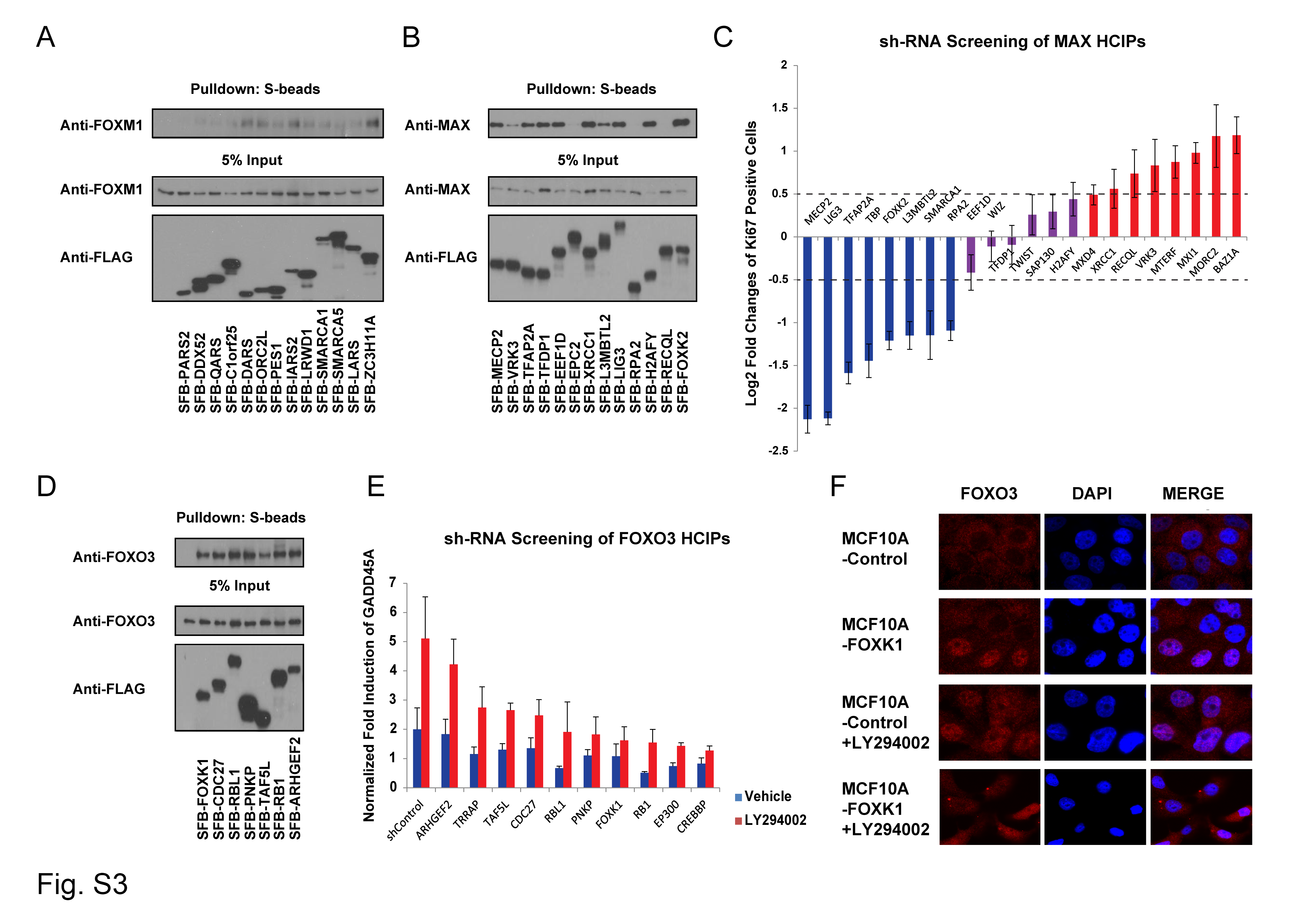

Supplement: Supplementary file 3 [file msb0011-0775-sd3.tif]

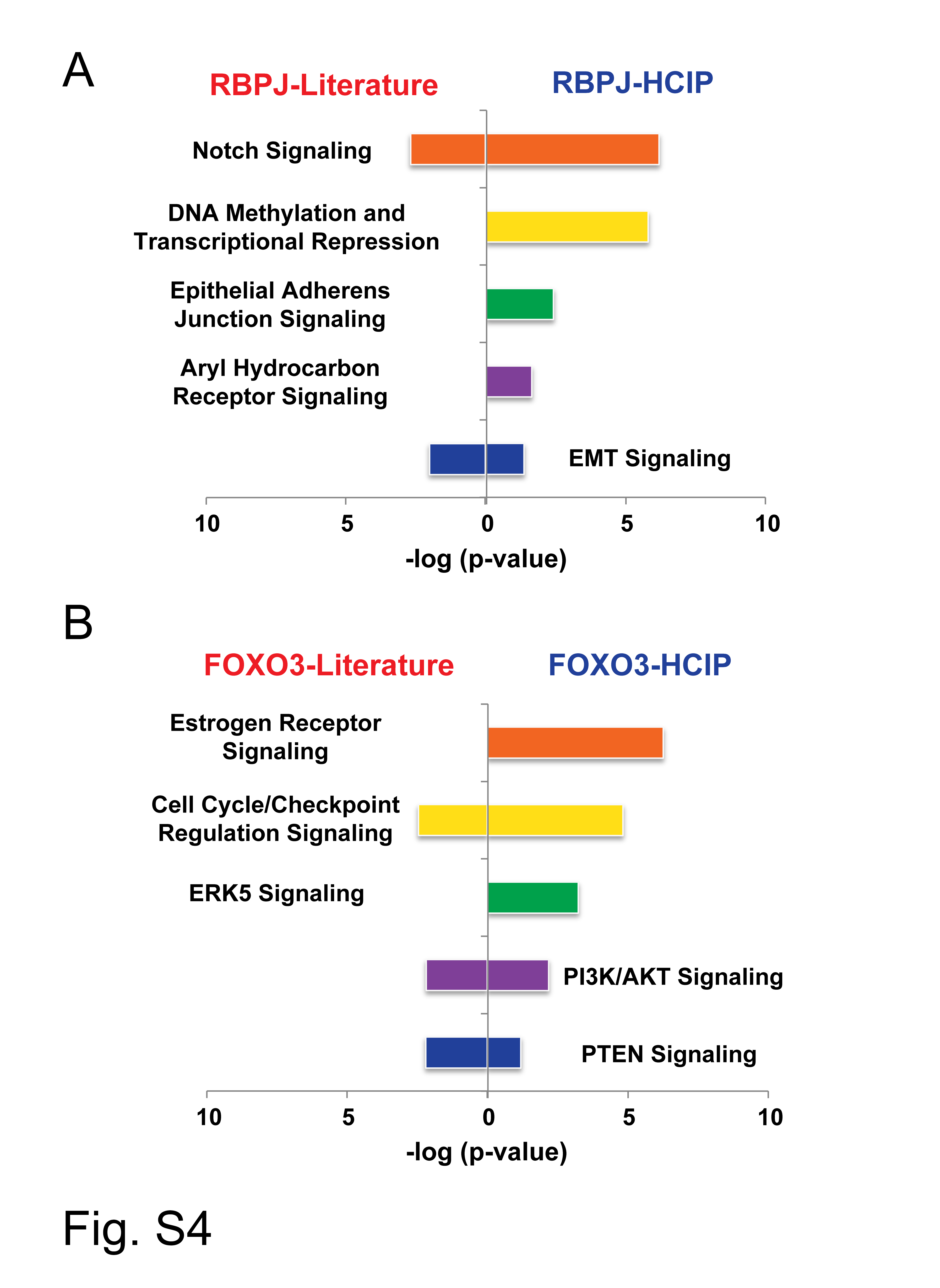

Supplement: Supplementary file 4 [file msb0011-0775-sd4.tif]

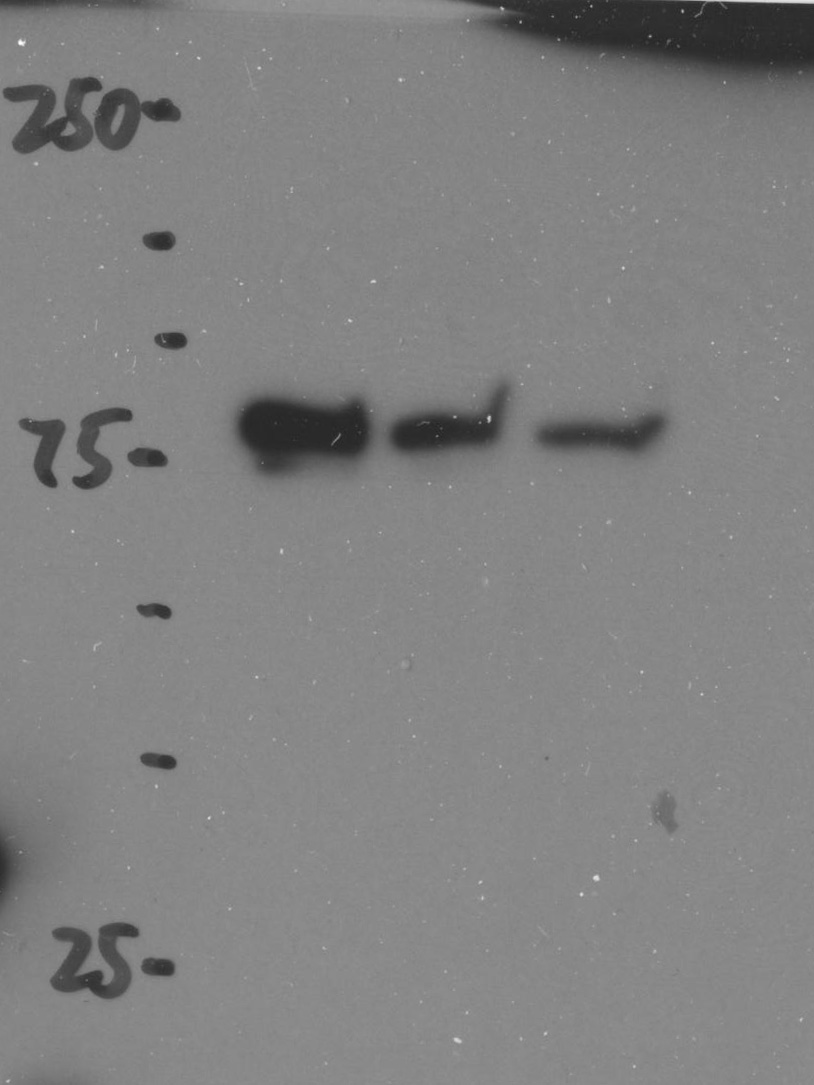

Supplement: Supplementary file 18 [file msb0011-0775-sd18.zip › Source Data for Figure 5/Fig.5B/Fig5B. Input_Anti-FLAG.jpg]

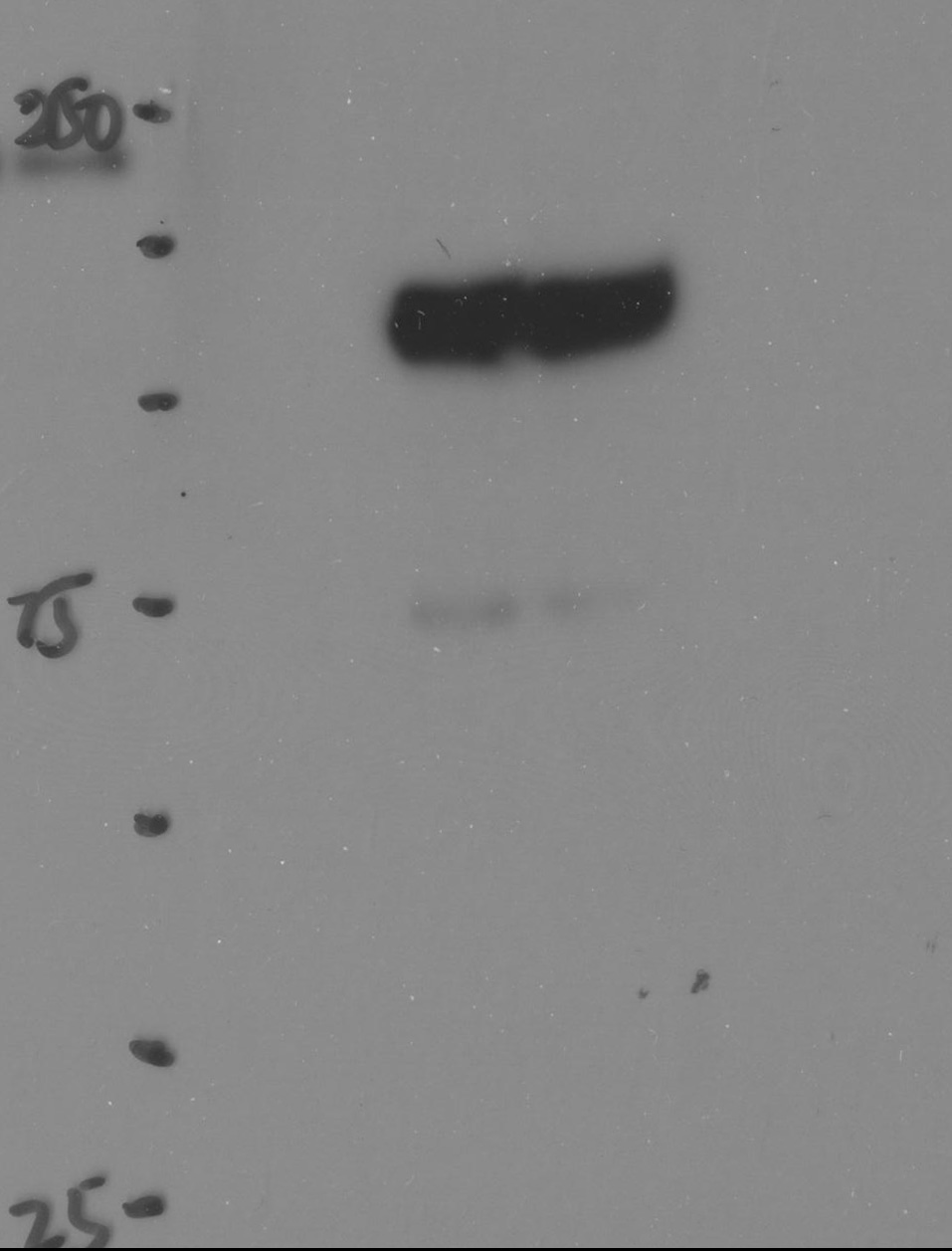

Supplement: Supplementary file 18 [file msb0011-0775-sd18.zip › Source Data for Figure 5/Fig.5B/Fig5B. Input_Anti-MYC.jpg]

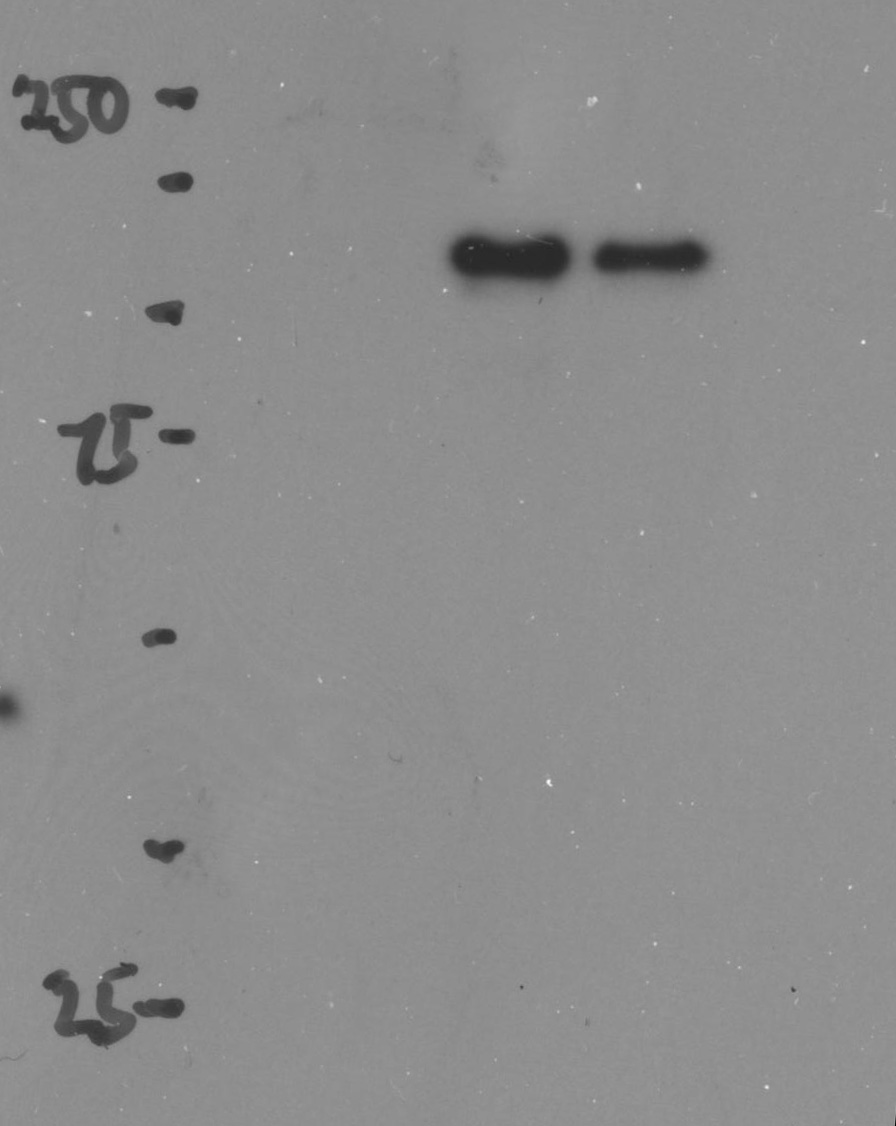

Supplement: Supplementary file 18 [file msb0011-0775-sd18.zip › Source Data for Figure 5/Fig.5B/Fig5B. Pulldown-S-beads_Anti-MYC.jpg]

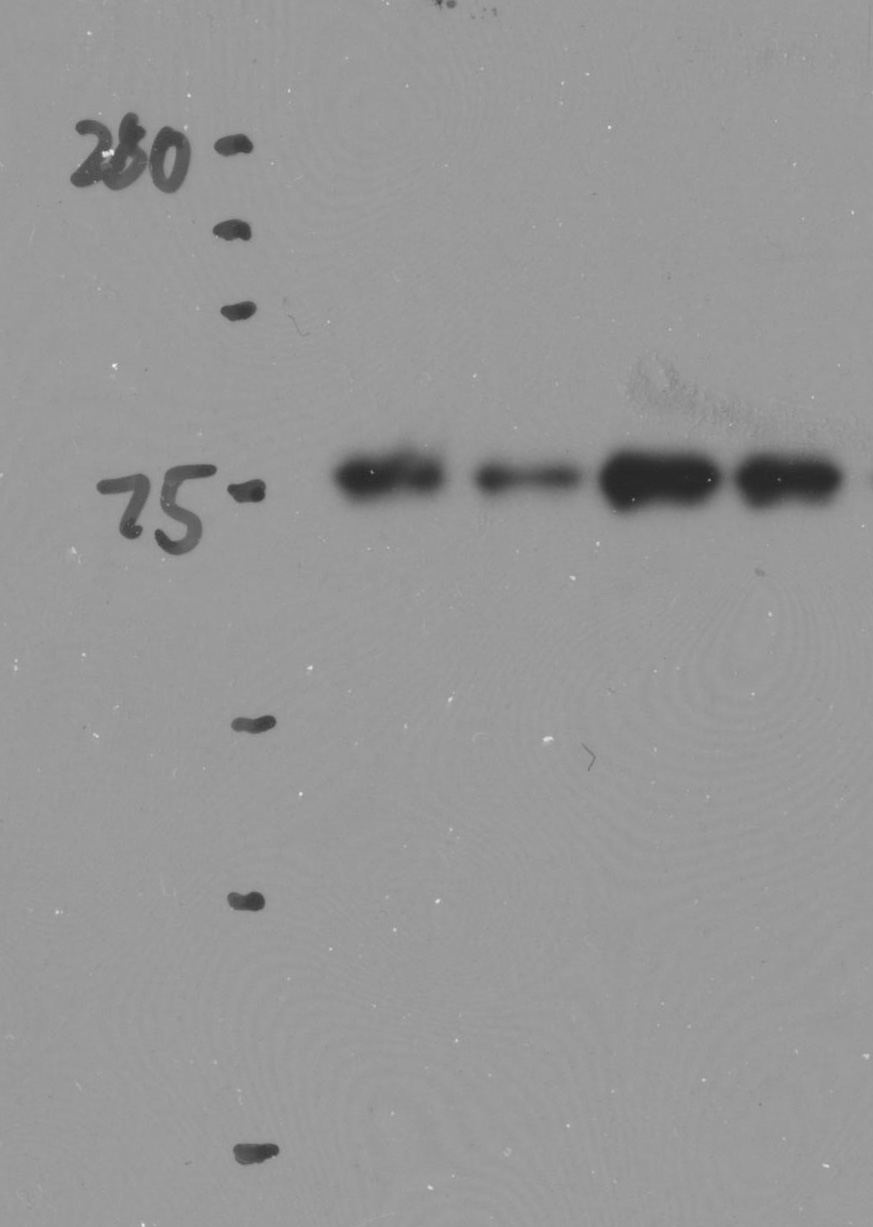

Supplement: Supplementary file 18 [file msb0011-0775-sd18.zip › Source Data for Figure 5/Fig.5E/Fig5E. Input_Anti-FLAG.jpg]

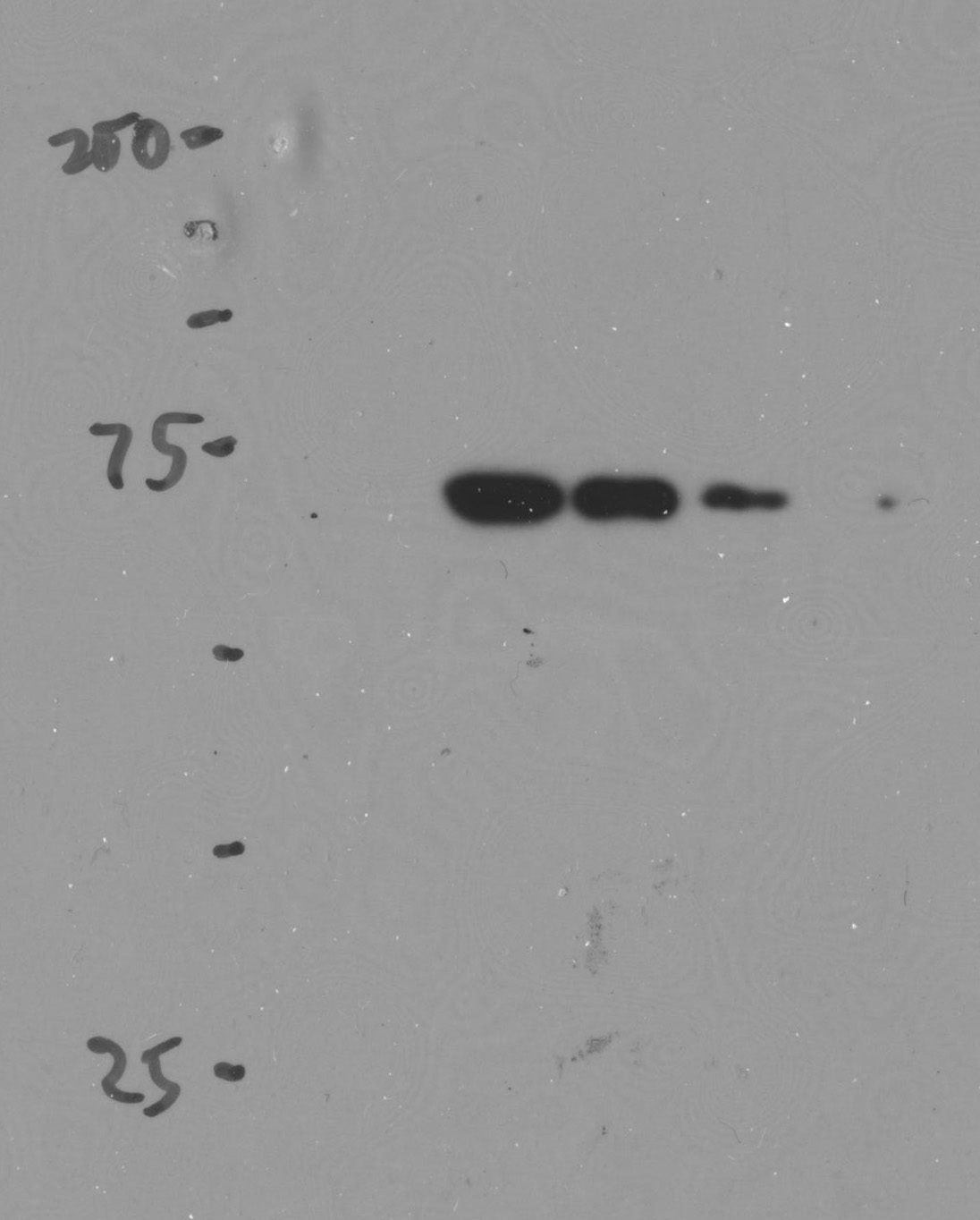

Supplement: Supplementary file 18 [file msb0011-0775-sd18.zip › Source Data for Figure 5/Fig.5E/Fig5E. Input_Anti-MYC.jpg]

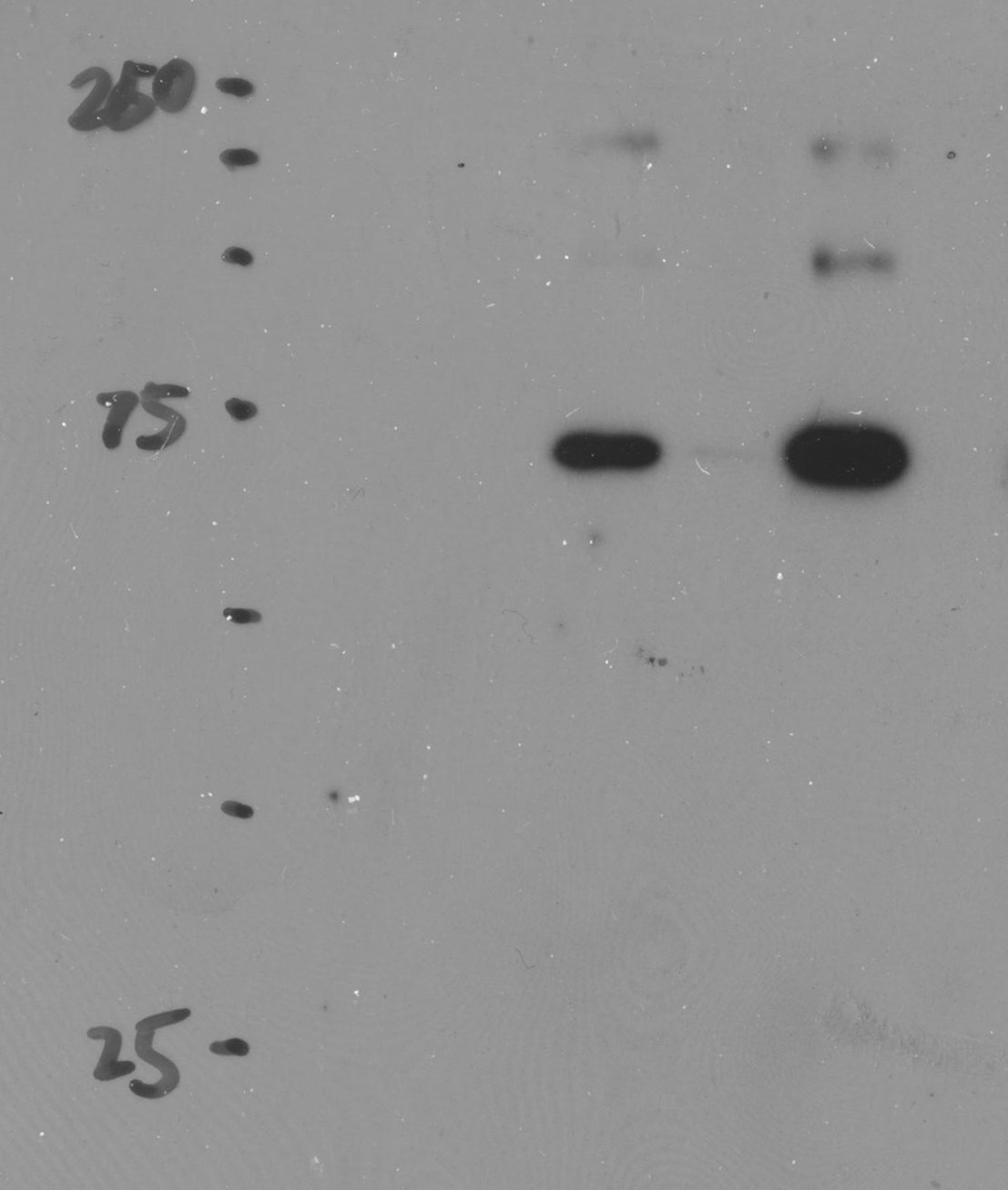

Supplement: Supplementary file 18 [file msb0011-0775-sd18.zip › Source Data for Figure 5/Fig.5E/Fig5E. Pulldown-S-beads_Anti-MYC.jpg]

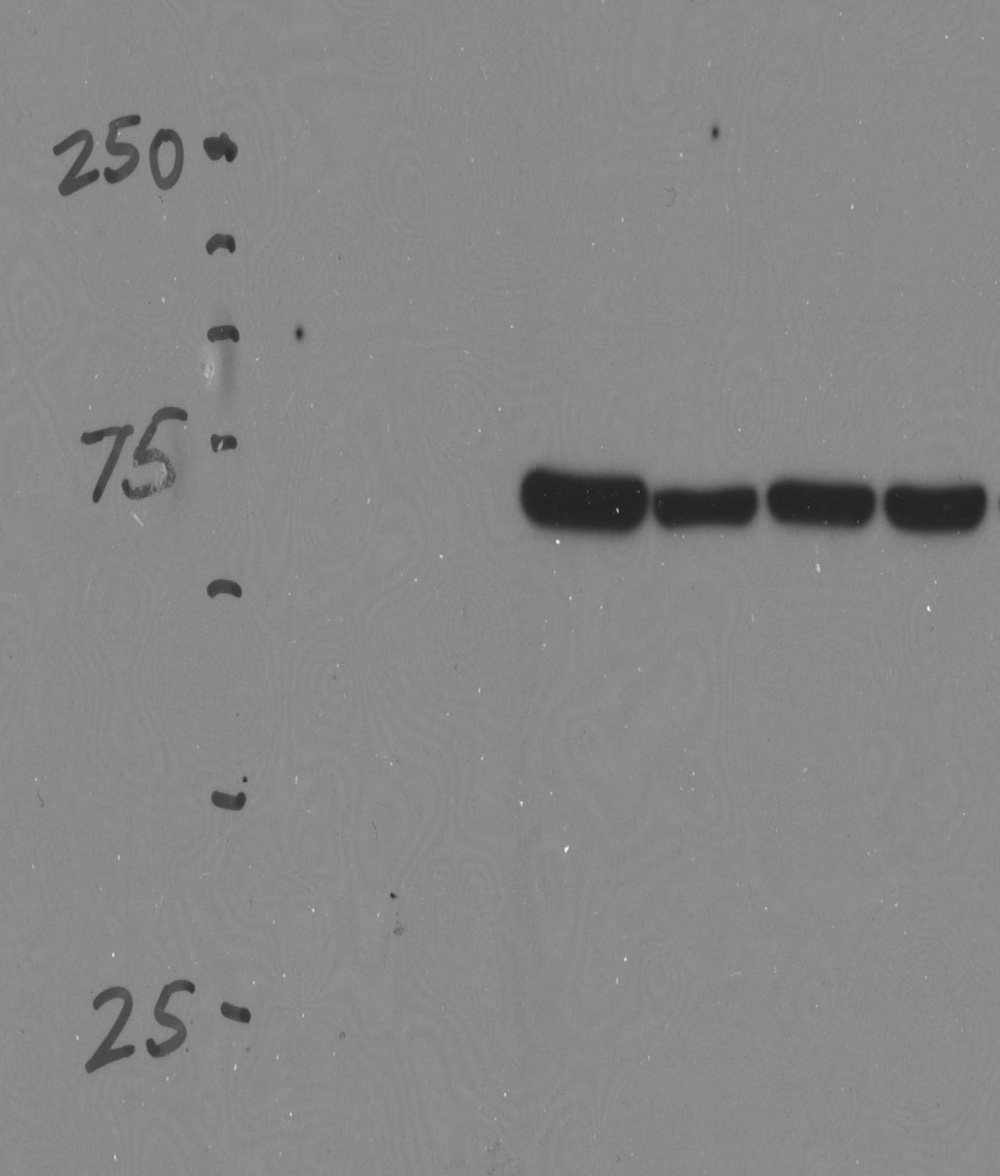

Supplement: Supplementary file 18 [file msb0011-0775-sd18.zip › Source Data for Figure 5/Fig.5F/Fig5F. Input_Anti-FLAG.jpg]

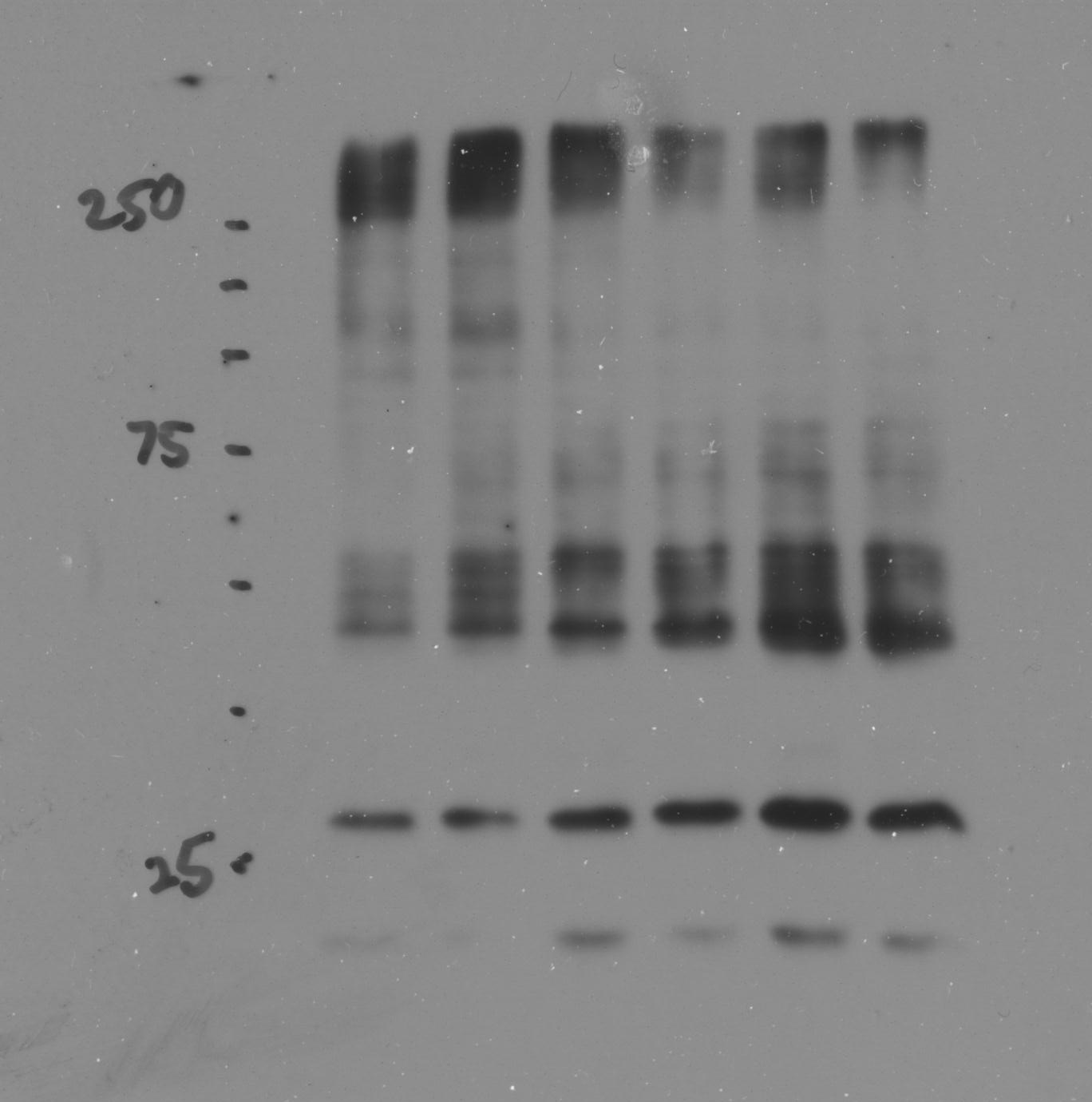

Supplement: Supplementary file 18 [file msb0011-0775-sd18.zip › Source Data for Figure 5/Fig.5F/Fig5F. Input_Anti-His.jpg]

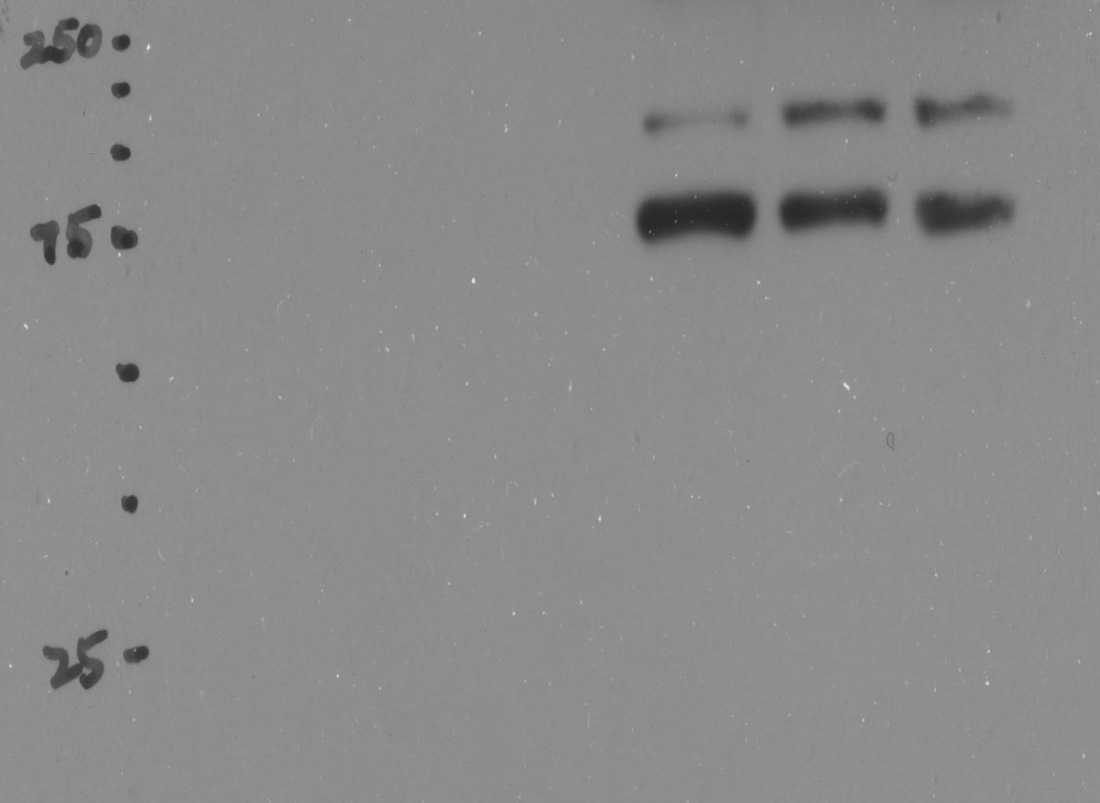

Supplement: Supplementary file 18 [file msb0011-0775-sd18.zip › Source Data for Figure 5/Fig.5F/Fig5F. Input_Anti-MYC.jpg]

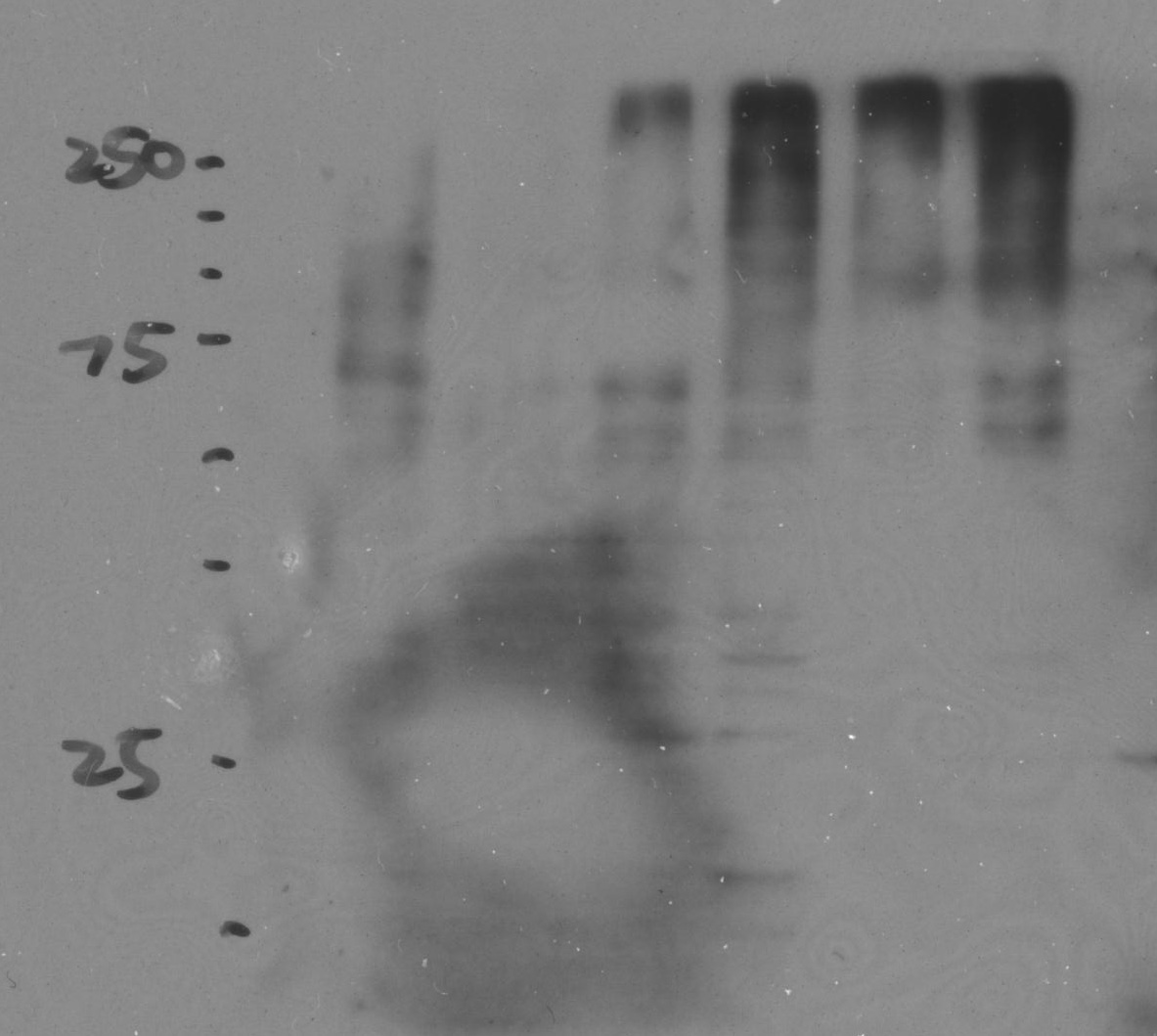

Supplement: Supplementary file 18 [file msb0011-0775-sd18.zip › Source Data for Figure 5/Fig.5F/Fig5F. Pulldown-Ni-NTA_Anti-FLAG.jpg]

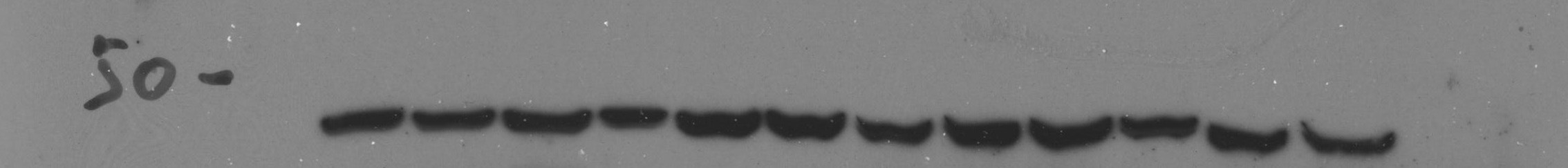

Supplement: Supplementary file 18 [file msb0011-0775-sd18.zip › Source Data for Figure 5/Fig.5G/Fig5G. 293T-shbTRCP & 293T-shbTRCP2_anti-bActin.jpg]

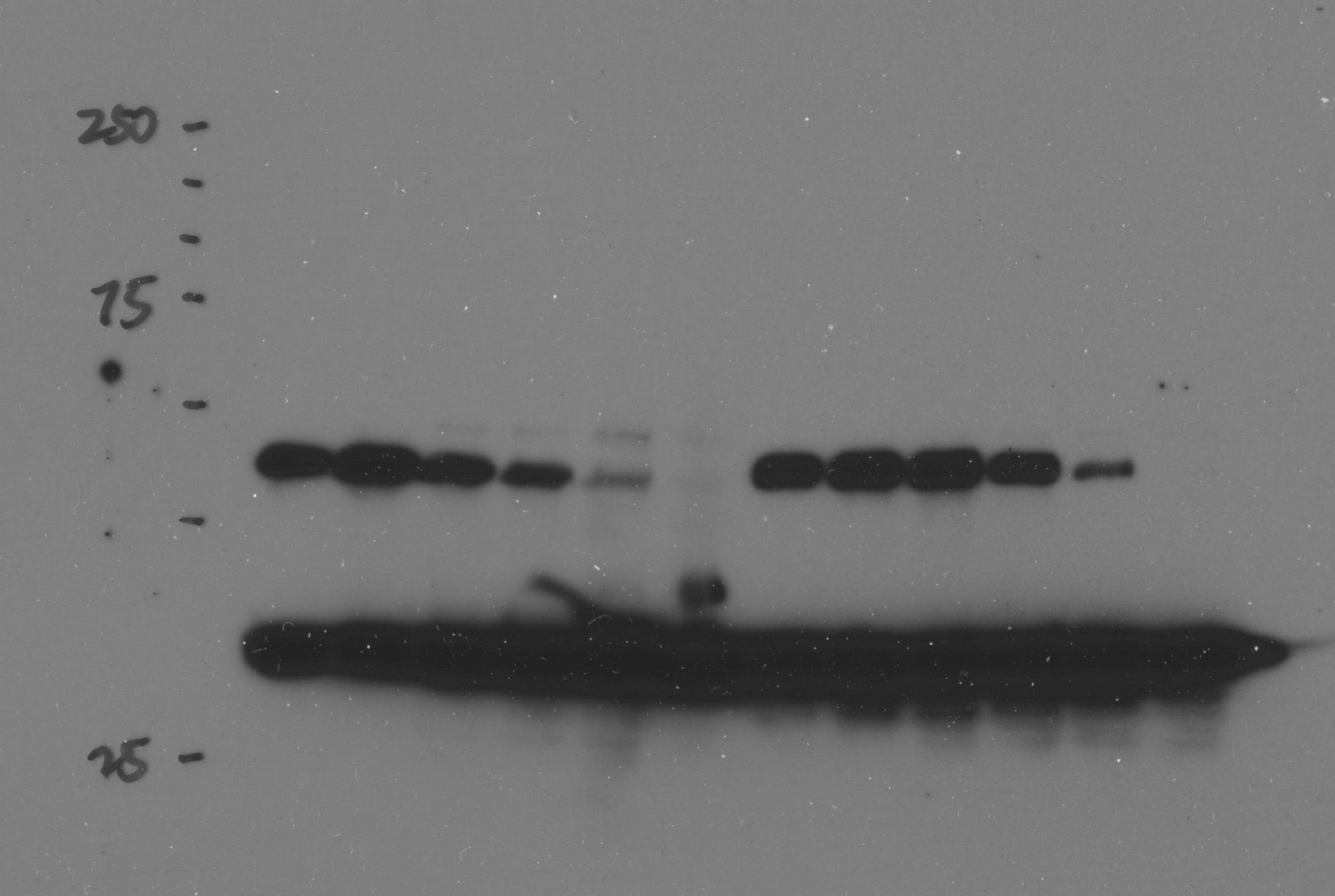

Supplement: Supplementary file 18 [file msb0011-0775-sd18.zip › Source Data for Figure 5/Fig.5G/Fig5G. 293T-shbTRCP & 293T-shbTRCP2_anti-FOXN2.jpg]

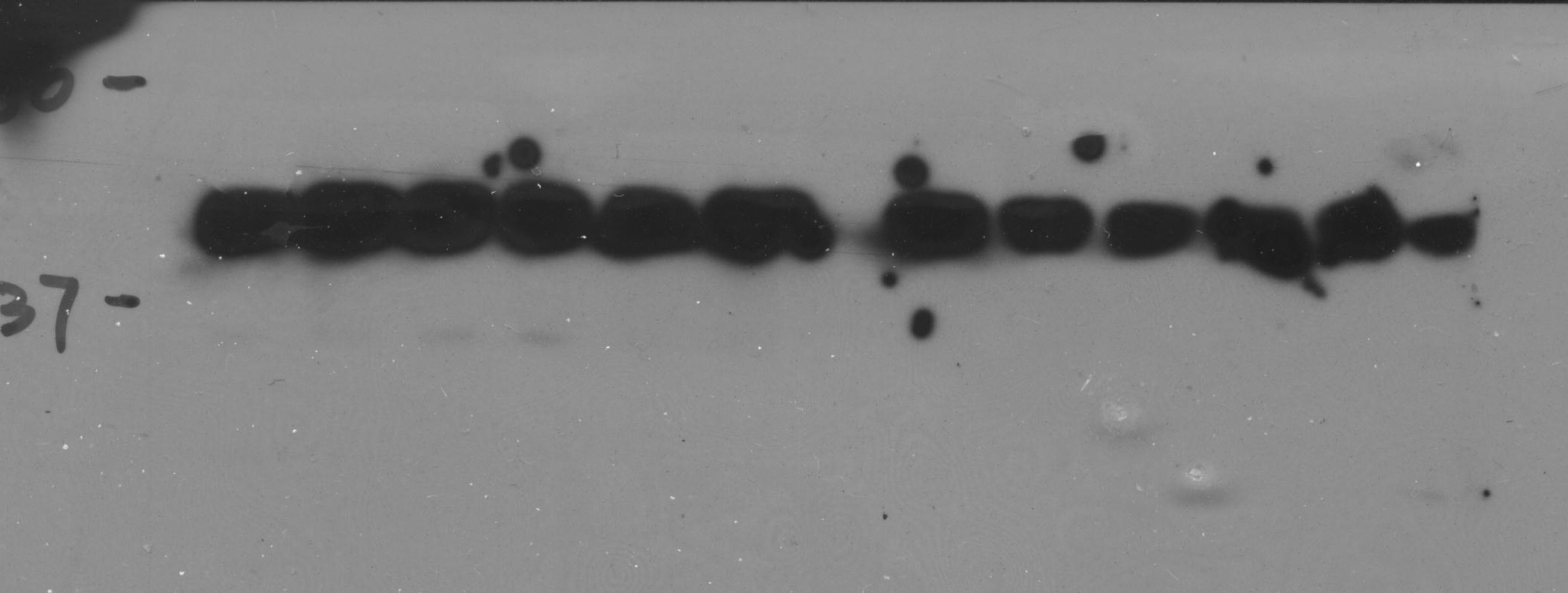

Supplement: Supplementary file 18 [file msb0011-0775-sd18.zip › Source Data for Figure 5/Fig.5G/Fig5G. 293T-shbTRCP & 293T-shbTRCP2_right panel_anti-bActin.jpg]

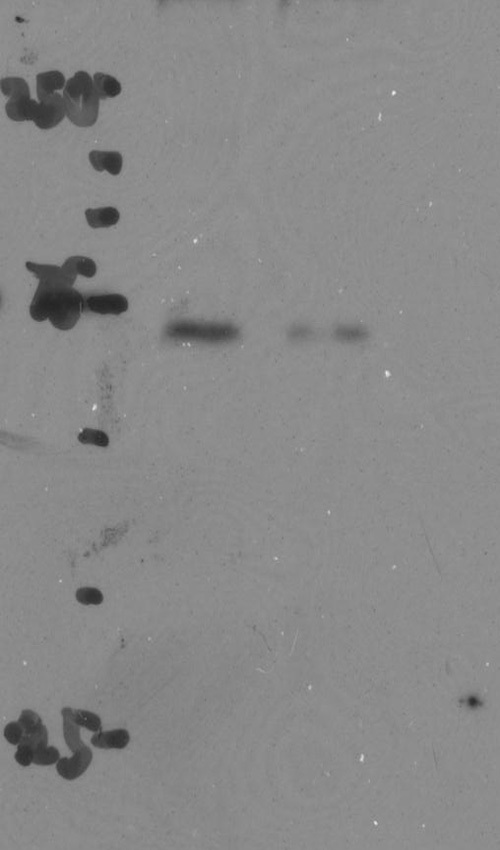

Supplement: Supplementary file 18 [file msb0011-0775-sd18.zip › Source Data for Figure 5/Fig.5G/Fig5G. 293T-shbTRCP2_anti-bTRCP2.jpg]

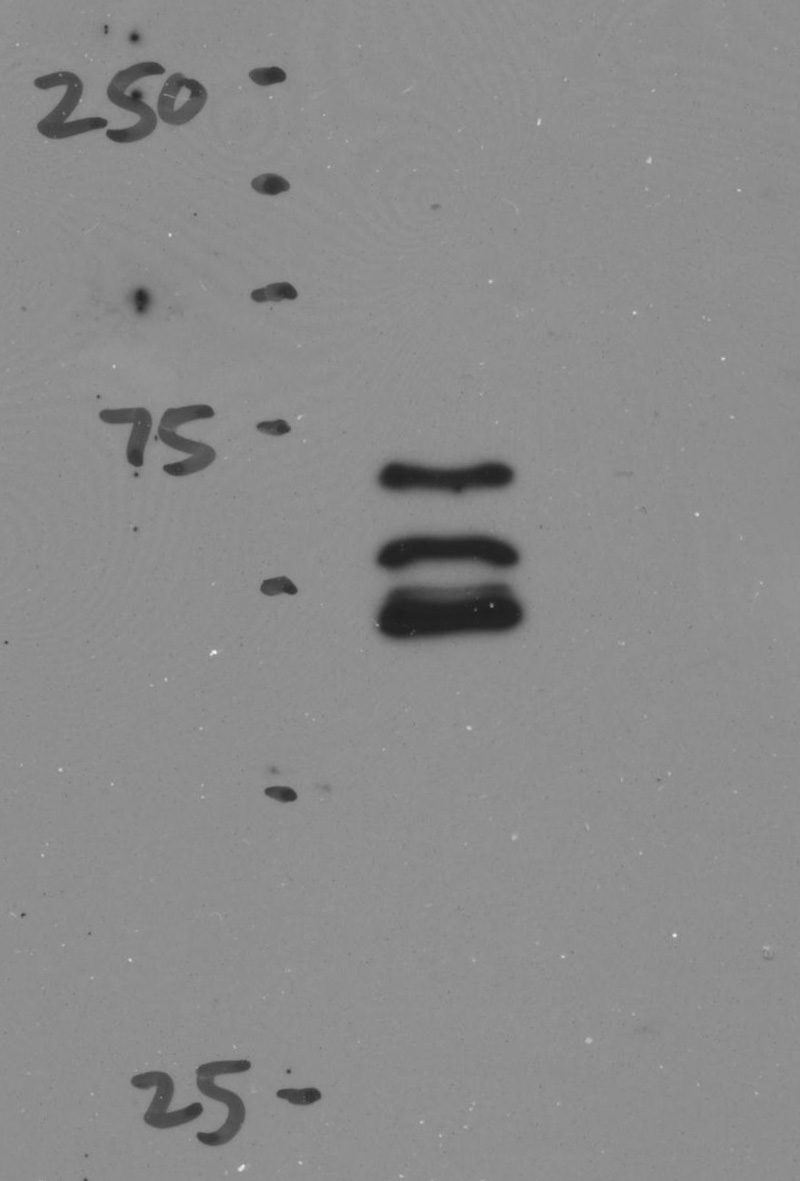

Supplement: Supplementary file 18 [file msb0011-0775-sd18.zip › Source Data for Figure 5/Fig.5G/Fig5G. 293T-shbTRCP_anti-bTRCP.jpg]

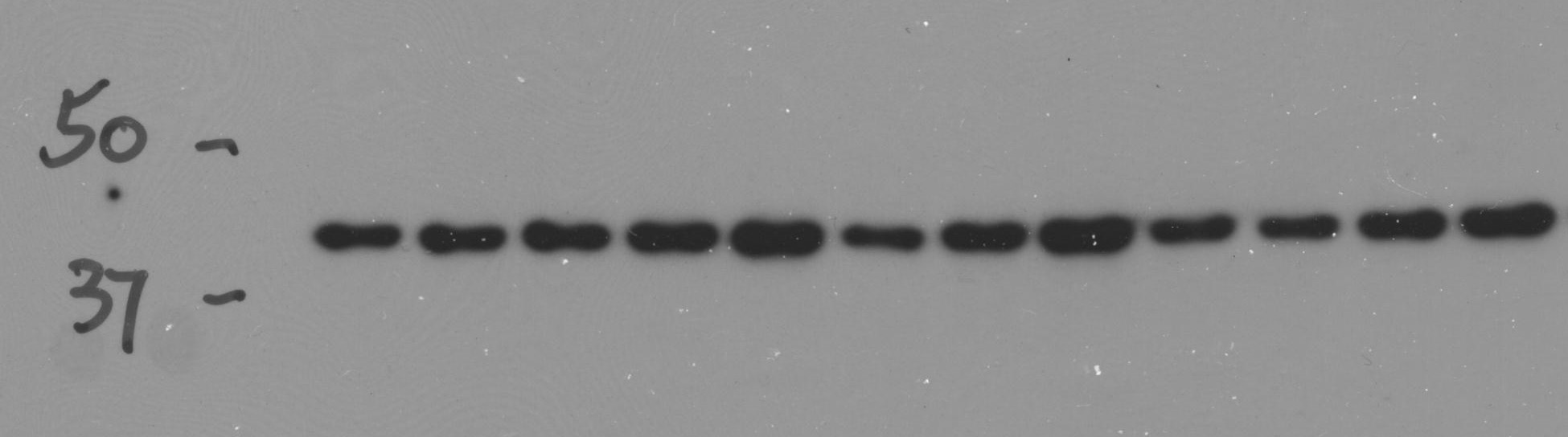

Supplement: Supplementary file 18 [file msb0011-0775-sd18.zip › Source Data for Figure 5/Fig.5G/Fig5G. 293T-shCtrl & 293T-shCUL1_anti-bActin.jpg]

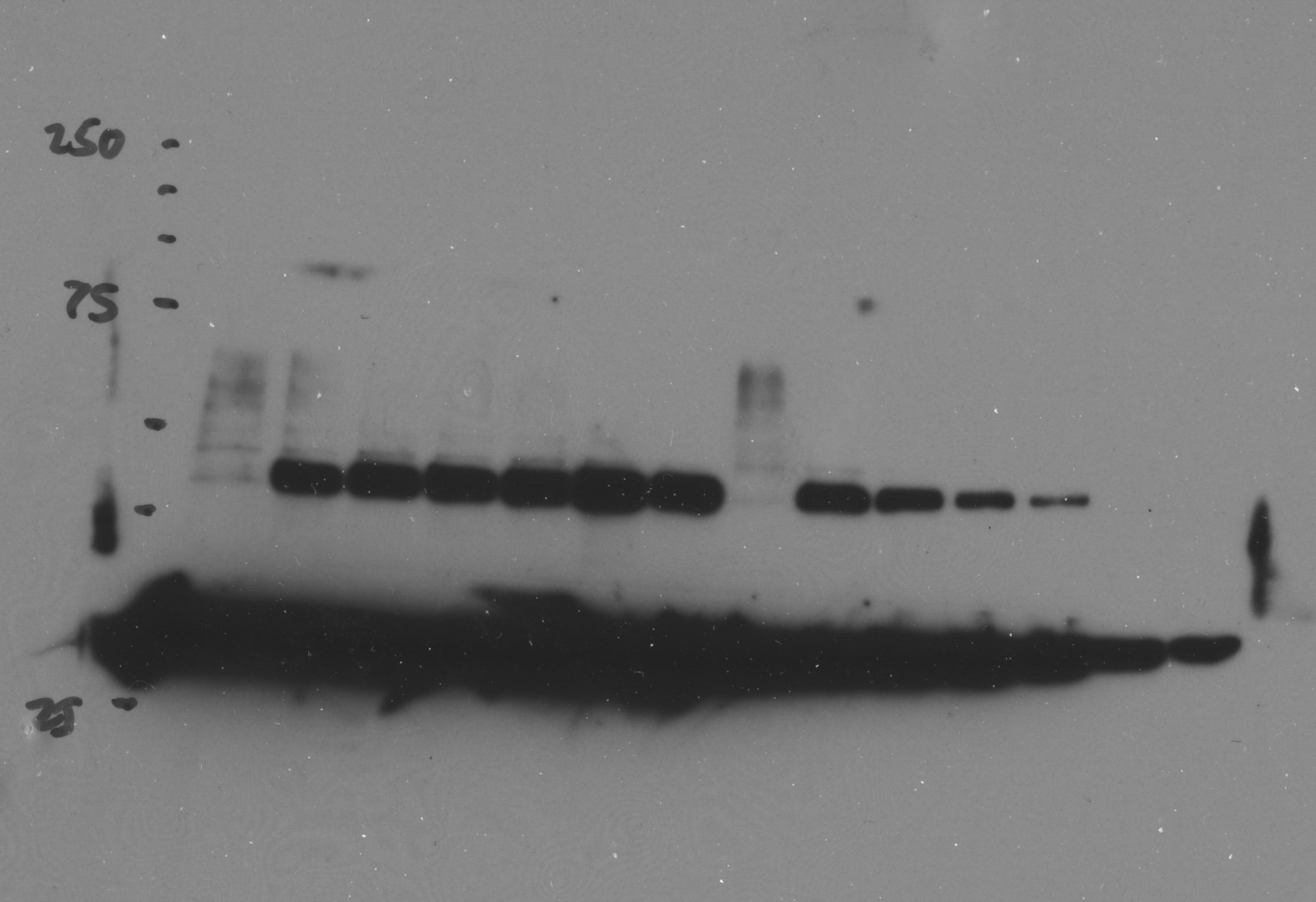

Supplement: Supplementary file 18 [file msb0011-0775-sd18.zip › Source Data for Figure 5/Fig.5G/Fig5G. 293T-shCtrl & 293T-shCUL1_anti-FOXN2.jpg]

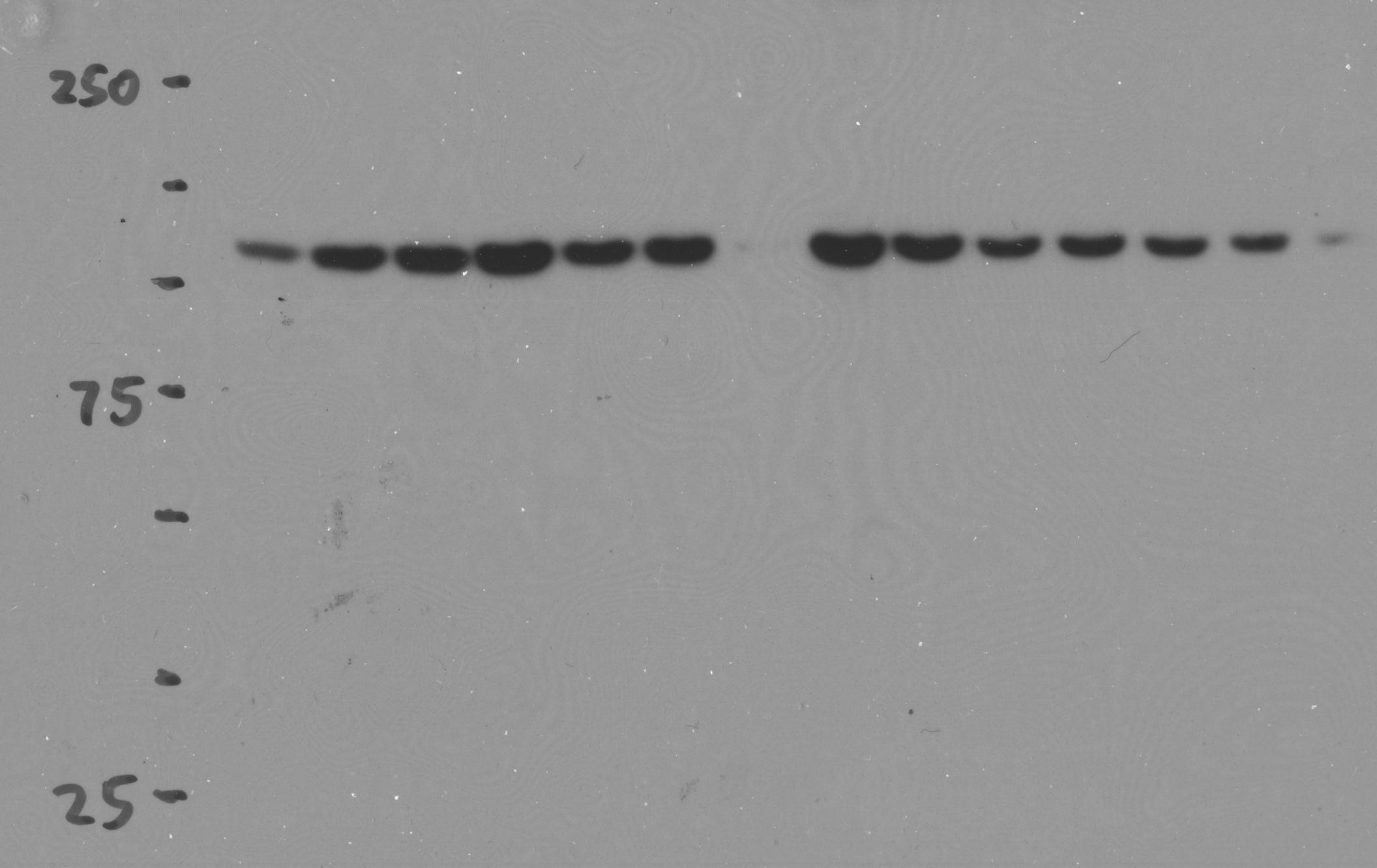

Supplement: Supplementary file 19 [file msb0011-0775-sd19.zip › Source Data for Figure 6/Fig.6B/Fig6B. Input-_Anti-MYC.jpg]

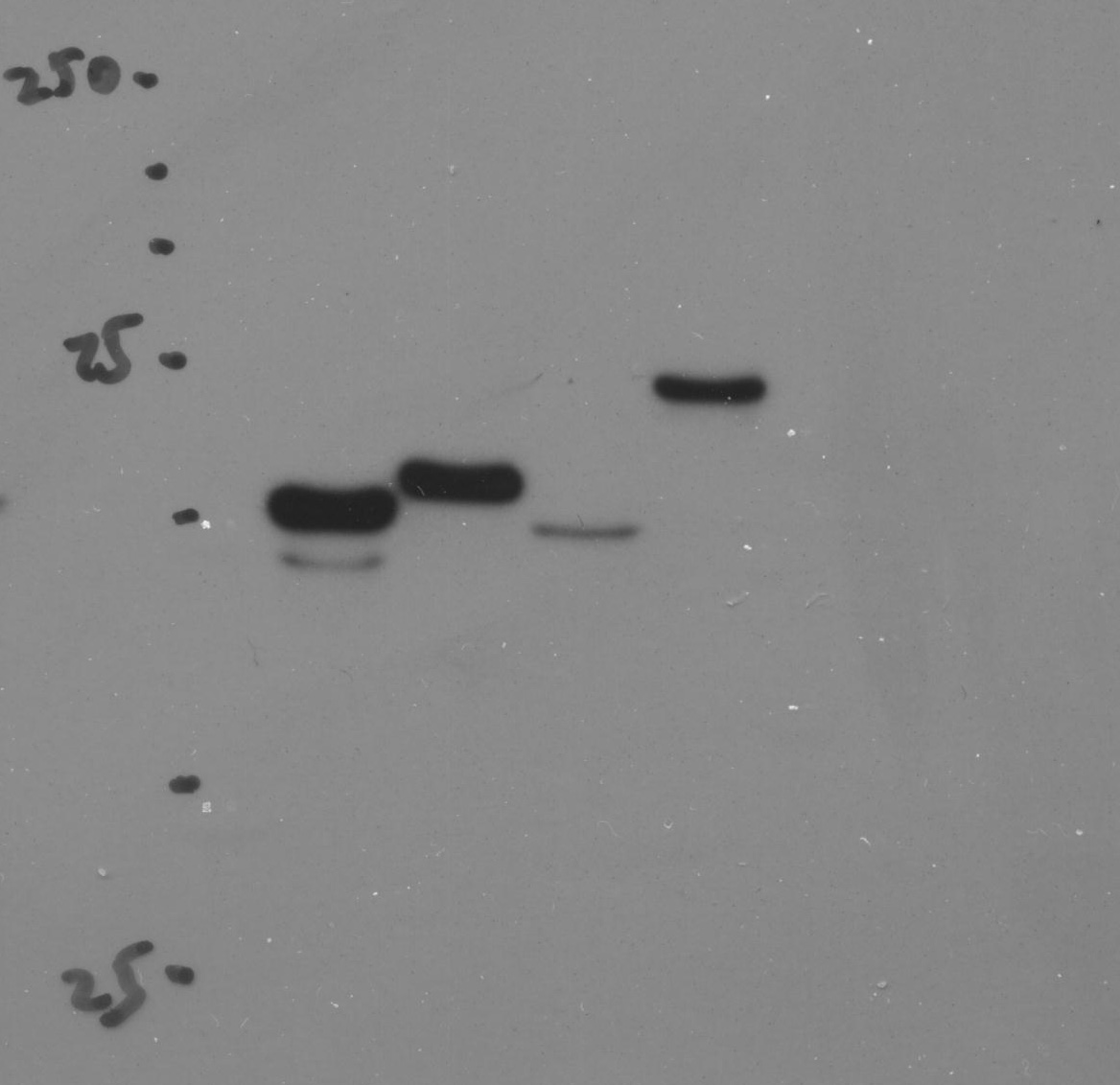

Supplement: Supplementary file 19 [file msb0011-0775-sd19.zip › Source Data for Figure 6/Fig.6B/Fig6B. Input-Chromatin_Anti-FLAG.jpg]

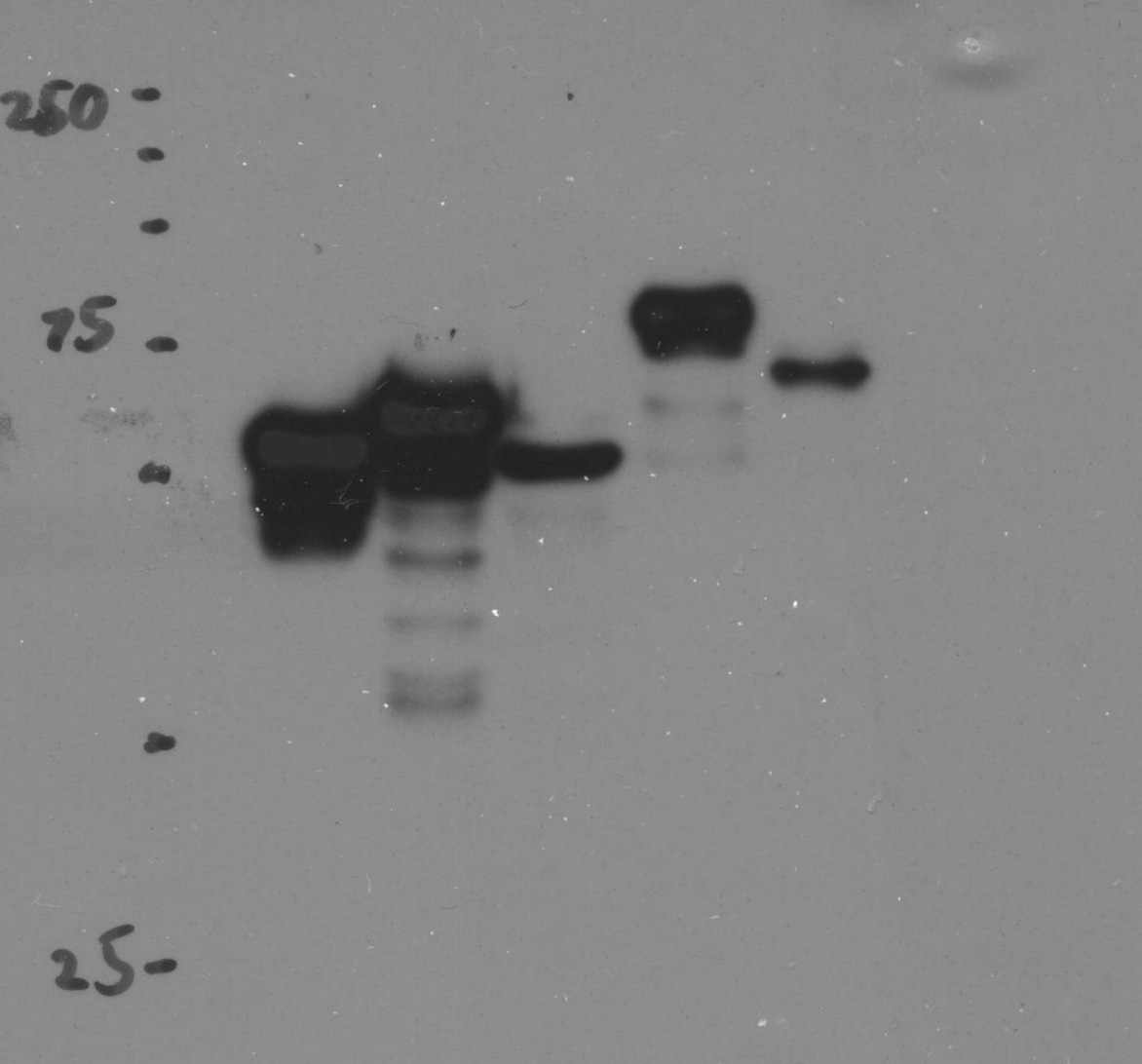

Supplement: Supplementary file 19 [file msb0011-0775-sd19.zip › Source Data for Figure 6/Fig.6B/Fig6B. Input-Soluble_Anti-FLAG.jpg]

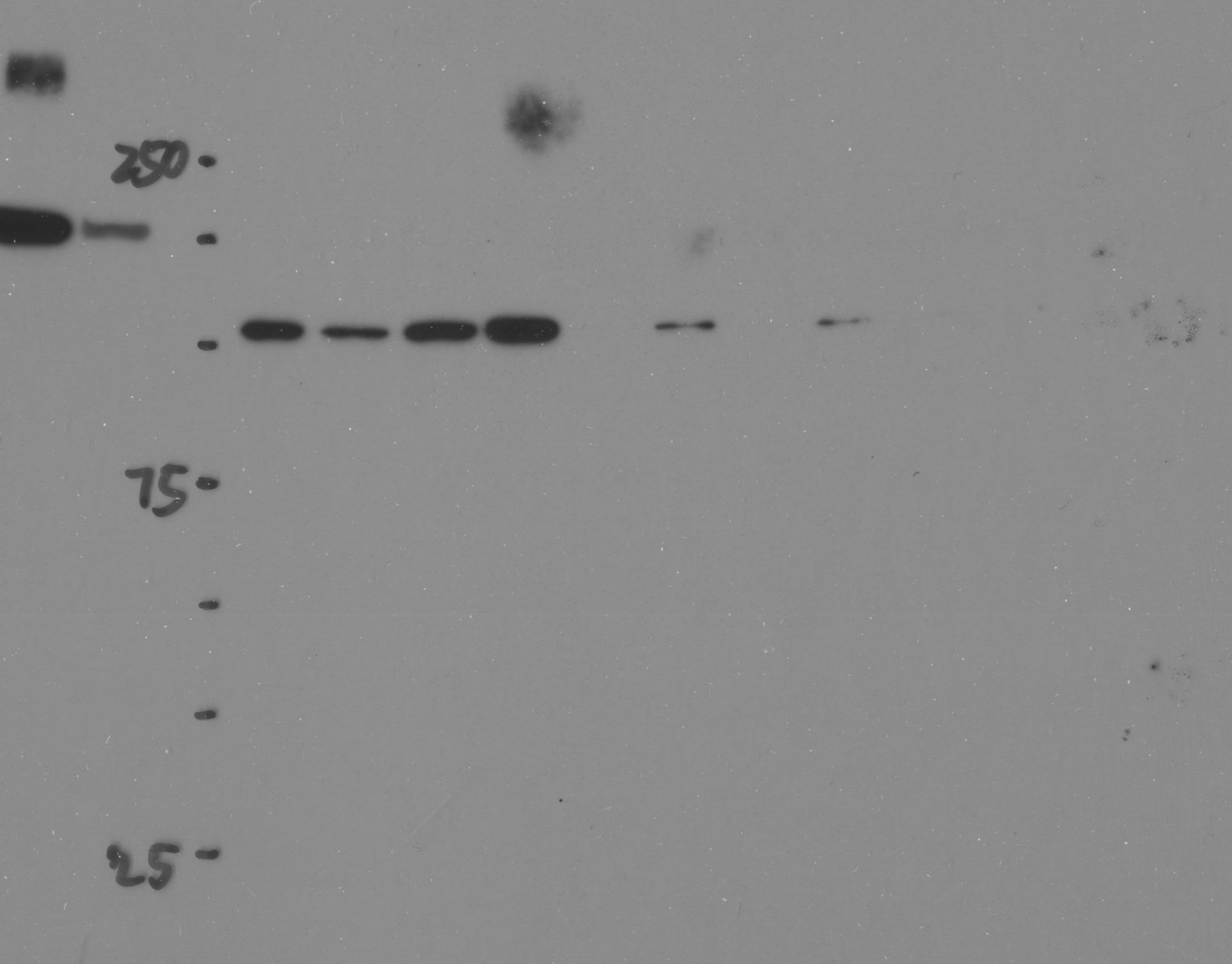

Supplement: Supplementary file 19 [file msb0011-0775-sd19.zip › Source Data for Figure 6/Fig.6B/Fig6B. Pulldown-S-beads_Anti-MYC.jpg]

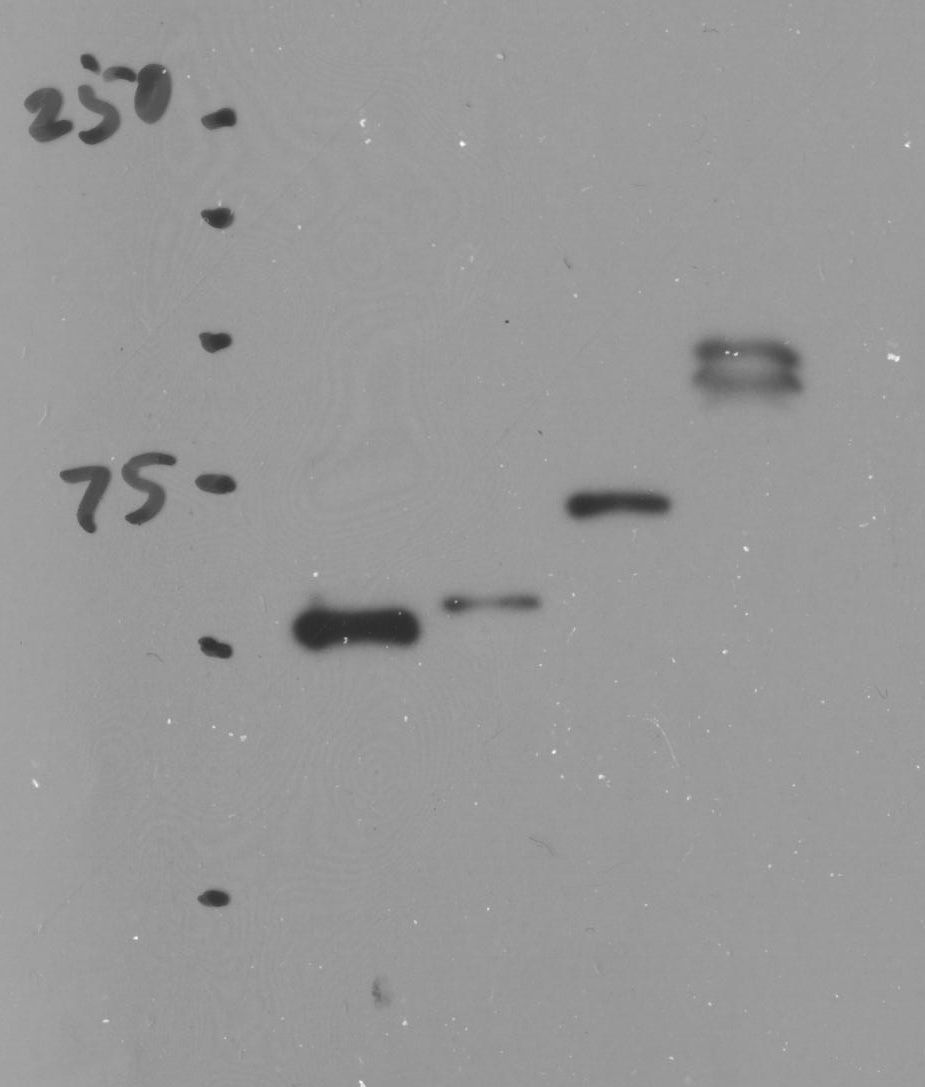

Supplement: Supplementary file 19 [file msb0011-0775-sd19.zip › Source Data for Figure 6/Fig.6C/Fig6C. Input-Chromatin_Anti-FLAG.jpg]

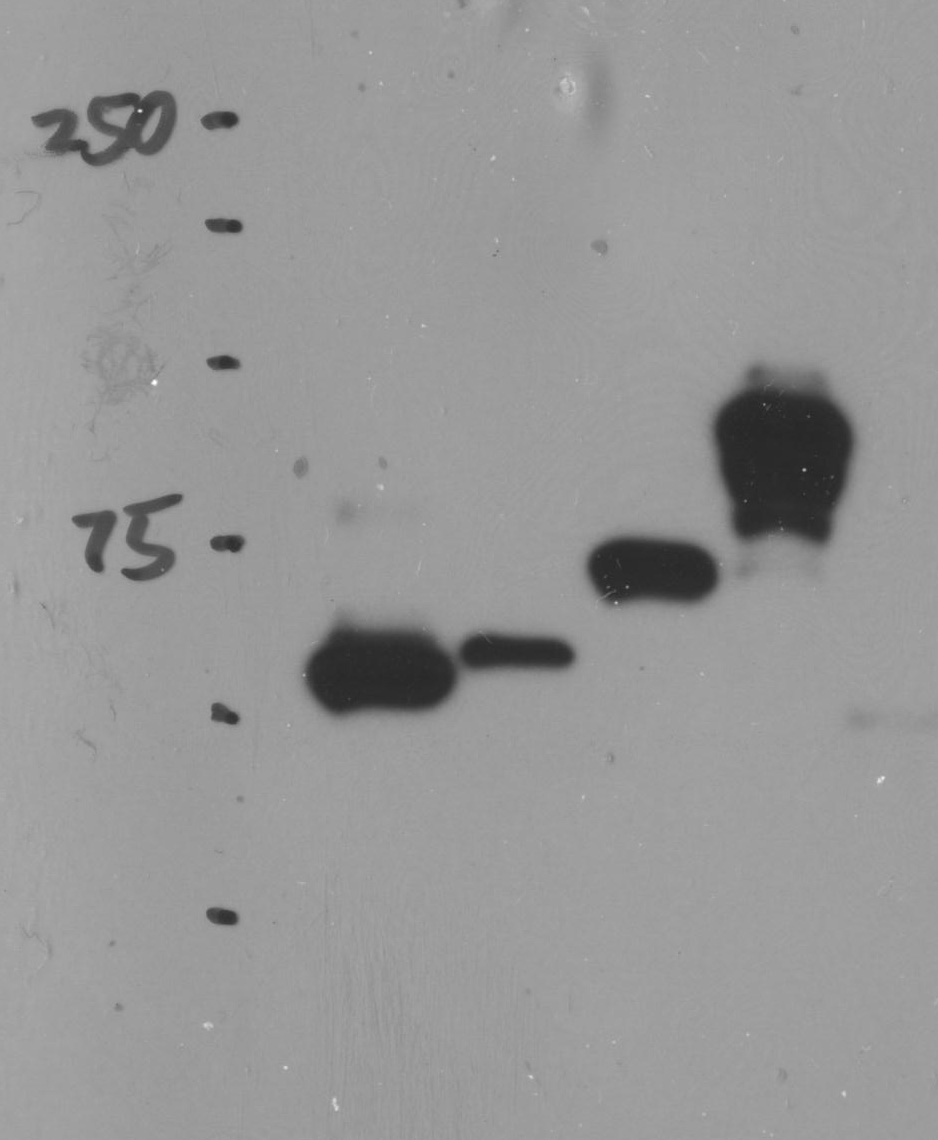

Supplement: Supplementary file 19 [file msb0011-0775-sd19.zip › Source Data for Figure 6/Fig.6C/Fig6C. Input-Soluble_Anti-FLAG.jpg]

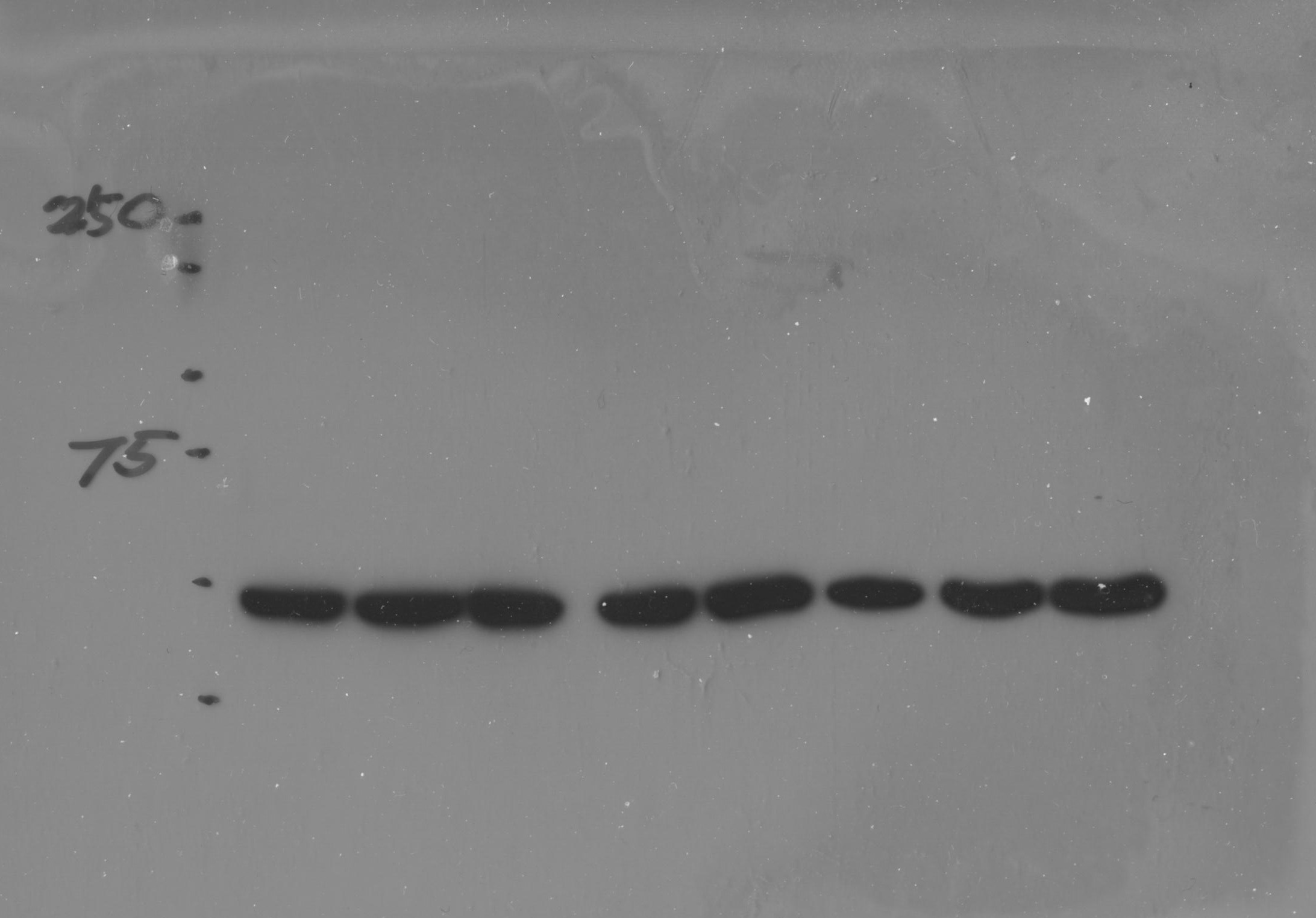

Supplement: Supplementary file 19 [file msb0011-0775-sd19.zip › Source Data for Figure 6/Fig.6C/Fig6C. Input_Anti-MYC.jpg]

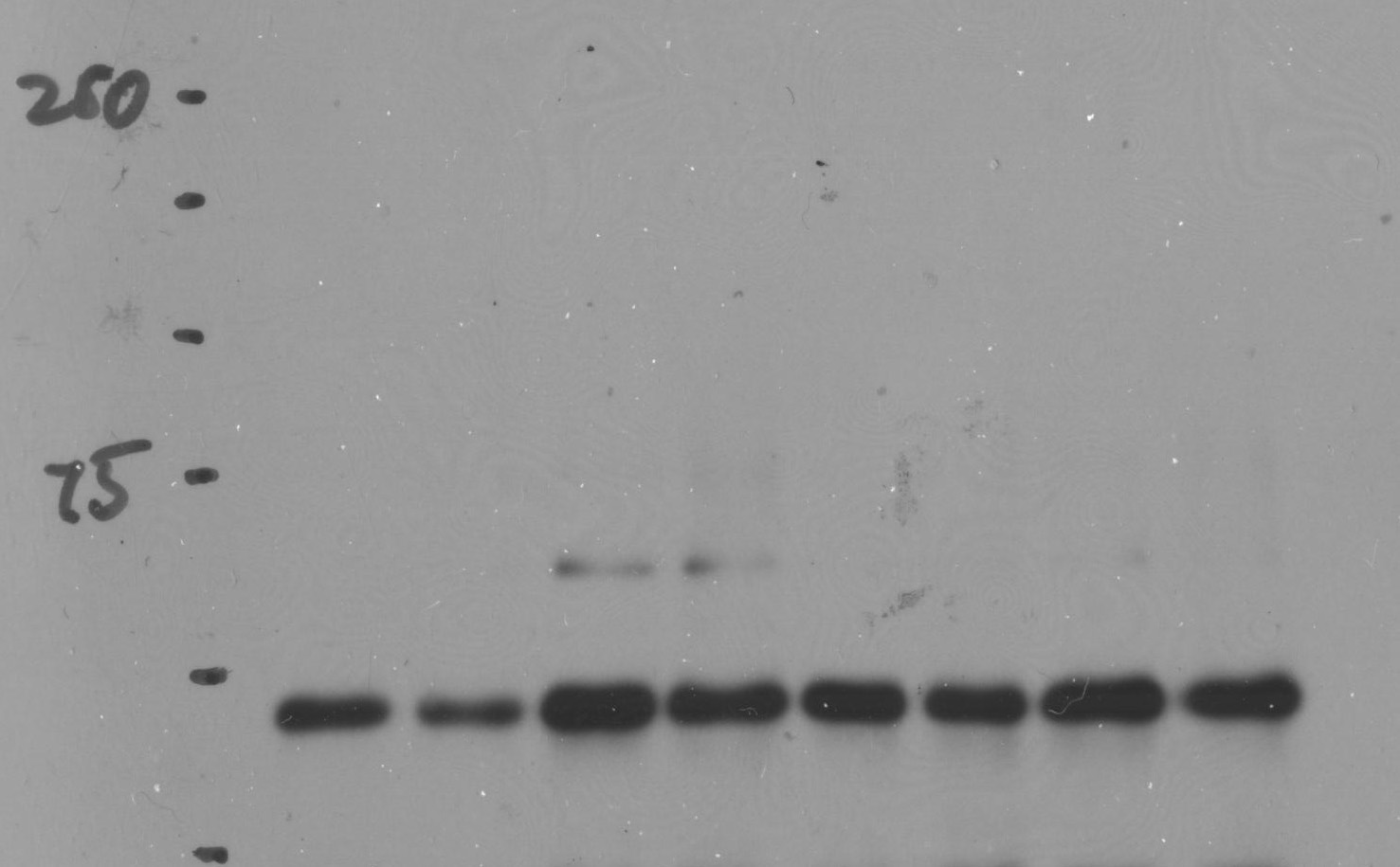

Supplement: Supplementary file 19 [file msb0011-0775-sd19.zip › Source Data for Figure 6/Fig.6C/Fig6C. Pulldown_Anti-MYC.jpg]
