# Supplementary material for: Proteomic analyses reveal distinct chromatin-associated and soluble transcription factor complexes
Source: Mol Syst Biol. 2015 Jan 21;11(1):775. doi: 10.15252/msb.20145504 (PMC4332150; doi:10.15252/msb.20145504)
Supplement: Supplementary file 21 [file msb0011-0775-sd21.docx]

**Supplementary Legends**

Location Specific Complexomes for Transcriptional Regulation

Xu Li^1,6^, Wenqi Wang^1,6^, Jiadong Wang^1^, Anna Malovannaya^2^, Yuanxin Xi^2,3^, Wei Li^2,3^, Rudy Guerra^4^, David H. Hawke^5^, Jun Qin^2^, and Junjie Chen^1,*^

^1^Department of Experimental Radiation Oncology, The University of Texas MD Anderson Cancer Center, Houston, TX77030, USA

^2^Department of Molecular and Cellular Biology,

^3^Division of Biostatistics, Dan L. Duncan Cancer Center, Baylor College of Medicine, Houston, TX, 77030, USA

^4^Department of Statistics, Rice University, Houston, TX77005, USA

^5^Department of Systems Biology, Department of Pathology, The University of Texas MD Anderson Cancer Center, Houston, TX77030, USA

^6^These authors contributed equally

^*^Corresponding author

Department of Experimental Radiation Oncology, The University of Texas MD Anderson Cancer Center, 1515 Holcombe Boulevard - Unit 66 (Room Y3.6006), Houston, Texas 77030
Ph:  713-792-4863; Fx:  713-745-6141

Correspondence to: [jchen8@mdanderson.org](mailto:jchen8@mdanderson.org)

**This PDF file includes:**

Supplementary Figures:

Figure S1 is related to Figure 1, which shows examples of our tandem affinity purifications.

Figure S2 is related to Figure 1, which shows the preys specificities in different groups and fractions.

Figure S3 is related to Figures 2 and 3, which shows data validation of HCIPs.

Figure S4 is related to Figure 4, which provides the gene ontology (GO) process analysis of human transcription factors in molecular & cellular functions and human diseases.

Supplementary Tables:

Table S1 is related to Figures 1 and 2, which lists the bait TFs and their involvement in cancer related and other signalling pathways and diseases.

Table S2 is related to Figures 1 and S1, which lists the bait TFs and their subcellular localizations.

Table S3 is related to Figures 1, 2 and 3, which lists original protein identification data of our human transcription factors proteomics studies.

Table S4 is related to Figures 1, 2 and 3, which lists original peptide identification data of our human transcription factors proteomics studies.

Table S5 is related to Figures 2 and S2, which lists the original data for atlas abundance and specificity scores in different group and fractions.

Table S6 is related to Figures 2 and 3, which lists HCIPs of the human transcription factors, data validation, and prey annotations.

Table S7 is related to Figures 2, which provides interaction overlaps of 24 repeated experiments.

Table S8 is related to Figure 3, which provides a complete post-translational modifications list of peptides.

Table S9 is related to Figures 3 and 6, which provides JUN AP-MS results using antibodies against endogenous proteins.

Table S10 is related to Figure 1 and 6, which provides the pathway annotations of TFs based on literature reports and HCIPs identified in this study.

Table S11 is related to Figure 1 and discussion, which lists TFs HCIP sets alterations in different cancer databases.

Table S12 is related to discussion, which provides NFκB1 re-TAP-MS results.

Supplementary Dataset S1 is related to Figures 3, which is the cytoscape.cys file illustrating the TF protein interaction networks in chromatin and soluble fractions, based on the HCIP datasets generated in this study.

**Supplementary Figure Legends**

**Figure S1. Establishing HEK293T cell lines stably expressing bait proteins for TAP-MS analysis.**

(**A-B**) Constructs encoding C-terminal triple-tag (SBP-tag, S-tag, and FLAG-tag) fused bait proteins were used to establish HEK293T-derivative cell lines stably expressing these proteins. The expression of bait proteins was confirmed by immunostaining and immunoblotting with anti-FLAG antibodies to ensure the proper localization (A) and expression (B), respectively. (**B**) Western blotting with antibodies recognizing endogenous proteins was conducted for 12 baits to ensure comparable expression of exogenous proteins with endogenous proteins. (**C**) TAP-MS was performed on both chromatin (Ch) and soluble (S) fractions of each protein; examples of some of these purifications are presented here. SDS-page gels were stained with coomassie blue. (**D**) 293T cells were treated with vehicle or 10µM LY294002 for 16 hours, and blotted with antibodies targeting FOXO3 and Histone H3.

**Figure S2. Analysis of prey specificity.**

(**A**) Prey specificities in different groups and fractions. The y axis shows prey specificity comparing with control random protein purifications. Positive numbers indicate that they specifically bind to baits in the experimental group, and negative numbers indicate that they have no preference or are enriched the control group. The x axis indicates the abundance of individual preys. (**B**) Prey specificities in the chromatin fraction. The y axis shows prey appearance specificity in the chromatin fractions. Positive numbers indicate that they specifically show up in the chromatin fraction, and negative numbers indicate that they have no preference or are specifically enriched in the soluble fraction. The x axis indicates the abundance of individual preys. (**C**) Prey specificities in the soluble fraction. The y axis shows appearance specificity in the soluble fraction.

**Figure S3. Validation of FOXM1, MAX and FOXO3 HCIPs.**

(**A, B, D**) 293T cells were transfected with constructs encoding SFB-tagged HCIPs of FOXM1 (A), MAX (B) and FOXO3 (D) as indicated. Pulldown experiments were carried out with S-protein beads and immunoblotted with antibodies against FOXM1 (A), MAX (B) and FOXO3 (D) as indicated. (**C**) 293T cells were infected with shRNA targeting MAX HCIPs. Four shRNAs targeting the same protein were packaged independently and stable cells are selected using puromycin. Cells were immunostained with Ki67 antibody and DAPI. Slides were mounted and live-visualized using a Nikon ECLIPSE E800 fluorescence microscope with a Nikon Plan Fluor 10× objective lens, and subjected to auto object count (Roper Scientific). Object parameters were set between 10 µM and 30 µM, n=4. (**E**) 293T cells were infected with shRNA targeting FOXO3 HCIPs. Four shRNAs targeting the same protein were packaged independently and stable cells are selected using puromycin. Cells were treated with vehicle or 10µM LY294002 for 16 hours. GADD45A mRNA level was evaluated with real-time PCR, n=4. (**F**) MCF10A, MCF10-shFOXK1 and MCF10A-FOXK1 cells were treated with vehicle or 10µM LY294002 for 16 hours and immunostained with FOXO3 antibody and DAPI, n=4.

**Figure S4. Pathway enrichment annotations based on individual interactome and disease correlation.**

Pathway enrichment was performed on RBPJ (A) and FOXO3 (B) based on their HCIPs identified in chromatin or soluble fractions and literatures. The x-axis indicates the –log (*P* value) of enrichment. “TF-Literature” indicates the signalling pathways annotation based on the literature reports. “TF-HCIP” here means the signalling pathways annotation based on the HCIPs obtained from our dataset.

**Supplementary Datasets**

**Supplementary Dataset S1. Human TFs Interactomes based on HCIP datasets generated in this study.**

The cytoscape.cys file illustrating the overall TF protein interaction networks in chromatin and soluble fractions, based on the HCIP dataset generated in this study.

**Supplementary Tables**

**Table S1. Bait TFs and their involvement in signalling pathways and diseases**.

We listed the 56 bait transcription factors used in our study and their involvement in signalling pathways and disease, as well as their expressions in HEK293T cells.

**Table S2. Bait TFs and their subcellular localizations.**

We listed the 56 bait transcription factors used in our study and their subcellular localizations in our HEK293T stable cells, suggested localizations by literatures, and the literature resources.

**Table S3. The complete protein identification lists.**

The complete protein identifications for 120 purifications of human transcription factors, 24 replicate purifications, and 70 control purifications are listed here. The experiment number for each bait protein was shown in the first row, which helps the search for the raw data files for specific bait protein.

**Table S4. The complete peptide identification lists.**

The complete peptide identifications for 214 experiments are listed here.

**Table S5. Prey specificity and abundance scores.**

We used the algorithms specified in our method section to calculate the prey appearance and enrichment. 3,714 preys identified in 120 purifications of transcription factors were assigned with specificities for TF interaction (Mu_TF), specificities in chromatin fraction (Mu_CHR), specificities in soluble fraction (Mu_SOL), and abundance score (Alpha). This table was used to generate Fig. 2A and Supplementary Fig. S2.

**Table S6. HCIPs of the human transcription factors, data validation, and prey annotations.**

The 2156 HCIPs identified for the human transcription factors are listed here. The bait name, HCIP prey, spectra counts (SpectraC) and SAINT score (SS) are indicated. We searched these HCIPs in different databases to identify interactions reported in the literature. These databases include: BioGrid (<http://thebiogrid.org/>) ([Stark et al, 2006](#_ENREF_7)), STRING (<http://string-db.org/>) ([von Mering et al, 2003](#_ENREF_8)), BIND (<http://bind.ca>) ([Bader et al, 2003](#_ENREF_1)), DIP (<http://dip.doe-mbi.ucla.edu>) ([Xenarios et al, 2000](#_ENREF_9)), HPRD (<http://www.hprd.org/>) ([Prasad et al, 2009](#_ENREF_5)), C. Elegans TF ([Reece-Hoyes et al, 2013](#_ENREF_6)), and CCI ([www.epicome.org](http://www.epicome.org)) ([Malovannaya et al, 2011](#_ENREF_4)). We also annotated the HCIPs or preys and included the prey Entrez gene name, localization, family and functions.

**Table S7. Interaction overlaps of HCIPs with 24 repeated experiments.**

Interaction overlaps of HCIPs with 24 repeated experiments. “SELF” indicates bait self-identifications; “HCIP” indicates they have also shown in HCIP list in repeated experiments; “Unfiltered” indicates they have shown in the unfiltered list in repeated experiments.

**Table S8. The complete post-translational modifications list.**

“TF-Disease-Literature” indicates the disease correlation annotation based on the literature reports. “TF-Disease-New” here means the new disease correlation annotation based on the non-self HCIPs obtained from our dataset. The *P* values are estimated using the Knowledge Base provided by Ingenuity pathway software (Ingenuity Systems, [www.ingenuity.com](http://www.ingenuity.com)), which contains findings and annotations from multiple sources including the Gene Ontology database. Only the statistical significant correlations are shown.

**Table S7. Interaction overlaps of HCIPs with 24 repeated experiments.**

Interaction overlaps of HCIPs with 24 repeated experiments. “SELF” indicates bait self-identifications; “HCIP” indicates they have also shown in HCIP list in repeated experiments; “Unfiltered” indicates they have shown in the unfiltered list in repeated experiments.

**Table S8. Complete post-translational modifications list of peptides.**

Peptide sequences (and hence protein identity) were determined by matching protein databases with the acquired fragmentation pattern by the Mascot in Proteome discoverer 1.4. (ThermoFisher, San Jose, CA). Enzyme specificity was set to partially tryptic with 2 missed cleavages. Mass tolerance was set to 2.0 for precursor ions and 1.0 for fragment ions. The database searched was the Human IPI databases version 3.6. Peptides were searched with phosphorylation (S, T) and acetylation (K) for identification; and oxidation (M), S-carboxamidoethyl-L-cysteine (C) and iodoacetamide (C) for modifications during sample processing. Peptide confidence: High: FDR < 0.01, Medium: 0.01 < FDR < 0.05.

**Table S9. JUN AP-MS result using antibodies against endogenous proteins**

A total of 5 × 10^7^ HEK293T cells were fractionated and subjected to affinity purification using antibodies against endogenous JUN (Cell Signaling #9163S) followed by MS analysis.

**Table S10. Pathway enrichment of TF HCIP datasets.**

TFs were enriched in pathway analysis using their HCIP sets. The *P* values were estimated using the Knowledge Base provided by Ingenuity pathway software (Ingenuity Systems, [www.ingenuity.com](http://www.ingenuity.com)), which contains findings and annotations from multiple sources including the Gene Ontology database, KEGG and Panther pathways. Only the statistical significant correlations (*P* < 0.01) are shown.

**Table S11. TFs HCIP sets alterations in different cancer databases.**

TFs were searched for their alteration case numbers and rates in cBioPortal database ([Cerami et al, 2012](#_ENREF_2); [Gao et al, 2013](#_ENREF_3)) using their HCIP sets. Overall: the overall alteration rates. Mutation: the mutation rates. Amplification or Deletion: the non-mutational alteration rates.

**Table S12. NFκB1 re-TAP-MS.**

A total of 1 × 10^8^ HEK293T cells stably expressing NFκB1 were fractionated and subjected to standard tandem affinity purification followed by MS analysis.

**REFERENCES**

Bader GD, Betel D, Hogue CW (2003) BIND: the Biomolecular Interaction Network Database. *Nucleic Acids Res* **31:** 248-250

Cerami E, Gao J, Dogrusoz U, Gross BE, Sumer SO, Aksoy BA, Jacobsen A, Byrne CJ, Heuer ML, Larsson E, Antipin Y, Reva B, Goldberg AP, Sander C, Schultz N (2012) The cBio cancer genomics portal: an open platform for exploring multidimensional cancer genomics data. *Cancer Discov* **2:** 401-404

Gao J, Aksoy BA, Dogrusoz U, Dresdner G, Gross B, Sumer SO, Sun Y, Jacobsen A, Sinha R, Larsson E, Cerami E, Sander C, Schultz N (2013) Integrative analysis of complex cancer genomics and clinical profiles using the cBioPortal. *Sci Signal* **6:** pl1

Malovannaya A, Lanz RB, Jung SY, Bulynko Y, Le NT, Chan DW, Ding C, Shi Y, Yucer N, Krenciute G, Kim BJ, Li C, Chen R, Li W, Wang Y, O'Malley BW, Qin J (2011) Analysis of the human endogenous coregulator complexome. *Cell* **145:** 787-799

Prasad TS, Kandasamy K, Pandey A (2009) Human Protein Reference Database and Human Proteinpedia as discovery tools for systems biology. *Methods Mol Biol* **577:** 67-79

Reece-Hoyes JS, Pons C, Diallo A, Mori A, Shrestha S, Kadreppa S, Nelson J, Diprima S, Dricot A, Lajoie BR, Ribeiro PS, Weirauch MT, Hill DE, Hughes TR, Myers CL, Walhout AJ (2013) Extensive rewiring and complex evolutionary dynamics in a C. elegans multiparameter transcription factor network. *Mol Cell* **51:** 116-127

Stark C, Breitkreutz BJ, Reguly T, Boucher L, Breitkreutz A, Tyers M (2006) BioGRID: a general repository for interaction datasets. *Nucleic Acids Res* **34:** D535-539

von Mering C, Huynen M, Jaeggi D, Schmidt S, Bork P, Snel B (2003) STRING: a database of predicted functional associations between proteins. *Nucleic Acids Res* **31:** 258-261

Xenarios I, Rice DW, Salwinski L, Baron MK, Marcotte EM, Eisenberg D (2000) DIP: the database of interacting proteins. *Nucleic Acids Res* **28:** 289-291
