# Supplementary material for: Proteomic analyses reveal distinct chromatin-associated and soluble transcription factor complexes
Source: Mol Syst Biol. 2015 Jan 21;11(1):775. doi: 10.15252/msb.20145504 (PMC4332150; doi:10.15252/msb.20145504)
Supplement: Supplementary file 17 [file msb0011-0775-sd17.pdf]

Proteomic analyses reveal distinct chromatin-associated and soluble transcription factor complexes

Xu Li, Wenqi Wang, jiadong Wang, Anna Malovannaya, Yuanxin Xi, Wei Li, Rudy Guerra, David H Hawke, Jun Qin and Junjie Chen

Corresponding author: Junjie Chen, The University of Texas MD Anderson Cancer Center

Review timeline:

Submission date:	18 June 2014
Editorial Decision:	15 July 2014
Revision received:	10 October 2014
Editorial Decision:	17 November 2014
Revision received:	25 November 2014
Accepted:	08 December 2014

Editor: Maria Polychronidou

Transaction Report:

(Note: With the exception of the correction of typographical or spelling errors that could be a source of ambiguity, letters and reports are not edited. The original formatting of letters and referee reports may not be reflected in this compilation.)

1st Editorial Decision 15 July 2014

Thank you again for submitting your work to Molecular Systems Biology. We have now heard back from the three referees who agreed to evaluate your manuscript. As you will see from the reports below, the referees find the topic of your study potentially interesting. They raise, however, substantial concerns on your work, which should be convincingly addressed in a revision of the manuscript.

One of the major points refers to the need to provide additional validations for the presented results. Moreover, further experimentation demonstrating the functional relevance of some of the interactions (i.e. extending the analysis of FOXN2 interactions) would significantly enhance the impact of this work.

We would like to point out that we do not agree with the comment of reviewer #2 that the data on the 19 TFs (that are presented in addition to the FOX TFs) seem redundant and we think that the data represent a potentially useful resource and should be included in the study.

On a more editorial level, we would kindly ask you to deposit the MS datasets and molecular interaction data in the appropriate public databases. (Additional information is available in the "Guide for Authors" section in our website at <http://msb.embopress.org/authorguide#a3.5>) Furthermore, we would like to ask you to include the links and accession numbers in the "Data Availability" section of your manuscript.

If you feel you can satisfactorily deal with these points and those listed by the referees, you may wish to submit a revised version of your manuscript. Please attach a covering letter giving details of the way in which you have handled each of the points raised by the referees. A revised manuscript

© European Molecular Biology Organization

will be once again subject to review and you probably understand that we can give you no guarantee at this stage that the eventual outcome will be favorable.

REFEREE REPORTS

Reviewer #1:

In this manuscript, Chen et al. report a comprehensive analysis of the complexome for transcriptional regulation. They applied tandem-affinity purification (TAP) coupled to mass spectrometry to characterize both the soluble and chromatin-associated complexes using 56 transcription factors as baits. The selection of transcription factors included the complete family of Forkhead box (FOX) and series of structurally unrelated TFs. The datasets is very likely to be very useful as it implies many signaling pathways involved in development and cancer. The dataset is impressive and the authors developed and applied a very carefully thought experimental design. This includes many biological replicates, numerous control purification with unrelated proteins - to discriminate the background - and several reciprocal, reverse purification experiments to confirm initially observed interactions. The analysis revealed the TFs in the on or off state contribute to different protein complexes. The work is solid and the results intriguing. The follow-up experiments though are a bit preliminary and disappointing. In a few cases the authors might have looked at - and compare - post-translational modifications in the in/off state, may be perform some siRNA (or kinase inhibitors, etc) experiments to address the possible functional relevance of (some) of these interactions. Also the bioinformatic analysis was limited: were the interactors conserved in other (mammalians) species? What are the expression profiles of the new interactors (different tissues and cell types)? Any mutation associated with disease status, etc? Addressing at least some of these questions may significantly raise the impact of the work.

Specific point:
1) What fraction of the TFs is expressed in the cell line used here (HEK)? This should be mentioned, as many expression data are now a day available, and the possible impact of expression in a "heterologous" system should at least be discussed.

Reviewer #2:

In this manuscript the authors describe an analysis of transcription factors and their protein interactions in HEK293C cells. The authors analyze 56 transcription factors with a particular focus on forkhead box family transcription factors and 19 additional transcription factors that are largely independent of the forkhead family transcription factors. The authors generate stable cells lines of these transcription factors with a tandem affinity tag and they analyze the soluble and chromatin associated fractions of each transcription factors protein interactions. The authors use standard and sound approaches to analyze their protein interaction network data and they compare their data to prior knowledge and they make the case for finding many new interactions in their datasets. Overall, the work is properly carried out and analyzed. However, there are several significant issues that need to be addressed prior to publication.

The first major issue is the samples used. The major focus is on the forkhead box family of transcription factors and there is potentially novel biology of importance in this data. Focusing the manuscript on this data only would strengthen the manuscript. The 19 additional transcription factors come across as an afterthought and do not add much to the manuscript. This aspect of the manuscript needs to be reconsidered. The authors carried out reciprocal purifications (data in Fig 2 F) but end with this. NFATC1 for example appears interesting in that it pulls down several transcription factors. A discussion of this and validation by CoIP or colocalization with imaging would be valuable and one way to strengthen this aspect of the manuscript. The other issue with the additional 19 TFs studied is some of these are well studied and there are missing identifications. For example, RelB is not listed in the NFKB1 datasets. What information was missing from their analysis and why?

Next, for all the purifications there are several instances where no identifications were presented in Fig3 like for FOXC4 chromatin, FOXC2 soluble, FOXJ2 soluble, ETS1 chromatin. The authors

© European Molecular Biology Organization

must discuss these samples where no identifications are provided and provide an explanation for this. Were there technical issues with their approach for these samples and if so what were they, or is there a biologicsal reason for these results? This must be discussed for their method to be fully understood by researchers.

The authors then largely focus on the forkhead box TFs and a couple of insights they found in their data. One is on a GO analysis of functions of preys for FOXM1 and the differences in the soluble and chromatin associated proteins for this TF. This data is interesting but no validation of the results is provided to show FOXM1 plays a role in the tRNA splicing complex function or regulation, for example. As it stands, this is a potential role of FOXM1, but remains speculative. The authors then provide CoIP information on FOXN2 and its interaction with FBXW11. The authors provide some data to suggest that there may be an increase in degradation upon these interactions in in Fig 5E, but this data is preliminary and more validation is needed to strengthen this aspect of the manuscript. Carefully considering the literature on FBXW11 and CUL1 and better demonstrating their ability to regulate FOXN2 would be valuable. For example, does RNAi of either of these proteins, or others in their pull down affect cellular FOXN2 levels? Additional evidence on FOXN2 and its regulation would strengthen the manuscript.

As it stands, this is a well executed analysis of transcription factor protein interactions, but the validation of these results and the potential new biological insights is lacking for a journal like Molecular Systems Biology. In addition to the other revision described, strengthening the data on FOXN2 would improve the paper sufficiently.

Reviewer #3:

This manuscript describes the protein-protein interaction network of a large number of transcription factors (TFs) identified by modified tandem-affinity-purification followed by mass spectrometry (TAP/MS). The authors obtained proteomics data of soluble and chromatin associated complexes of the 56 TFs which include targets of many signalling pathways and a complete family of Forkhead (FOX) TFs (actually only 37 members so the text needs altering). More importantly, they concluded that most of TFs form very distinct protein complexes on and off chromatin, and the location-specific protein complexes are associated with distinct regulatory properties and diverse functions of these TFS. Overall, this is a potentially useful dataset but to be useful, there are some important areas of validation required (see below) to demonstrate its robustness. If the authors want to go beyond presenting a dataset and provide novel mechanistic insights, then further experimentation is required to validate things like "location-specific complexes". If these experiments are not provided, then the title needs altering and some of the conclusions toned down.

Major comments:
(1) More evidence needs providing to validate the subfractionation methodology. For example, western blots showing the correct re-distribution of factors expected to be in the soluble and insoluble fractions. This is important so that we know exactly which parts of the cell we are dealing with. Indeed, the authors comment that they do not get a lot of histones and HMG proteins. Why are these not in the "nuclear fraction" as one might expect, and does this mean that there is another fraction that is discarded which might have TFs associated?
(2) There is a lack of critical validation experiments. While it is impractical to do for everything, some examples should be given. For example, with the available antibodies, it would be relatively easy to do for FOXM1, to show first by mass spec that the same proteins are found. Secondly western analysis with antibodies to endogenous proteins would help. This is important as the authors admit, tags can affect things, as can over-expression (although in several examples shown, the authors attempt to choose lines not vastly over-expressing TFs).
(3) Validation of the different locations by an alternative means is required. For example PLA could be used to show cytoplasmic versus nuclear interactions of endogenous proteins. ChIP can be used to show co-occupancy on chromatin.
Related to this point, there is no validation of whether the "soluble" interacting proteins affect TF activity, or indeed any of the new interactors (with the exception of minimal evidence to show low levels of polyubiquitination of FOXN2 when a protein is over-expressed).

Specific comments for the figures
1) Fig 2C could be displayed as a supplementary table.

2) Fig. 2D is confusing. ,As described in the text on page 8, the highest reproducible rate from the 24 purifications (20 % of total 120) of biological replicates is 10 counts with the lowest 44% of 2 counts. How can it be that the unreproducible rate is therefore only 10% as shown in the figure 2D?

3) In Fig.2E the majority of the HCIPs identified by the authors are unknown compared to several known databases. However, this could be due to the lack of in depth studies. Can the authors therefore select very well documented TF and compare the interaction proteins with the data obtained in this manuscript? This will help with validating the data.

4) In Figs E3, E4, the data is not that informative. Nearly every TF in either fraction brings up the terms cell cycle and development, so this does not really discriminate well (see comments about FOXM1 below). Can the authors point to more informative terms that would help with our understanding of individual TF function?

5) In the description of Fig.3B, the authors pick and choose things to suit their viewpoint. There are many other proteins that do not fit. For example, MEF2A is clearly nuclear enriched yet it shows peptides that are apparently soluble enriched. The reciprocal applies to NFATC1 which has chromatin enriched peptides but is exclusively cytoplasmic. The data description therefore needs radically altering and some explanation provided.
In this regard, FOXN2 appear completely nuclear yet in Fig.5, it seems to only be binding to soluble proteins. Are these soluble nuclear or soluble cytoplasmic?

6) In Fig.4D-F. According to the figure, FOXM1 binds HCIP on chromatin mainly functioning in cell cycle regulation, whereas in the soluble fraction, the top GO is 'RNA post transcriptional modification'. However, in the text in page 15, the authors mentioned that '...FOXM1 formed two distinct complexes on and off chromatin, but for the same biological outcome: the regulation of mitosis and the promotion of cell cycle progression'. This is misleading (See point 4 above).
In Figure 4F, for the HCIP in soluble fraction, it is indicated as 'transcription independent function' but this is likely that this might only be regulation or modification for the bait protein itself, instead of the function of the FOXM1 protein. If the authors want to invoke new functions for FOXM1, then they should show that it affects tRNA splicing.

7) It is interesting to identify the interaction proteins for FOXN2, however, they need more evidence to clarify the conclusion. In Fig.5B, to confirm that both FOXN2 and RFX1 interact on chromatin, ChIP or even re-ChIP should be performed. For immunopricipitation, ideally, endogenous proteins should be detected. Chromatin fractionation experiments should be performed in some validation experiments.

8) Fig 6C - Based on the identified protein-protein interaction data, the authors created a new disease correlation network, which is quite different from the one shown in Fig 1A, see Ets1, TP53 and Max etc. This will cause confusion and misunderstanding for the potential function TFs, and it is not clear that interacting proteins (rather than regulated genes) will provide a useful readout of biological function/disease association (see other comments above about generally useless GO terms such as cell cycle and development).

Other minor and textual changes:
(1) Did the authors use any stimuli to make TFs move to the nucleus like NFAT or NFkB etc which are clearly predominantly cytoplasmic? If not, then what is the relevance of the nuclear fraction for these TFs? Careful presentation and discussion of this point is needed.
(2) On page 9, the text on MAX/FOXM1 interactions is not clear. Did you tag all the interactors and then flip the experiment or did you just repeat the MAX/FOXM1 IPs? Where is this data?
(3) The font size in Fig. 3A will be impossible to read in the final sized paper figures.
(4) Page 10. In addition to Sadisvam et al., 2012, there are two more papers that should be cited that link FOXM1 to the MMB complex. Grant et al. Mol Biol Cell. 2013 Dec;24(23):3634-50. Mol Cell Biol. 2013 Jan;33(2):227-36.
(5) Different colours for the lines in Fig. 6C should be selected for the benefit of colour blind scientists.

(6) On page 3, the authors should cite a paper from the Taipele lab which identifies PWMs for all Forkhead TFs. Cell. 2013 Jan 17;152(1-2):327-39.

1st Revision - authors' response 10 October 2014

Point-to-point response to reviewers

Reviewer #1:

In this manuscript, Chen et al. report a comprehensive analysis of the complexome for transcriptional regulation. They applied tandem-affinity purification (TAP) coupled to mass spectrometry to characterize both the soluble and chromatin-associated complexes using 56 transcription factors as baits. The selection of transcription factors included the complete family of Forkhead box (FOX) and series of structurally unrelated TFs. The datasets is very likely to be very useful as it implies many signaling pathways involved in development and cancer. The dataset is impressive and the authors developed and applied a very carefully thought experimental design. This includes many biological replicates, numerous control purification with unrelated proteins - to discriminate the background - and several reciprocal, reverse purification experiments to confirm initially observed interactions. The analysis revealed the TFs in the on or off state contribute to different protein complexes. The work is solid and the results intriguing. The follow-up experiments though are a bit preliminary and disappointing. In a few cases the authors might have looked at - and compare - post-translational modifications in the in/off state, may be perform some siRNA (or kinase inhibitors, etc) experiments to address the possible functional relevance of (some) of these interactions. Also the bioinformatic analysis was limited: were the interactors conserved in other (mammalians) species? What are the expression profiles of the new interactors (different tissues and cell types)? Any mutation associated with disease status, etc? Addressing at least some of these questions may significantly raise the impact of the work.

Thank you for the nice summary and suggestions. We have now performed additional bioinformatics analysis and experiments to strengthen our manuscript.

First, we have searched post-translational modifications including phosphorylation and acetylation in all our MS results (Table E8, page 10-11). We identified 8,043 peptides been modified by phosphorylation or acetylation. We identified 6,842 phosphorylation sites and 4,384 acetylation sites. We have also compared the PTMs in the two fractions. 47% of the modified peptides only exist in one fraction. Since this difference may be caused by limited representation of prey peptides, we compared the PTMs of bait proteins, which have relative higher representation in our dataset. 36% of the modified bait peptides only exist in one fraction. These results indicate that PTMs of TF protein complexes also differ in soluble and chromatin fractions.

We have also performed shRNA screening targeting the novel HCIPs of MAX and FOXO3, using respectively Ki67 staining and GADD45A mRNA level as readouts, to provide further validation that these HCIPs indeed regulate the functions of bait TFs. We found that 14 out of 20 of MAX HCIPs modulate cell growth/proliferation by at least 1.5 folds as evaluated by Ki67 staining (Fig. E3C, page 10), and 6 out of 8 of FOXO3 HCIPs changed FOXO3 downstream target gene GADD45A mRNA level by at least 1.5 folds (Fig. E3E, page 10). From FOXO3 HCIPs, we chose FOXK1 for further validation. We found that FOXK1 could regulate FOXO3 subcellular localization, since overexpression of FOXK1 translocated FOXO3 to the nucleus, while knocking down FOXK1 reduced the FOXO3 nuclear translocation upon treatment with PI3K inhibitor LY294002 (Fig. E3F, page 10).

We have also improved our bioinformatics analysis. We have obtained HCIP datasets expression alteration and mutation profiling in multiple TCGA databases (Table E11, page 14). Instead of general GO analysis in Cellular and molecular functions, we have searched pathway enrichments for HCIP sets, which we hope is more specific and functional relevant (Table E10, page 14).

These additional analysis and experiments are now included in our revised manuscript. We believe that these studies further improve the significance of our manuscript.

© European Molecular Biology Organization

Specific point:
1) What fraction of the TFs is expressed in the cell line used here (HEK)? This should be mentioned, as many expression data are now a day available, and the possible impact of expression in a "heterologous" system should at least be discussed.

We have added a TF expression profile in HEK293T cells in the revised Table E1, sheet 2. We have expanded our discussion on the "heterologous" system in the Discussion (page 18). In general, since proteins often form distinct protein complexes in different tissues to execute their tissue-specific functions, our interaction and functional prediction as well as disease correlations based on the TAP/MS results performed in HEK293T cells only represent a fraction of the functions carried out by these bait proteins and should only be used as references. One needs to be cautious when drawing any conclusion solely based on PPI studies.

Reviewer #2:

In this manuscript the authors describe an analysis of transcription factors and their protein interactions in HEK293C cells. The authors analyze 56 transcription factors with a particular focus on forkhead box family transcription factors and 19 additional transcription factors that are largely independent of the forkhead family transcription factors. The authors generate stable cells lines of these transcription factors with a tandem affinity tag and they analyze the soluble and chromatin associated fractions of each transcription factors protein interactions. The authors use standard and sound approaches to analyze their protein interaction network data and they compare their data to prior knowledge and they make the case for finding many new interactions in their datasets. Overall, the work is properly carried out and analyzed. However, there are several significant issues that need to be addressed prior to publication.

Thank you for the nice summary. Please see below for details that address the issues you raised.

The first major issue is the samples used. The major focus is on the forkhead box family of transcription factors and there is potentially novel biology of importance in this data. Focusing the manuscript on this data only would strengthen the manuscript. The 19 additional transcription factors come across as an afterthought and do not add much to the manuscript. This aspect of the manuscript needs to be reconsidered. The authors carried out reciprocal purifications (data in Fig 2 F) but end with this. NFATC1 for example appears interesting in that it pulls down several transcription factors. A discussion of this and validation by CoIP or colocalization with imaging would be valuable and one way to strengthen this aspect of the manuscript. The other issue with the additional 19 TFs studied is some of these are well studied and there are missing identifications. For example, RelB is not listed in the NFKB1 datasets. What information was missing from their analysis and why?

Thank you for the suggestion. We agree that the 19 additional transcription factors were not included at the beginning of our study, and our functional validations mostly focused on FOX proteins. However, we believe that these 19 TFs provide additional information for our study. First, they encompass all of the five structural superfamilies of TFs, which supports our conclusion of distinct TF complexes formed on and off chromatin beyond just FOX TFs. Second, these additional TFs are involved in many human diseases and signalling pathways. We hope that the profiling of their interactomes could provide useful resource for the research community.

We agree with the reviewer that additional data validation and discussion on these 19 TFs are necessary. We have expanded our studies on the JUN-CREB-ATF-NFATC sub-network. Based on our purification results, we built this fraction-specific sub-network, in which NFATC only binds to JUN-CREB-ATF complex in chromatin fractions (Fig. 6A, page 13-14). We have confirmed the interactions between NFATC1 and other TFs by co-IP using epitope tags (Fig. 6B, 6C, page 14). We have also compared the ChIP-seq results of NFATC1 with those of ATF2 and CREB1 that are available in the ENCODE database and found significant overlapping between their target genes (Fig. 6D, page 14). These data indicate that they indeed function together to regulate gene expression (Fig 6, page 13-14).

© European Molecular Biology Organization

Yes, we have identified REL and RELA, but not RELB, in our NFkB1 purifications. RELB was identified as NFkB1-associated protein in a similar TAP/MS study conducted by Dr. Superti-Furga's group using HEK293 cells (Bouwmeester et al. 2004). Thus, we repeated our NFkB1 purification using the same stable cells with increased MS detection time, which allowed us to increase total peptide and protein identification by about 2 folds (total spectra counts: 2054 versus 1125, total protein identifications: 138 versus 58) and obtained 4 peptides of RELB in NFkB1 soluble fractions, indicating that this association is true but relatively weak (new purification result of NFkB1 has been included in Table E12). This result is consistent with NFkB1 TAP/MS result performed in HEK293 cells as previously reported by Dr. Superti-Furga's group, in which they identified RELB 4 times in their 7 NFkB1 purifications. Again, we believe our TAP/MS results performed in HEK293T cells only represent a fraction of the binding proteins and functions carried out by these bait proteins, which is limited by cell type and MS sensitivities. The current study only reflects a snapshot of the complexity of human protein interactomes. We have added this discussion and reference in the Discussion section (Page 15).

Next, for all the purifications there are several instances where no identifications were presented in Fig3 like for FOXC4 chromatin, FOXC2 soluble, FOXJ2 soluble, ETS1 chromatin. The authors must discuss these samples where no identifications are provided and provide an explanation for this. Were there technical issues with their approach for these samples and if so what were they, or is there a biologicsal reason for these results? This must be discussed for their method to be fully understood by researchers.

The results that have few or no prey identifications are due to several reasons. In many cases, such as in the cases of FOXC2 and FOXJ2, the bait proteins expressed well and could be found in both fractions. For example, we identified 38 and 64 peptides of FOXC2, 129 and 100 peptides of FOXJ2 in chromatin and soluble fractions, respectively. However, after the removal of non-specific interacting proteins such as chaperones, there is no HCIP left in the soluble fraction lists, which may indicate that these proteins only form functional complexes on chromatin. In some other cases, such as FOXA2 and ETS1, the bait proteins are highly enriched in one fraction. For example, we identified 20 peptides of FOXA2 in chromatin fraction, but none in the soluble fraction. Similarly, we identified 59 peptides of ETS1 in chromatin fraction and 402 peptides in soluble fraction (also see Fig. 6B, ETS1 is highly enriched in soluble fraction). In these cases when the bait protein was predominantly presented in one fraction, the lack of HCIPs in the other fraction could reflect the nature of these bait TFs, which predominantly form functional complexes in one fraction. Of course, this could also due to technical reasons, especially for chromatin fractions, since we may lose some of the associated proteins during extraction of chromatin-associated proteins. In the case of FOXO4, the failure to identify HCIPs could be just due to technical issues, since we only recovered a few peptides of FOXO4 in either fractions, which may indicate problems with protein expression or stability. We have added this discussion in the revised manuscript (page 15).

The authors then largely focus on the forkhead box TFs and a couple of insights they found in their data. One is on a GO analysis of functions of preys for FOXM1 and the differences in the soluble and chromatin associated proteins for this TF. This data is interesting but no validation of the results is provided to show FOXM1 plays a role in the tRNA splicing complex function or regulation, for example. As it stands, this is a potential role of FOXM1, but remains speculative.

We agree that we cannot make any solid conclusion solely based on interactomes and GO analysis. We have therefore toned down our predictions as suggested (page 12).

The authors then provide CoIP information on FOXN2 and its interaction with FBXW11. The authors provide some data to suggest that there may be an increase in degradation upon these interactions in in Fig 5E, but this data is preliminary and more validation is needed to strengthen this aspect of the manuscript. Carefully considering the literature on FBXW11 and CUL1 and better demonstrating their ability to regulate FOXN2 would be valuable. For example, does RNAi of either of these proteins, or others in their pull down affect cellular FOXN2 levels? Additional evidence on FOXN2 and its regulation would strengthen the manuscript. As it stands, this is a well executed analysis of transcription factor protein interactions, but the validation of these results and the potential new biological insights is lacking for a journal like Molecular Systems Biology. In addition to the other revision described, strengthening the data on FOXN2 would improve the paper sufficiently.

We agree that additional validation of FOXN2 HCIPs will be very helpful in supporting our conclusions. Therefore, we have now compared FOXN2 stability in HEK293T cells and HEK293T-shβTRCP/ βTRCP2/CUL1 cells. In support of our working hypothesis that βTRCP/βTRCP2/CUL1 complex is involved in FOXN2 degradation, we found that FOXN2 became more stable in cells with CUL1 downregulation (Fig. 5G, page 13). Increased FOXN2 stability was modest in cells with βTRCP or βTRCP2 knockdown (Fig. 5G, page 13), which agrees with the fact that βTRCP and βTRCP2 are highly related proteins and have overlapping functions in the cell. We also performed ChIP-seq experiments of FOXN2 and its chromatin-binding partner RFX1 in 293T cells, with two biological and two technical replicates each and four negative controls. The results showed that 44% FOXN2 target genes overlapped with RFX1 target genes, indicating that they indeed function together on chromatin to regulate gene expression (Fig. 5C, page 13). We hope that these additional validations, together with our previous data, support our hypothesis of fraction-specific complex formation involved in the regulation of transcription factors.

Reviewer #3:

This manuscript describes the protein-protein interaction network of a large number of transcription factors (TFs) identified by modified tandem-affinity-purification followed by mass spectrometry (TAP/MS). The authors obtained proteomics data of soluble and chromatin associated complexes of the 56 TFs which include targets of many signalling pathways and a complete family of Forkhead (FOX) TFs (actually only 37 members so the text needs altering). More importantly, they concluded that most of TFs form very distinct protein complexes on and off chromatin, and the location-specific protein complexes are associated with distinct regulatory properties and diverse functions of these TFS. Overall, this is a potentially useful dataset but to be useful, there are some important areas of validation required (see below) to demonstrate its robustness. If the authors want to go beyond presenting a dataset and provide novel mechanistic insights, then further experimentation is required to validate things like "location-specific complexes". If these experiments are not provided, then the title needs altering and some of the conclusions toned down.

Thank you for the careful reading of our manuscript and summary of our studies. Please see our point-to-point response below for details.

Major comments:
(1) More evidence needs providing to validate the subfractionation methodology. For example, western blots showing the correct re-distribution of factors expected to be in the soluble and insoluble fractions. This is important so that we know exactly which parts of the cell we are dealing with. Indeed, the authors comment that they do not get a lot of histones and HMG proteins. Why are these not in the "nuclear fraction" as one might expect, and does this mean that there is another fraction that is discarded which might have TFs associated?

As expected, most of the TFs mainly localize in the nucleus. Therefore, in this study, we did not isolate cytosolic versus nuclear fractions. Instead, we isolated soluble versus chromatin fractions based on our assumption that TFs on chromatin are likely to be involved in transcription-related functions, while they are not when in the soluble fractions. The soluble fractions of these TFs are still localized in the nuclei (as determined by immunostaining). We agree with this reviewer that an example of re-distribution would greatly strengthen the validity of our fractionation strategy. Thus, we treated HEK293T cells with PI3K inhibitor LY294002 and observed FOXO3 re-distribution using IF (Fig. E3F) and WB (Fig. E1D).

For the extraction of chromatin fractions, we treated the insoluble fraction with TurboNuclease, which hydrolyses both single- and double-stranded DNA or RNA to oligonucleotides of 1-4 bases in length, to release chromatin-bound proteins. In fact, this step released all the histones and HMG proteins to the “chromatin fraction”, which has been confirmed by WB (please see Fig. 1C, in which Histone H3 is only detected in chromatin fraction. We have also blotted for Histone H4 and HMGA2 antibodies and the results were the same). This TurboNuclease treatment step not only released the chromatin-bound proteins, but also eliminated any nonspecific interactions mediated by DNA. Only specific interactions between TF and certain chromatin-associated proteins would be identified by our stringent purification and data filtration. We did get histone components in many

© European Molecular Biology Organization

of our purifications but the abundance was usually low (Table E3). These low abundant TF-histone interactions were considered as non-specific interactions and eliminated by our modified-SAINT analysis, while specific interactions remained after data filtration. For example, RBPJ co-purified with the entire HDAC1/2 complexes and HMG20B, but not with other abundant HMG proteins such as HMGA1/2 (Table E6). Thus, we believe our fractionation strategy is effective and reflects the specific protein-protein interactions on chromatin. TFs probably do not always associate tightly with histones or HMG proteins. This is likely the reason that we did not regularly uncover these proteins in our HCIP dataset from tandem-affinity purifications.

(2) There is a lack of critical validation experiments. While it is impractical to do for everything, some examples should be given. For example, with the available antibodies, it would be relatively easy to do for FOXM1, to show first by mass spec that the same proteins are found. Secondly western analysis with antibodies to endogenous proteins would help. This is important as the authors admit, tags can affect things, as can over-expression (although in several examples shown, the authors attempt to choose lines not vastly over-expressing TFs).

Thank you for the suggestion. We agree that using tags and over-expression could potentially cause artifacts in affinity purifications. Thus, we chose the stable lines with the correct sub-cellular localizations and the lowest expression for affinity purifications, and used stringent criteria in both purification and data analysis, to minimize the artifacts and try to best reflect the interactions under physiological conditions.

Yes, we have compared our TAP-MS results with our previous purification results using antibodies against endogenous proteins (Malovannaya et al. 2011) (Table E6, column K). With the baits available in that dataset (JUN, MAX, NFkB1, SMAD4, TP53, FOXK1, FOXK2, FOXP1, FOXP2 and FOXP4), 37% high-confident interactions identified by our TAP-MS are also present in that endogenous AP-MS dataset.

To further confirm that the fraction-specific interactions truly reflect the status of endogenous protein complexes, we have now conducted AP purifications using antibodies against endogenous JUN (Cell Signaling #9163S) in chromatin and soluble fractions (Table E9, Fig. 6A, page 13). 65% high-confident interactions identified by our tagged TAP-MS have been confirmed by these AP-MS using antibodies against endogenous JUN. More importantly, we found the fraction specificities are highly reproducible by endogenous AP. FOS, FOSB, ATF2, ATF3, ATF7 and CREB5 were found in both fractions, while NFATC1, MEF2A, HOXB9, SMARCB1 only appeared in chromatin fractions, and DARS, IARS, NBR1 only in soluble fractions. 96% JUN high-confident interactions confirmed by AP using endogenous antibodies have the same fraction preference as those in TAP-MS results. These data support our conclusion that this fraction-specificity of TFs is not due to the epitope tags or overexpression, but reflects the fraction preference of endogenous protein complexes.

(3) Validation of the different locations by an alternative means is required. For example PLA could be used to show cytoplasmic versus nuclear interactions of endogenous proteins. ChIP can be used to show co-occupancy on chromatin.

We agree with this reviewer's comment. As mentioned above, we treated HEK293T cells with PI3K inhibitor LY294002 and observed FOXO3 re-distribution by IF (Fig. E3F) and WB (Fig. E1D).

We agree that additional validation of FOXN2 HCIPs will be very helpful in supporting our conclusions. Thus, we performed ChIP-seq experiments of FOXN2 and its chromatin-binding partner RFX1, with four experiments each (two biological and two technical replicates). The results showed that 44% FOXN2 target genes overlapped with RFX1 target genes, indicating that they indeed function together on chromatin to regulate gene expression (Fig. 5C, page 13). We believe that these additional validations, together with our previous data, support our hypothesis that fraction-specific complex formation are involved in the regulation of transcription factors.

We have also compared the ChIP-seq results of NFATC1 with those of ATF2 and CREB1 that are available in the ENCODE database and found significant overlap between their target genes (Fig. 6D). These data indicate that these TFs indeed function together to regulate gene expression (Page 13-14, Fig 6).

© European Molecular Biology Organization

Related to this point, there is no validation of whether the "soluble" interacting proteins affect TF activity, or indeed any of the new interactors (with the exception of minimal evidence to show low levels of polyubiquitination of FOXN2 when a protein is over-expressed).

We have now compared FOXN2 stability in HEK293T cells and HEK293T-shβTRCP/βTRCP2/CUL1 cells. We found that FOXN2 became more stable in cells with CUL1 downregulation (Fig. 5G, page 13), while the increased stability of FOXN2 was modest in cells with βTRCP or βTRCP2 knowdown (Fig. 5G, page 13), which is in agreement with the fact that βTRCP and βTRCP2 are highly related proteins and have redundant functions in the cell. These data further support our hypothesis that fraction-specific complex formation are involved in the regulation of transcription factors. In this case, the soluble βTRCP/βTRCP2/CUL1 complex is involved in FOXN2 degradation.

Specific comments for the figures
1) Fig 2C could be displayed as a supplementary table.

We have moved the results in Fig. 2C and 2F to two new tables (Table 1 and 2).

2) Fig. 2D is confusing. As described in the text on page 8, the highest reproducible rate from the 24 purifications (20 % of total 120) of biological replicates is 10 counts with the lowest 44% of 2 counts. How can it be that the unreproducible rate is therefore only 10% as shown in the figure 2D?
To avoid the confusion, we removed the pie chart and only presented the bar graph to show the reproducibility of our results.

3) In Fig.2E the majority of the HCIPs identified by the authors are unknown compared to several known databases. However, this could be due to the lack of in depth studies. Can the authors therefore select very well documented TF and compare the interaction proteins with the data obtained in this manuscript? This will help with validating the data.

Thank you for the suggestion. We agree with this reviewer and believe that this is the case. We have compared the results from 19 relative well-studied TFs with the information available from the knowledge database (new Fig 2D, right panel). Of these 19 TFs, 33% of the interactions we identified in this study have already been reported by previous studies, which is higher than the average 18% of all interactions in our study. In some cases, such as NFkB1 and Smad4, this rate is even higher (100% and 84%, respectively).

4) In Figs E3, E4, the data is not that informative. Nearly every TF in either fraction brings up the terms cell cycle and development, so this does not really discriminate well (see comments about FOXM1 below). Can the authors point to more informative terms that would help with our understanding of individual TF function?

We agree with this reviewer that the GO and disease correlation interpreted by HCIPs may not be very specific and informative. Thus, we have removed the previous Fig. 6C and Supplementary Figs. E3 and E4. We have added a TF-canonical pathway enrichment table (Table E10), which indicates the correlation of TF functions predicted by their HCIPs with canonical pathways. These suggest pathway involvement of not only bait TFs, but also the preys, as listed in the column E. We hope that this information could be more specific than the regular GO annotation on molecular functions and diseases.

5) In the description of Fig.3B, the authors pick and choose things to suit their viewpoint. There are many other proteins that do not fit. For example, MEF2A is clearly nuclear enriched yet it shows peptides that are apparently soluble enriched. The reciprocal applies to NFATC1 which has chromatin enriched peptides but is exclusively cytoplasmic. The data description therefore needs radically altering and some explanation provided. In this regard, FOXN2 appear completely nuclear yet in Fig.5, it seems to only be binding to soluble proteins. Are these soluble nuclear or soluble cytoplasmic?

Sorry for the confusion in the text. As expected, most TFs predominantly localize in the nuclei, which are also the cases for their tagged counterparts. In this study, we did not isolate cytosolic

versus nuclear fractions. Instead, we isolated soluble versus chromatin fractions based on our assumption that TFs on chromatin are likely to be involved in transcription-related functions, while they are not when in the soluble fractions. The soluble fractions of these TFs are still localized in the nuclei (as determined by immunostaining). For example, MEF2A localizes in the nucleus as indicated by immunostaining (Fig. E1A). However, MEF2A mainly present in the soluble fraction but not on the chromatin, as suggested by fractionation and Western blotting analysis.
We also found that the numbers of HCIPs (as shown in Fig.3) do not correlate with the bait protein expression levels in different fractions. For example, FOXN2 and FOXJ2 are present in both fractions. We identified 54 and 113 peptides of FOXN2, 129 and 100 peptides of FOXJ2 in chromatin and soluble fractions, respectively. After the removal of non-specific interacting proteins, there are few or no HCIPs in the chromatin fraction of FOXN2 or the soluble fraction of FOXJ2. These data again support our hypothesis that TFs form very distinct complexes on and off chromatin.

6) In Fig.4D-F. According to the figure, FOXM1 binds HCIP on chromatin mainly functioning in cell cycle regulation, whereas in the soluble fraction, the top GO is 'RNA post transcriptional modification'. However, in the text in page 15, the authors mentioned that '...FOXM1 formed two distinct complexes on and off chromatin, but for the same biological outcome: the regulation of mitosis and the promotion of cell cycle progression'. This is misleading (See point 4 above).
In Figure 4F, for the HCIP in soluble fraction, it is indicated as 'transcription independent function' but this is likely that this might only be regulation or modification for the bait protein itself, instead of the function of the FOXM1 protein. If the authors want to invoke new functions for FOXM1, then they should show that it affects tRNA splicing.

We agree that we cannot draw any solid conclusion solely based on interactomes and GO analysis. Therefore, we have toned down our conclusion (page 12).

7) It is interesting to identify the interaction proteins for FOXN2, however, they need more evidence to clarify the conclusion. In Fig.5B, to confirm that both FOXN2 and RFX1 interact on chromatin, ChIP or even re-ChIP should be performed. For immunopricipitation, ideally, endogenous proteins should be detected. Chromatin fractionation experiments should be performed in some validation experiments.

Thank you for the suggestion. We have performed the ChIP-seq and CHX assays as suggested. For details, please see our response to your general point 3.

8) Fig 6C - Based on the identified protein-protein interaction data, the authors created a new disease correlation network, which is quite different from the one shown in Fig 1A, see Ets1, TP53 and Max etc. This will cause confusion and misunderstanding for the potential function TFs, and it is not clear that interacting proteins (rather than regulated genes) will provide a useful readout of biological function/disease association (see other comments above about generally useless GO terms such as cell cycle and development).

This disease network only shows the new correlations, in which we removed the “known correlations” to emphasize the new insights from our data analysis. In fact, over 70% previous reported disease correlations have also been suggested by our analysis. Nevertheless, we agree with this reviewer that the GO and disease correlation interpreted by HCIPs may not be very specific and informative. Thus, we have removed the previous Fig. 6C and Supplementary Figs. E3 and E4.

Other minor and textual changes:
(1) Did the authors use any stimuli to make TFs move to the nucleus like NFAT or NFkB etc which are clearly predominantly cytoplasmic? If not, then what is the relevance of the nuclear fraction for these TFs? Careful presentation and discussion of this point is needed.

We have used stimuli for some of these TFs, including Smad4 (TGFβ and BMP4), TCF4 (LiCl), JUN/FOS (TNFα), to promote their nuclear localization. We have observed slight increase of bait spectra counts in the chromatin fractions except TCF4 (Table E6). In the revised manuscript, we have also used PI3K inhibitor LY294002 to promote FOXO3 nuclear localization and redistribution to chromatin fractions (Figs. E1D, E3F).

(2) On page 9, the text on MAX/FOXM1 interactions is not clear. Did you tag all the interactors and then flip the experiment or did you just repeat the MAX/FOXM1 IPs? Where is this data?

Yes, we tagged all the MAX/FOXM1/FOXO3 HCIPs with SFB tag and performed reverse Co-IP experiments, and blotted with antibodies against endogenous MAX/FOXM1/FOXO3 (please see Supplemental Figure E3).

(3) The font size in Fig. 3A will be impossible to read in the final sized paper figures.

Thank you for the suggestion. We have increased all the font sizes from 6pt to 8pt in the revised Fig. 3A.

(4) Page 10. In addition to Sadisvam et al., 2012, there are two more papers that should be cited that link FOXM1 to the MMB complex. Grant et al. Mol Biol Cell. 2013 Dec;24(23):3634-50. Mol Cell Biol. 2013 Jan;33(2):227-36.

Thanks! We have added these references in the revised manuscript.

(5) Different colours for the lines in Fig. 6C should be selected for the benefit of colour blind scientists.

We have removed this panel as suggested. Please see our response to your specific point 8.

(6) On page 3, the authors should cite a paper from the Taipele lab which identifies PWMs for all Forkhead TFs. Cell. 2013 Jan 17;152(1-2):327-39.

This reference is now included in the revised manuscript.

2nd Editorial Decision 17 November 2014

Thank you again for submitting your work to Molecular Systems Biology. We have now heard back from the three referees who agreed to evaluate your manuscript. As you will see from the reports below, the referees are satisfied with the modifications made and they think that the study is now suitable for publication.

Prior to the formal acceptance of your manuscript, we would ask you to address the following points:
- We would like to ask you to deposit the newly generated ChIP-Seq datasets (for FOXN2 and RFX1) in one of the appropriate public databases. (Additional information is available in the "Guide for Authors" section in our website at <http://msb.embopress.org/authorguide#a3.5>) and to include the links and accession numbers in the "Data Availability" section of your manuscript.
- We would also like to encourage you to provide the Cytoscape files (as supplementary files) for the figure panels depicting related network illustrations.
- Along the lines of the comment by Reviewer #3 regarding the title, we would like to suggest the following title: "Proteomic analyses reveal distinct chromatin-associated and soluble transcription factor complexes".

Please resubmit your revised manuscript online, with a covering letter listing amendments and responses to each point raised by the referees. Please resubmit the paper **within two weeks** and ideally as soon as possible. If we do not receive the revised manuscript within this time period, the file might be closed and any subsequent resubmission would be treated as a new manuscript. Please use the Manuscript Number (above) in all correspondence.

REFEREE REPORTS

Reviewer #1:

© European Molecular Biology Organization

The authors have adequately addressed the points and questions that were raised.

Reviewer #2:

The authors have significantly and appropriately revised this interesting body of work. I fully support publication of the revised manuscript.

Reviewer #3:

This is a much improved paper and the authors have addressed the majority of my comments well. The important issues were validation of the data and providing more biological relevance, and this has now been done. In terms of a dataset, this is now reasonably well validated and should be of use to the community. I only have a few minor points to make. First, the authors have not apparently treated cells with anything to make NFAT and NFkB translocate to the nucleus. Ideally this should be commented on and how they can then detect nuclear complexes. Secondly, the authors do not really provided any novel functions for the "soluble" complexes. All they show is that a protein is targeted for degradation in this fraction. ie no new function is ascribed to the transcription factor in this context. Some rewording is therefore warranted.
The other issue still surrounds the title and conclusions based on "location specific" complexes. What the authors mean is chromatin bound or not, rather than "location" per se. this is a little bit semantic but could still be confusing for the reader. Also in this regard, showing know translocation of FOXO to the nucleus does not help, and protein complexes should have been shown by WB in chromatin and nuclear soluble fractions.
At this stage, I would suggest altering the title to something like "Chromatin associated and chromatin free complexomes for transcriptional regulation" to make the message clearer.

2nd Revision - authors' response 25 November 2014

Specifically, we have deposited our ChIP-seq data of FOXN2 and RFX1 to ArrayExpress under accession number E-MTAB-3120. To access the data please visit: http://www.ebi.ac.uk/arrayexpress/experiments/E-MTAB-3120/. For reviewer login please use: Username: Reviewer_E-MTAB-3120, Password: W8KK4Wvj. The link and the accession number are also presented in the Materials & Methods section (page 23).

We have included Cytoscape files illustrating TF protein interaction networks in chromatin and soluble fractions as supplementary dataset S1 (page 9).We have modified our title and abstract following the suggestions by you and Reviewer#3. We have also revised the text to include additional comments and rewording as suggested by Reviewer#3.

© European Molecular Biology Organization
